# Proteogenomic Assessment of Intraspecific Venom Variability: Molecular Adaptations in the Venom Arsenal of *Conus purpurascens*

**DOI:** 10.1016/j.mcpro.2021.100100

**Published:** 2021-05-23

**Authors:** Meghan Grandal, Mickelene Hoggard, Benjamin Neely, W. Clay Davis, Frank Marí

**Affiliations:** 1 Chemical Sciences Division, National Institute of Standards and Technology, Hollings Marine Laboratory, Charleston, South Carolina, USA; 2 Department of Drug Discovery, Medical University of South Carolina, Charleston, South Carolina, USA

**Keywords:** proteogenomics, venomics, cone snails, *Conus purpurascens*, venom variability, AGC, automatic gain control, HCD, high collision dissociation, LC-MS/MS, liquid chromatography coupled to tandem mass spectrometry, nAChR, nicotinic acetylcholine receptor, NGS, next generation sequencing, PTM, posttranslational modification, TIC, total ion chromatogram

## Abstract

Cone snails produce venom that contains diverse groups of peptides (conopeptides/conotoxins) and display a wide mass range, high rate of posttranslational modifications, and many potential pharmacological targets. Here we employ a proteogenomic approach to maximize conopeptide identification from the injected venom of *Conus purpurascens*. mRNA sequences from *C. purpurascens* venom ducts were assembled into a search database and complemented with known sequences and *de novo* approaches. We used a top-down peptidomic approach and tandem mass spectrometry identification to compare injected venom samples of 27 specimens. This intraspecific analysis yielded 543 unique conopeptide identifications, which included 33 base conopeptides and their toxiforms, 21 of which are novel. The results reveal two distinct venom profiles with different synergistic interactions to effectively target neural pathways aimed to immobilize prey. These venom expression patterns will aid target prediction, a significant step toward developing conotoxins into valuable drugs or neural probes.

## Highlights

• We analyzed the injected venom of 27 specimens of *Conus purpurascens*.
• We found 543 unique conopeptide identifications.
• We identified 21 novel base conopeptides.
• We found two distinct venom profiles with different synergistic interactions.

## Graphical Abstract

**Figure:**
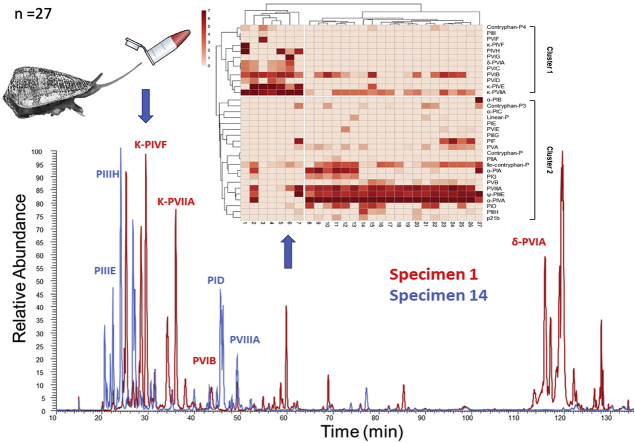

## In Brief

Cone snail venom is an extensive source of active molecules that have potential pharmacological and biotechnological applications. We employed a top-down functional proteogenomic approach to assess the injected venom of *Conus purpurascens*. The two distinct venom profiles found will interact differently to target neural pathways aimed to immobilize prey. These venom expression patterns will aid target prediction and the development of conotoxins into drug leads or neural probes.

Venomous animals comprise over 200,000 species across several taxa and display a variety of mechanisms for venom production, delivery, and use (1). For most animals, venom is proteinaceous in nature; however, as different taxonomic groups independently evolved, they developed highly adapted and very specific venom concoctions as a solution to environmental pressures, a clear example of convergent evolution. Most venoms are complex mixtures of peptides, proteins, and small molecules that might act in concert to immobilize prey or deter predators. The specific molecular content of these composites varies from phyla, class, order, family, and genus. There can be also significant venom variability within the same species (2, 3, 4, 5, 6, 7, 8, 9, 10). In some cases, venom varies within the individual specimens (3, 11, 12, 13, 14, 15), as some animals can switch their venom from predatory to defensive concoctions. Intraspecific venom plasticity expands the molecular adaptations of venomous animals, and in doing so it augments the repository of compounds with medicinal applications, such as Captopril from the Brazilian pit viper venom, Exenatide from the Gila monster, and Ziconotide/Prialt from cone snail venom (16).

The venom found in marine predatory snails belonging to the genus *Conus* (cone snails) has been intensely studied in terms of content and pharmacological properties. Most notable are the conotoxins, a diverse group of disulfide-constrained (two or more disulfide bonds) peptides that target ion channels, ligand-gated receptors, and transporters with high affinity and specificity (17, 18). *Conus* venom can also contain linear and one-disulfide bond peptides (19), which along with conotoxins define the conopeptides, the full small peptidic complement of the venom of cone snails. Conopeptide diversity occurs at both the sequence and posttranslational modification (PTM) level, resulting in thousands of conopeptides that range in size, structure, and activity. Conopeptides/Conotoxins are classified according to gene superfamilies based on conserved signal sequences; and each superfamily can encode hundreds of mature conopeptide sequences (20, 21, 22). Mature conotoxins have displayed a plethora of cysteine frameworks and disulfide-bonding patterns, which in turn affects activity. Conopeptide complexity also results from a high rate of PTMs (23, 24, 25). The same base peptide can have many differentially modified forms (26, 27), or “toxiforms”. Conopeptide hypermodifications can be viewed as an evolutionary development to expand the molecular reach of the venom.

The molecular diversity of cone snail venom is extraordinary as its expression is species-specific with little overlap of components among the more than 800 extant species (28). This complexity is compounded by intraspecific and intraspecimen venom variations due to predatory or defensive venom profiles (10, 11, 13). This complexity provides a rich source of bioactive peptides (29, 30), but it also presents a challenge for venom characterization. Intraspecific studies have relied heavily upon comparisons of venom chromatography and mass matching to known venom components, rather than global MS/MS spectral matching, to identify venom components. Because one base conopeptide can have many toxiforms with different masses, it is very difficult and rather uninformative to assess intraspecific venom variation through molecular mass lists alone, and in doing so, it can lead to overestimates of the extend of venom variability. Next-generation sequencing technology for RNAseq and advances in high-resolution liquid chromatography coupled to tandem mass spectrometry (LC-MS/MS) have mitigated the challenges associated with the analysis of complex venoms and have allowed assessment of the venom peptidome/proteome through “venomic” approaches (31, 32).

A comprehensive analysis of the venom composition is crucial to assess venom plasticity and to determine synergistic mechanisms of envenomation used to immobilize prey or deter predators. Here we present a large-scale intraspecific venom analysis of *C. purpurascens*, the only fish-hunting species of the tropical Eastern Pacific region. Earlier groundwork revealed that *C. purpurascens* had two distinct venom “cabals” or groups of conopeptides acting synergistically to paralyze their prey (9, 33, 34). The cabals act as either (1) a neuromuscular block (motor cabal), targeting nicotinic acetylcholine receptors (nAChRs, α- and ψ-conotoxins) and skeletal muscle sodium channels (μ-conotoxins), or (2) an excitotoxic neuronal block (lightning-strike cabal), targeting neuronal sodium (δ-conotoxins) and potassium channels (κ-conotoxins). Previous works, however, were based on mass lists obtained from the venom of a limited number of specimens (3, 9).

We present a comprehensive venom analysis by utilizing high-resolution LC-MS/MS-based peptide identification to analyze and compare injected venom from 27 individual specimens of *C. purpurascens*. In doing so, we sought to maximize the identifications of conopeptides and their toxiforms. We also assessed the biochemical diversity of the venom arsenal by comparing conopeptide expression patterns to gain a more refined view of synergistic relationships among the venom components.

## Experimental Procedures

### Animal Specimens and Venom Collection

Specimens of *C. purpurascens* (n = 27) were collected from a 4 km^2^ area on the Guanacaste Province, Pacific coast of Costa Rica. Their size ranged from 50 to 85 mm in length. Snails were kept in an aquarium facility at 25 °C with a day/night cycle where they were “milked” and fed every 15 days in average. *C. purpurascens* was chosen for this intraspecific venom analysis because it is a fish-hunting species that uses a hook-and-pull strategy to capture the prey allowing venom collection through a “milking” procedure (35). Briefly, venom was collected into Eppendorf tubes that have a piece of latex glove stretched over the opening and are baited with a piece of goldfish fin on the latex. When the snail senses the fin, it spears the latex and injects venom into the tube. After the venom is released, the snail is fed with a live fish. The injected venom samples were not pooled, and they were stored at –80 °C until used for further analysis. We used injected venom as it differs in composition and complexity from venom that is collected by dissecting the snail and extracting from the gland (5, 36) and is a more accurate representation of what is injected into their prey. Sizes of the individual specimens are provided in supplemental Table S1.

### Reduction/Alkylation and LC-MS/MS Analysis

An aliquot of individual venom sample (5 μl) was diluted in ammonium bicarbonate buffer (50 mM). Cysteine bonds were reduced with dithiothreitol (7 mM) for 1 h at 60 °C and alkylated with iodoacetamide (18 mM) for 1 h at 21 °C in the dark. Following reduction and alkylation, the samples were desalted using C18 spin columns (ThermoFisher Pierce) and lyophilized before LC-MS/MS analysis.

Samples were reconstituted in water/0.1% formic acid and analyzed by LC-MS/MS on an Orbitrap Fusion Lumos trihybrid mass spectrometer (Thermo Fisher Scientific) coupled with an UltiMate 3000 RSLCnano System (ThermoFisher Scientific). A 160 min gradient with solutions A (5% acetonitrile/0.1% formic acid) and B (80% acetonitrile/0.1% formic acid) on an Acclaim PepMap 2 μm C18 column (75 μm × 25 cm) (Thermo Fisher Scientific) was used. The flow rate was set at 0.3 μl/min with the following gradient steps: 0 min at 5% B, 10 min at 5% B, 115 min at 27.5% B, 130 min at 40% B, 140 min at 95% B, 150 min at 5% B, 160 min at 5% B.

MS1 scans (200–2000 m/z) were collected with the Orbitrap mass analyzer at a resolution of 120,000 using quadrupole isolation; RF lens 30%, AGC target 4.0e^5^, and a 50 ms injection time. Precursor ions were fragmented using HCD (32%). MS2 scans were collected with the Orbitrap mass analyzer at a resolution of 30,000 using quadrupole isolation and AGC target 2e^4^. A charge state filter was used (+2–6) and the intensity threshold was set to 2e^4^. Dynamic exclusion was set to exclude precursor ions for 60 s after collecting 10 MS2 scans within 30 s.

### Transcriptome Assembly and Database Configuration

Subsequent to the collection of their corresponding injected venom, two *C. purpurascens* specimens (both female and 41 and 55 mm in length respectively) were sacrificed for transcriptomic analysis. The venom ducts were dissected from the snails, immediately placed in RNAlater (Invitrogen) and stored at –80 °C. mRNA was extracted from the venom gland using an RNeasy Lipid Tissue mini kit (Qiagen), and mRNA quality was confirmed with a 2100 Bioanalyzer (Agilent). Illumina libraries were prepared with an NEBNext Ultra Directional RNA Library prep kit (New England BioLabs). Sequencing was performed on a NextSeq 500 platform (Illumina, Inc.) and yielded 28 million paired-end reads (75 bp). The two transcriptomes were assembled with Trinity *de novo* transcript assembler (v. 2.2.0) using default parameters; group pairs distance: 500 bp, path reinforcement distance: 75 bp (37). Both transcriptomes were translated with EMBOSS applications, transeq (6-frame) and getorf (between the start and stop codons), for analysis and comparison (38, 39).

Several databases were configured and assessed for completeness before choosing the best search database for the 27 venom samples. The database was optimized for time-intensive nonenzymatic searches with many PTMs using the following criteria; inclusivity of conopeptide-encoding transcripts and the total number of entries. We compared the following four database configurations, all from the *de novo* transcriptome assemblies of venom gland RNAseq data. (1) The *de novo* assembly was searched against the UniProt Animal Toxin Annotation database (ToxProt) and all UniProt *Conus* entries (TaxID: 6490) using blastX (e = 10^−5^), then the translated open reading frames were extracted with getorf, and complete transcripts with signal sequences were extracted with SignalP v4.0 (40). (2) The *de novo* assembly was searched as previously described using blastX, then the hits were translated with transeq, and only transcripts containing >4 cysteines were extracted. (3) The *de novo* assembly was translated, the open reading frames were extracted with getorf, and complete transcripts with signal sequences were extracted with SignalP (this configuration did not include a blast step). (4) Trinity assembly was translated with transeq and getorf, and resulting transcripts were searched against the ToxProt database to extract toxin-like sequences using blastp (e = 10^−5^). We chose the ToxProt-guided configuration (4) as the optimal search database, to which we added a customized *C. purpurascens* database that included conopeptide sequences not present in the transcriptomes (supplemental Fig. S1). The additional *C. purpurascens* database included previously identified peptides from UniProt (taxid: 41690) and unpublished conopeptide sequences identified in-house using the PEAKS *de novo* search algorithm (Bioinformatics Solutions Inc) (41). PEAKS can deduce peptide sequences from MS/MS spectra without a database. PEAKS scored the predicted sequences with an average local confidence (ALC) score. We only included predicted conopeptide sequences with ALC scores greater than 98% in our in-house *C. purpurascens* database (supplemental Fig. S2).

### Database Search Parameters and Acceptance Criteria for Identifications

Database searches were performed with the Sequest algorithm within Proteome Discoverer v2.0 (Thermo Fisher Scientific). Two databases were included in the search for the 27 venom RAW files (supplemental Table S2): (1) Tox-Prot-guided database (1652 entries) and (2) in-house *C. purpurascens* database (40 entries). Search parameters included the following: nonenzymatic mass error of 10 ppm for precursor peptides and 0.02 Da for fragment ions. Fixed modification, carbamidomethyl (C), was introduced and several previously reported conopeptide PTMs were introduced as variable modifications: oxidation (M/P), carboxylation (E), bromination (W), deamidation (N/Q), pyroglutamate (N-terminus), and amidation (C-terminus). The false discovery rate (FDR) threshold was set to 1% using a decoy database. Only high and medium confidence protein matches were considered for downstream analysis.

### Experimental Design and Statistical Rationale

The sample size (n = 27) was the total number of specimens we had available for the intraspecies analysis. This large sample size provides a comprehensive view of *C. purpurascens* venom and maximizes peptide IDs. A comparison of venom profiles was performed with hierarchal cluster analysis and principal component analysis.

### Hierarchal Cluster Analysis and Principal Component Analysis

Total intensities were normalized to the conopeptide with the highest intensity within each sample. Total intensities were normalized in this analysis to account for differences in protein concentration between venom samples. Hierarchal clustering and principal component analysis were performed using ClustVis software (v. 2018-12-20) (42). Normalized intensities were log-transformed (ln(x + 1)) prior to hierarchal cluster analysis. Hierarchal clustering was employed on both *x*- and *y*-axes using Pearson correlation distance with average linkage.

## Results

### Conopeptide Identification

The venom analysis from 27 specimens of *C. purpurascens* yielded 543 unique conopeptide identifications, which included 33 base conopeptides and their toxiforms. Of these, 21 base conopeptide sequences were identified for the first time (Table 1). Detailed descriptions of each new conopeptide are provided in supplemental data. Twenty-six of the conopeptides were identified through the transcriptome-assembled search database. However, seven conopeptides were identified in the venom but were not found in either of the transcriptomes. Four of these peptides were identified from *C. purpurascens* UniProt entries (α-PIA, α-PIB, κ-PIVF, PVIF), and three conopeptides were sequenced *de novo* and added to our in-house search database (Contryphan-P4, PIF, and PIG). For the conopeptides that were identified from RNAseq data, full or partial transcripts were used to assign superfamilies through their corresponding signal sequence (supplemental Table S3).

**Table 1:** Conopeptides identified from the injected venom of *Conus purpurascens*

Superfamily	Conopeptide	Sequence	Toxiforms
A	α-PIA	RDPCCSNPVCTVHNPQIC	18
A	α-PIB	QSPGCCWNPAC-VKNR—C	6
A	α-PIC	TSGCCKHPAC-GKNR—C	1
A	**PID**	DPCCSNPACNVNNPQICG	11
A	**PIE**	NAAAKAFDLTAPTAGEGCCFNPACAVNNPNIC	2
A	**PIF∗**	QEPGCCRNPAC-VKHR—C	13
A	**PIG∗**	PCCSNPVCTVHGGPQLC	2
A	αA-PIVA	GCCGSYPNAACHPCSCKDRPSYCGQ	98
A	κ-PIVE	DCCGVKLEM-CHPCLCDNSCKNYGK	69
A	κ-PIVF	DCCGVKLEM-CHPCLCDNSCKKSGK	32
A	**PIVH**	DCCGVVMEE-CHKCLCNQTCKKK	45
B2	**Linear-P**	QPSAENEEGKFRFFDKQ	6
M	**Ile-Contryphan-P**	GCVIWPWC	7
M	**Contryphan-P3**	CAIWTKC	3
ND	**Contryphan-P4∗**	CVYWRKC	1
M	ψ-PIIIE	HPPCCLYGKCRRYPGCSSASCCQR	27
M	**PIIIG**	QWGCCPVNACRSCHCC	2
M	**PIIIH**	KCCPLTACKLGSGCKCCE	7
M	**PIIII**	CCQA-YCSRYHCLPCC	1
O1	δ-PVIA	EACYAPGTFCGIKPGLCCSEFCLPGVCFG	3
O1	**PVIB**	QCTPYGGSCGVD-STCCGRCNVPRNKCE	67
O1	**PVIC**	EACYAPGTFCGIKPGLCCSALCLPAVCID	--
O1	**PVID**∗∗	PCKKSGRKCFPHQKDCCGRACIITICP	3
O1	**PVIE**	VGEFRGCAHINQACNPP-QCCRGYTCQSSYIPSCQL	16
ND	**PVIF**∗∗	ATSNRPCKKTGRKCFPHQKDCCGRACIITICP	3
O1	**PVIG**∗∗	GATSNRPCKIPGRKCFPHQKDCCGRACIITICP	16
O1	κ-PVIIA	CRIPNQKCFQHLDDCCSRKCNRFNKCV	18
O2	Contryphan-P	GCPWDPWC	1
O3	**PIIA**	CCCIRSDGPKCSRKCLSSFFC	2
S	**PVIIIA**	GCSGSPCFKNKTCRDECICGGLSNCWCGYGGSRGCKCTCRE	33
T	PVA	GCCPKQMRCCTL	2
T	**PVB**	DCCPEKMWCCPL	11
Con-ikot-ikot	**p21b**	FELLPSQDRSCCIRKTLECLENYPGQESQRAHYCQQDATTNCPDTYDFGCCPGYATCMSINAQNNVRPAHDTCINRLCFDPGF	--

Conopeptides reported for the first time here are in bold. Conopeptides identified with the PEAKS *de novo* software are indicated by (∗). ∗∗These base conopeptides were previously described from cDNA libraries as P2B-D (58); since these designations do not conform with current conopeptide nomenclature, we have renamed them accordingly. The number of toxiforms only includes peptides identified with full cysteine framework.

Abbreviation: ND, not determined.

**Fig. 1:**
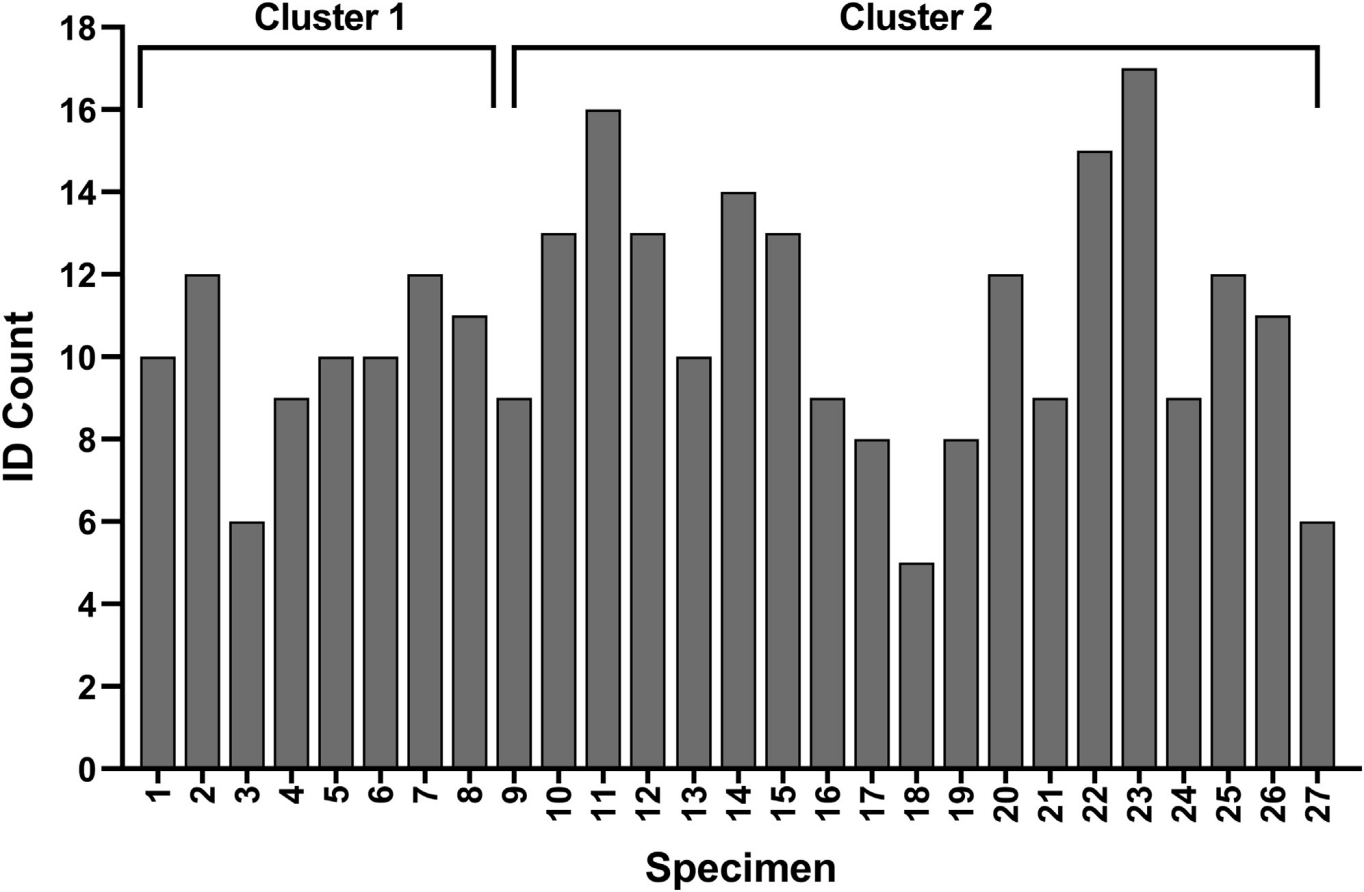
**Total conopeptide IDs for 27 *C. purpurascens* injected venom samples**.

**Fig. 2:**
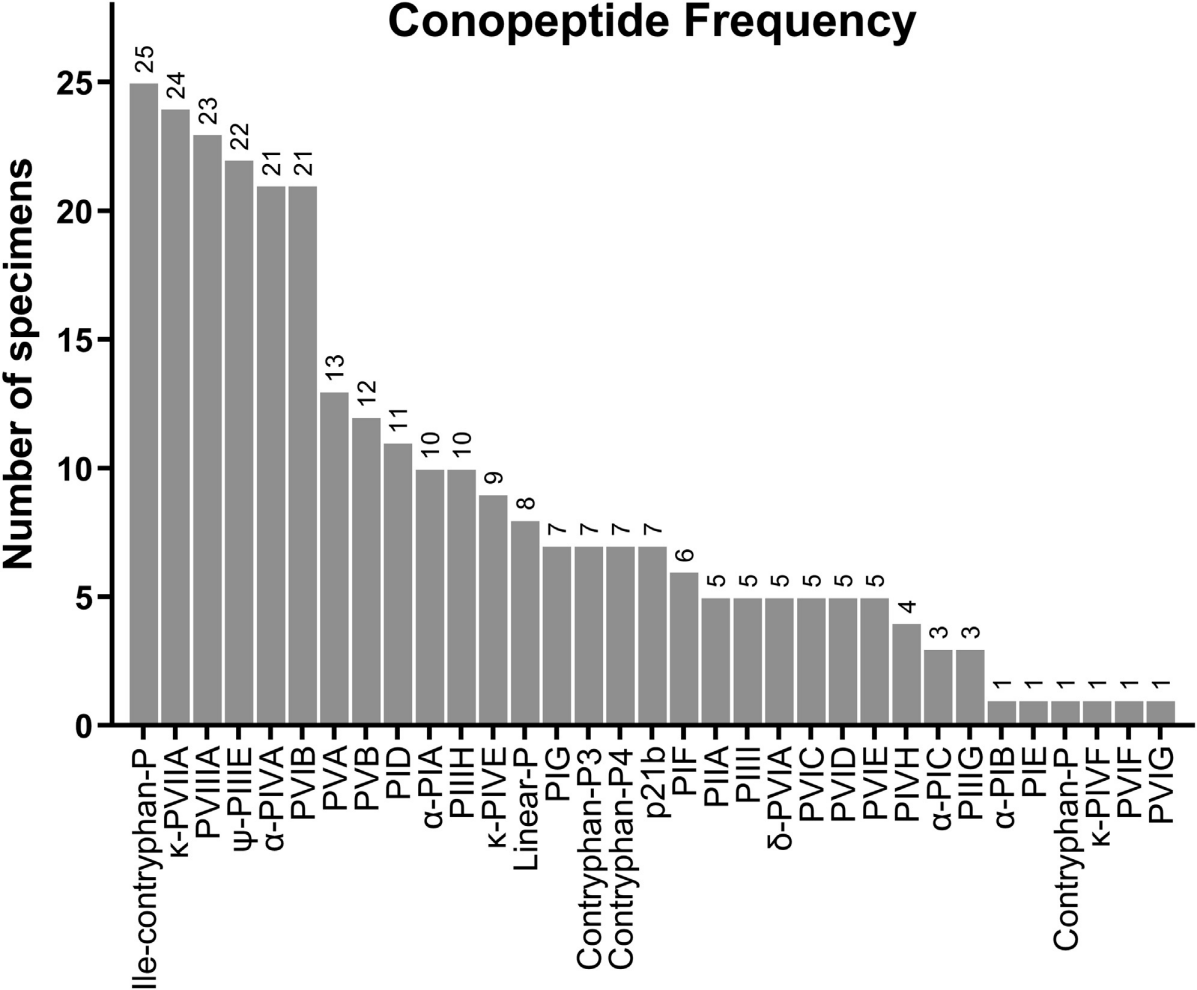
**Conopeptide frequency in injected venom from a population of *C. purpurascens* (n = 27)**.

The number of base conopeptide IDs per sample of injected venom ranged from 5 to 17 (mean= 10.6 ± 2.6) (Fig. 1). The most prevalent conopeptide in this population of snails was Ile-contryphan-P, identified in 25 of the 27 venom samples. This was followed closely by conopeptides κ-PVIIA, PVIIIA, ψ-PIIIE, α-PIVA, and PVIB, all identified in more than 75% (n > 20) of the venom samples (Fig. 2).

Toxiforms were assessed for each base conopeptide (supplemental Tables S4–S35), and the total numbers for each are reported in Table 1. Toxiforms were only considered if the peptide maintained a complete cysteine framework. The PTMs identified through MS/MS analysis included amidated C-terminal, hydroxyproline, oxidized methionine, deamidated asparagine/glutamine, carboxyglutamate, brominated tryptophan, N-terminal pyroglutamate, and truncations from both terminals. Modifications such as asparagine deamidation and methionin oxidation can be the result of sample processing; therefore, toxiforms identified with these modifications would need futher validation on whether they are naturally occurring. The most abundant PTMs were C-terminal amidation and hydroxyproline, occurring on 25 and 24 base conopeptides respectively. Differentially modified toxiforms were identified for 27 of the 33 base conopeptides (Table 1). The same modification(s) occurred on different residues of the same peptide, generating unique toxiforms with the same molecular weight. This is the case of hydroxyproline, which occurred on up to three residues simultaneously on four peptides: α-PIVA, ψ-PIIIE, PVIE, and PVIG. Differential hydroxylation patterns are seen for these conopeptides (supplemental Tables S4–S35). The greatest PTM variability was observed on A-superfamily conotoxins α-PIVA (104 toxiforms) and κ-PIVE (69 toxiforms) and new O1-superfamily conopeptide PVIB (69 toxiforms).

### Intraspecific Venom Comparison

**Fig. 3:**
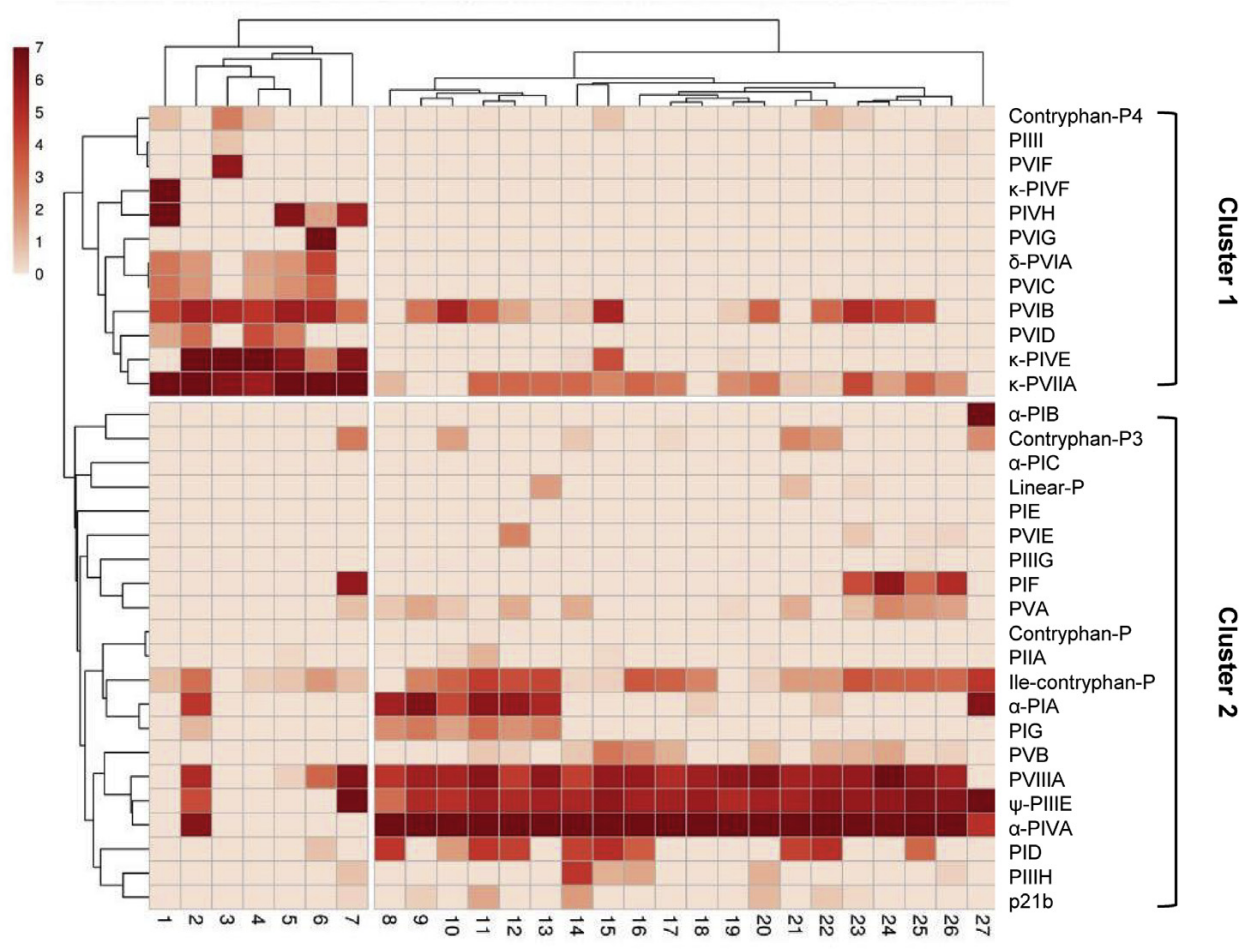
**Normalized intensity (MS/MS) of conopeptides in the injected venom of 27 individuals.** Ion intensities were normalized to the highest value for each specimen and ln(x + 1)-transformed. Both rows and columns are clustered using correlation distance and average linkage.

**Fig. 4:**
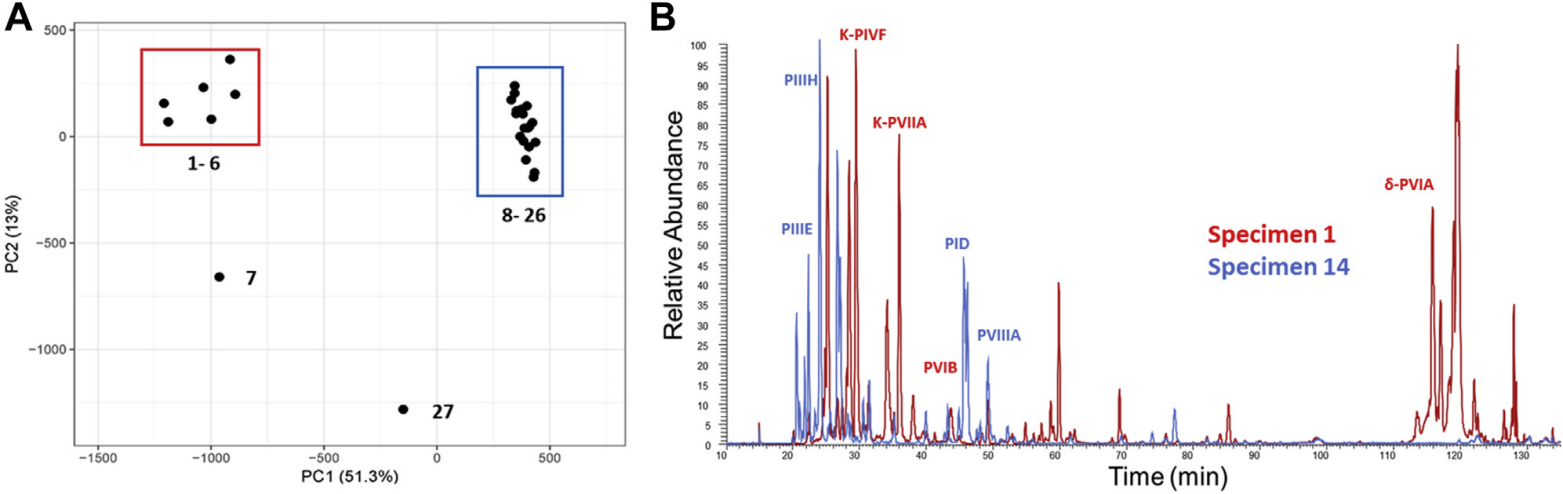
**Analysis of the conopeptides of the injected venom of*****C. purpurascens.**A*, PCA analysis of normalized ion intensity for all conopeptide IDs (n = 27). *B*, total ion chromatogram (TIC) overlay of Specimen 14 from cluster 1 (*blue*) and specimen 1 from cluster 2 (*red*).

Conopeptide expression profiles varied among the 27 samples. Two groups were distinguished from cluster analysis of the 33 base conopeptides, specimens 1–7 and 8–27 (Fig. 3). Clustering along the y-axis distinguished two groups of conopeptides that correlate to different venom compositions. The first cluster (snails 1–7) is mainly comprised of δ- and κ-conotoxins that target the sodium and potassium channels, respectively. These conotoxins make up the “lightning strike” cabal that rapidly immobilizes prey by acting on ion channels. The second cluster (snails 8–27) contains ψ- and α-conotoxins that both act on nAChRs and make up the “motor cabal.” Principal component analysis supported this dual expression pattern in the venom and clustered samples into two distinct groups of specimens 1–6 and specimens 8–26, with specimens 7 and 27 as outliers (Fig. 4*A*). An overlay of total ion chromatograms (TIC) from specimen 5 from cluster 1 (blue) with specimen 14 from cluster 2 (red) emphasizes the distinction in venom profile components between the two clusters (Fig. 4*B*).

The conopeptide identifications were made from venom gland transcriptome databases of two *C. purpurascens* specimens (transcriptomes A and B). These specimens correspond to specimen venom samples 1 (snail sacrificed for transcriptome A) and 14 (snail sacrificed for transcriptome B). To assess the coverage of the milked venom sample by the corresponding transcriptome, we compared conopeptide expression between these two specimens (supplemental Table S4). Our comparison examines the expression of each peptide between the two specimens at both transcriptomic (TPM) and proteomic (relative intensity) levels. Conopeptides expressed in the venom gland but not identified in the injected venom sample are shown in gray. Conopeptides identified in injected venom sample, but not expressed in venom gland transcriptome, are shown in blue. Overall, we see a differential expression pattern between the two specimens and between transcriptomic and proteomic expressions within the same specimen.

### Novel S-Superfamily Conotoxin PVIIIA

**Fig. 5:**
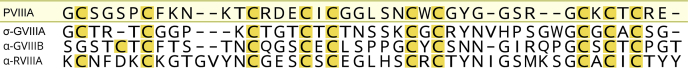
**Alignment of PVIIIA with characterized S-superfamily conotoxins σ-GVIIIA, α-RVIIIA, and α-GVIIIB**.

**Fig. 6:**
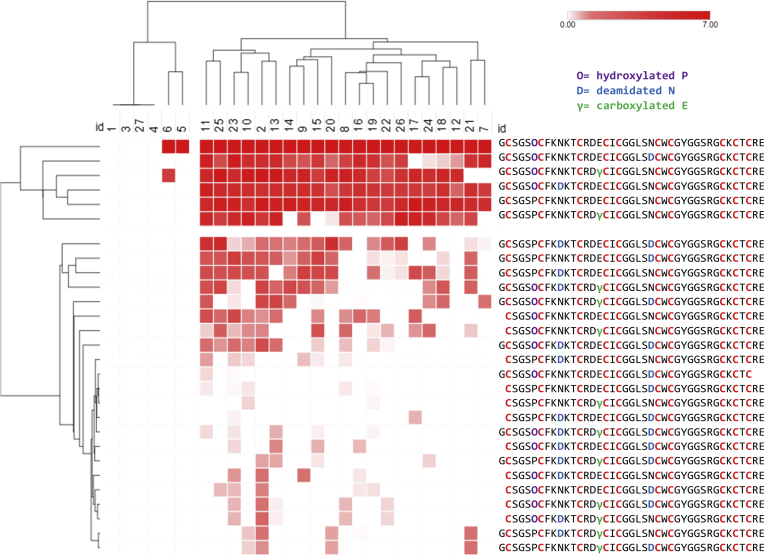
**PVIIIA toxiforms across 27 *C. purpurascens* specimens.** Total MS/MS intensities were calculated for each toxiform. Ion intensities were normalized to the highest value for each specimen and ln(x + 1)-transformed. Rows and columns are clustered using correlation distance and average linkage.

The new conotoxin, PVIIIA, is the first member of the S-superfamily found in injected venom. The peptide has five disulfides and exhibits cystine framework VIII (C-C-C-CX_aa_C-CX_aa_C-CX_aa_CX_aa_C). It was expressed in high frequency and abundance within this *C. purpurascens* population. It was identified in 23 of the 27 venom samples (Fig. 2). When venom profiles were compared, PVIIIA expression clustered closely with α-PIVA and ψ-PIIIE, which both target nicotinic receptors as part of the motor cabal (Fig. 3). Alignment with functionally characterized S-superfamily conotoxins, known to target serotonin (σ-GVIIIA) and nicotinic (α-GVIIIB, α-RVIIIA) receptors, exhibits very little sequence homology aside from the conserved cysteine framework (Fig. 5). PVIIIA is 41 residues in length and has five sites of modification, as determined by MS/MS spectral matching. We mapped all identified PTMs for the 33 toxiforms of PVIIIA (supplemental Table S32). The following sites of modification were determined: hydroxylated Pro(6), carboxylated Glu(16), deamidated Asn(10, 24), and truncations on both N- and C- terminals. These modifications occur in most possible combinations, significantly expanding the molecular diversity of the PVIIIA base peptide. We also compared toxiform expression among the 27 specimen samples (Fig. 6). The heatmap shows two clusters of peptides, which correlate to high abundance (top cluster) and lower abundance (bottom cluster). The six toxiforms in the top cluster exhibit the highest expression within the samples, as shown by color, and also within the population.

## Discussion

Proteogenomic approaches, including proteotranscriptomics, are ideally suited to study venom. The proteinaceous nature of venom allows a comprehensive assessment of the venom composition (venome) and the study of venom dynamics (venomics). Here we have applied venomics to study the intraspecific variability of the injected predatory venom used by *C. purpurascens*, a fish-hunting cone snail that has been studied intensively for the past 25 years (43, 44). Studies on cone snail venom are quite significant, as the venom is a valuable source of bioactive peptides that can be used as neuronal probes and developed as novel therapeutic agents. Several conopeptides have reached clinical trials, including the approval of Prialt, among the most powerful painkillers known (45). Analysis of the intrinsic complexity of cone snail venom has been significantly advanced with the advent of NGS transcriptomic data that provides thousands of novel putative conopeptide sequences—a trend that will continue to expand. It is critical to probe venom using proteomic approaches, as transcriptomic data on its own can only provide putative sequences. Large-scale top-down proteomics/peptidomics is the best way to assess *de facto* PTMs and cleavage sites to generate mature conopeptides. We sought to maximize venom coverage through conopeptide identifications; however, practical aspects of these workflows, such as the number of available transcriptomes, size of the conopeptides suitable for “top down”/enzyme-free methods, and unforeseen PTMs, may have an effect on the final coverage of components obtained. While recognizing these limitations for complete venom coverage, we were able to increase component identification by including sequences discovered through *de novo* methods and sequences previously reported for *C. purpurascens* to our search database. Regardless of total coverage obtained, our results reveal a clear picture of the venom profiles and envenomation strategies employed by *C. purpurascens*.

We show through a functional proteogenomic comparison between specimens 1 and 14 that transcriptomic data from the venom gland does not provide complete coverage of the venom components. We identified conopeptides in the injected venom that were not represented at the transcript level, demonstrating the lack of homogeneity between the venom gland transcriptome and the injected venom. Of the 17 conopeptides reported in UniProt for *C. purpurascens*, seven were not found in either venom gland transcriptome (α-PIA, α-PIB, μ-PIIIA, ψ-PIIIF, κ-PIVF, p21a, conantokin-P, and Leu-contryphan-P). By combining transcriptomes from two specimens into a search database, we were able to increase our total proteome coverage of the venom. However, these results emphasize that a transcriptome is a snapshot of gene expression at the precise moment the animal was sacrificed for mRNA extraction and cannot be used alone to fully describe the dynamics of venom expression. Other limitations include missing toxin transcripts during the transcriptome assembly process, as *de novo* assemblers can face difficulties when attempting to process large numbers of closely related transcript isoforms (46).

Our aim was to achieve high-confidence peptide identifications to help describe the molecular mechanisms of predation utilized by this population of *C. purpurascens*, where all specimens were collected at the same geographical location, with similar sizes, and kept under controlled conditions in laboratory aquaria. Our venomics approaches led to the identification of 543 conopeptides, which are the result of 33 base sequences and their corresponding toxiforms, significantly expanding the current inventory of *C. purpurascens* conopeptides. As expected, these are only a fraction of the putative conopeptide base sequences predicted by transcriptomic expression or by the number of unique masses deconvoluted at the MS1 level (9). We were able to ascertain numerous toxiforms from the 33 identified base conopeptide sequences. Cone snails have the remarkable ability to engineer their venom peptides through hypermodification, a molecular adaptation to expand the reach of their venom (23, 25, 26). These PTMs may have important implications for development and molecular engineering of novel peptide-based therapeutics (47, 48, 49). Using spectral matching, we were able to detect sites of differential hydroxylation and carboxylation, which could not be deciphered through mass matching alone.

Our results emphasize the importance of identifying venom components from the injected venom, the actual brew delivered into prey. This is in striking contrast to intraspecific studies that utilized dissected venom (5, 36), which neglect venom processing and delivery at several levels. This is the first study using high-resolution MS, transcriptomic data, and *de novo* approaches on the injected venom of a large group of individuals of the same species for global identification of components, assessment of venom dynamics, and evaluation of synergistic interactions between conopeptides and their potential pharmacology.

The conopeptide composition of the predatory injected venom arsenal of *C. purpurascens* consists of cysteine-constrained peptides that range from one disulfide bond (contryphans) to five disulfide bonds (PVIIIA and p21b). The outlier is the linear peptide (Linear-P) belonging to the B2-superfamily. The molecular masses ranged from 938 Da (Contryphan-P3) to 4960 Da (PVIIIA), indicating a widespread of molecular features of these venom components. These venomes are covered by conotoxin frameworks I–VIII, X, and 21. Except for frameworks II, VIII, and 21, 3D structural information exists to help assign disulfide bonding and folding patterns (50, 51, 52, 53) to these newly discovered *C. purpurascens* conotoxins. This is particularly true for the well-studied α-conotoxins (framework I) and κ-, δ-conotoxins (frameworks VI, VII). Structural assignments of the more complex frameworks, such as those found in PVIIIA and p21b (5 disulfide bonds), remain a challenge. While the structural and even functional features of novel base sequences can be predicted by homology, such as PID, PIE, PIF (which are homologues of other well-characterized α-conotoxins), others such as PIIA, PIIIG-I, PVIE, and PVIIIA have no significant homology to functionally characterized conotoxins; and therefore their activity and role in the envenomation strategy will require further investigation.

Hierarchical cluster analysis of the venom profiles of 27 specimens enabled us to ascertain strong linkages and possible synergisms between specific conopeptides through coexpression patterns. We found two distinct clustering patterns indicating that two different venom cabal combinations can be employed by *C. purpurascens* as a hunting strategy. Cluster 1 contains classical members of the lightning strike cabal, affecting neuronal transmission by disrupting the propagation of action potentials (δ-PVIA, κ-PVIIA, κ-PIVE), but not apparent members of the motor cabal, comprising paralytic toxins acting primarily on nicotinic muscular targets (α or αA conotoxins). These findings provide a significant revision to the original venom cabal configurations for *C. purpurascens*. The original cabal concept was introduced by the synergy of conotoxins κ-PVIIA and δ-PVIA (the lockjaw peptide) found in the pooled venom from several individuals of *C. purpurascens* collected in the Gulf of California (33, 43). However, when using pooled venom, the lightning strike cabal would be complemented with members of the motor cabal that includes several inhibitors of nAChRs such as a αA (PIVE, PIVF, PIVG) and ψM (PIII-I) conotoxins, which is not the case for individuals within cluster 1 (nonpooled samples). Since conotoxins PIVE, κ-PVIIA, δ-PVIA, and their respective toxiforms, and novel conotoxins, PIVH, PVIB, PVIC, PVID (and toxiforms), are part of cluster 1, the latter appear to complement the lighting strike cabal within those *C. purpurascens* individuals.

Cluster 2 contains several inhibitors of nAChRs such as α-PIA-F, αA-PIVA, and ψ-PIIIE conotoxins in addition to components of the lighting strike cabal, δ-PVIB and κ-PVIIA (also present in cluster 1). This is an indication of the use of multiple cabals as the primary arsenal of this populations of *C. purpurascens*. The role of PVIIIA is intriguing, as it is highly expressed in cluster 2, but given the abundance of nicotinic inhibitors already present there, it would be unlikely that another more complex nicotinic inhibitor is necessary to complete the motor cabal. Another curious finding within cluster 1 was the presence of mini-M conotoxins PIIIG, PIIIH, and PIII-I. While these conotoxins are prevalent in worm and mollusk-hunting *Conus* species (54, 55), they have not been found in the injected venom of fish hunting species until now. The significance of this finding is under investigation.

We use PVIIIA as an example to demonstrate toxiform variability among the 27 venom samples. We found that the toxiforms are differentially expressed throughout our *C. purpurascens* population, in a similar manner to that of the base peptides. A heatmap of PVIIIA toxiforms shows two clusters of peptides (Fig. 6). The top cluster is comprised of six toxiforms, which are expressed in high abundance, while the bottom cluster is expressed in lower abundance (bottom cluster). While the toxiform comparison does not provide an insight to its role in the venom, it can help distinguish which toxiforms are most abundant within the population and provide leads for downstream bioactivity assays.

Populations of cone snails in different habitats and geographical locations can show different venom phenotypes, as seen in *C. purpurascens* venom studies carried out on animals from the Gulf of California (33), The Clipperton atoll (56), Ecuador (57), Panama (3), and Costa Rica (10) showing profound differences in venom profiles. For example, p21a, a conotoxin with the putative ability to modulate AMPA receptors, was found in a *C. purpurascens* specimen from Ecuador (57), but not in the animals from Costa Rica here studied. However, the homologous conotoxin p21b was found as part of cluster 2, but not cluster 1. Given the differences in cabals between clusters 1 and 2, it is likely that p21b participates in the lightning strike cabal within cluster 2 in lieu of PVIA, which is absent in this cluster. Habitat is critical to these slow-moving creatures as they must adapt to very localized areas. Part of this adaptation process will be venom production to capture prey that are prevalent to these microhabitats. Accordingly, venom profiles that we found might be a product of such adaptation. This adaption appears to be imprinted over their development in the wild, as upon captivity the venom remains invariant as these animals were fed and kept under identical conditions.

Despite extensive studies on *C. purpurascens* through decades, using HR-MS/MS spectral matching, we have revealed a deeper coverage of the components of the injected venom from 27 specimens of *C. purpurascens*. Furthermore, we have shown the dramatic venom variations from specimen to specimen and the dynamic interaction of components as revealed by two patterns of synergism. These findings further develop the cabal concept in several ways. (1) The expanded reach of components due to the hypermodification to generate a plethora of toxiforms, (2) novel components belonging to distinct cabals, and (3) the possibility of multiple cabals operating independently within the same geographical group of individuals. In addition to providing the strongest evidence of venom cabals, to date, these findings will allow us to predict molecular targets of uncharacterized conopeptides based on global expression patterns. These analyses can aid the convoluted process of developing conotoxins/conopeptides into valuable molecular probes or therapeutics (supplemental Figs. 3–26 and Tables 36–39).

## Data availability

All identified conopeptide sequences from this study are available on UniProt. Mass spectra RAW files and search result files are available on MassIVE with the dataset identifier MSV000085498 (ftp://massive.ucsd.edu/MSV000085498/).

## Supplemental data

This article contains supplemental data.

## Conflict of interest

The authors declare no competing interests.
