# Supplementary material for: Proteogenomic Assessment of Intraspecific Venom Variability: Molecular Adaptations in the Venom Arsenal of Conus purpurascens
Source: Mol Cell Proteomics. 2021 May 23;20:100100. doi: 10.1016/j.mcpro.2021.100100 (PMC8260871; doi:10.1016/j.mcpro.2021.100100)
Supplement: Supplemental Figures S1–S26 and Tables S1–S39 [file mmc1.docx]

**Proteogenomic Assessment of Molecular Adaptations in the Venom Arsenal of *Conus purpurascens***

Meghan Grandal^1,2^, Mickelene Hoggard^1^, Benjamin Neely^1^, W. Clay Davis^1^ and Frank Marí^1*^

^1^Chemical Sciences Division, National Institute of Standards and Technology, Hollings Marine Laboratory, 331 Fort Johnson Road, Charleston, SC, 29412, USA.

^2^Department of Drug Discovery, Medical University of South Carolina, 70 President Street, Charleston, SC 29425, USA.

**Supplementary Information**

| File ID | Specimen ID | Size (mm) |
| --- | --- | --- |
| 3-L | 1 | 62 |
| 5-3M | 2 | 51 |
| 3-M | 3 | 50 |
| 7-S | 4 | 45 |
| 8-2M | 5 | 41 |
| 6-3L | 6 | 57 |
| 6-M | 7 | 52 |
| 3-6M | 8 | 46 |
| 6-2L | 9 | 53 |
| 7-2S | 10 | 46 |
| 3-2L | 11 | 45 |
| 8-L | 12 | 47 |
| 8-3S | 13 | 32 |
| 5-2Mb | 14 | 53 |
| 5-5M | 15 | 43 |
| 3-2M | 16 | 55 |
| 6-2M | 17 | 43 |
| 7-3S | 18 | 43 |
| 5-4M | 19 | 53 |
| 8-2S | 20 | 37 |
| 6-4L | 21 | 60 |
| 8-M | 22 | 42 |
| 5-M | 23 | 54 |
| 7-M | 24 | 52 |
| 8-4M | 25 | 44 |
| 8-S | 26 | 39 |
| 7-L | 27 | 55 |

**Table S1-** Information on the 27 *C. purpurascens* specimen samples used in this analysis.

| **Sequence File** | **Description** | **# Sequences** |
| --- | --- | --- |
| 1. Transcriptome A | *De novo* assembly of RNA Seq data from snail A | 83,051 |
| 2. Transcriptome B | *De novo* assembly of RNA Seq data from snail B | 84,410 |
| 3. Translated– getorf | Files 1 and 2 translated with getorf | 231,175 |
| 4. Translated– transeq | Files 1 and 2 translated with transeq | 1,004,766 |
| 5. ToxProt BLAST hits | Files 3 and 4 searched against ToxProt database (blastp) | 1,652 |
| 6. *C. purpurascens*  database | All known *C. purpurascens* conopeptides including UniProt entries (taxid: 41690) and *de novo* sequences | 40 |

**Table S2-** FASTA sequence file descriptions. Files 5 and 6 (gray) were used as search databases

| **Superfamily** | **Conopeptide** | **Transcript** |
| --- | --- | --- |
| A | PIC | MGMRMMFIVFLLVVLATTVGSFTLDRVLGLASEGRNAEAIDNALDQRDPKRR**TSGCCKHPACGKNRC** |
|  | PID | MGMRMMFIVFLLVVLATTVVSFTLDRASDGRDAAANDKASDLIALTARR**DPCCSNPACNVNNPQICG** |
|  | PIVA | MGMRMMFIVFLLVVLATTVVSFTSDRASDDRNTNDKASRLLSHVVR**GCCGSYPNAACHPCSCKDRPSYCGQGR** |
|  |  | MGMRMMFIVFLLVVLATTVGSFTSDRASDDRNTNDKASRLLSHVVR**GCCGSYPNAACHPCSCKDRPSYCGQGR** |
|  | PIVE | MGMRMMFIVFLLVALATTVGSFTSDRASDGRNAAVNDKASHLIDNVIR**DCCGVKLEMCHPCLCDNSCKNYGK**GKKEYGK |
|  | PIVH | VVLATTVVSFTSDRASDGRNAAVNDKASPLIAKVIR**DCCGVVMEECHKCLCNQTCKKK**GKKELWEMMTATDKRNT |
| B2 | B2 linear | MLRLIIAAVLASACLAFPERRDGVPAEQANLQGFDPAAQAMPAMAGMQQMPGMAGGQFLPFNPNFGMAYKRDMDESLEKRKQHSQFNADNESPFEAGDNLGDFMNFMKGNGNNVPFANMDSDATDLGNF**QPSAENEEGKFRFFDKQ**Q |
|  |  | MLRLIIAAVLASACLAFPERRDGVPAEQANLQGFDPAAQAMPAMAGMQQMPGMAGGQFLPFNPNFGMAYKRDMDEILEKRKQHSQFNADNESPFEAGDNLGDFMNFMKGNGNNVPFANMDSDATDLGNF**QPSAENEEGKFRFFDKQ**Q |
| M | Ile-Contryphan-P | MLKMGVLLFTFLVLFPLATLQLDADQPVERYVEKKQDLNPDERTKTLHALRPPSVDKRATSL**GCVIWPWC** |
|  | Contryphan P3 | MLKMGVLLFIFLVLLPLATLQLDADQPVERYAENKQDLKPDERREIILPALGPPSVDKRATSLA**CAIWTKC** |
|  | PIIIE | MMSKLGALLTICLLLFPITALLMDGDQPADRPAERMEDDISSEVHRLLERR**HPPCCLYGKCRRYPGCSSASCCQR**G |
|  |  | MMSKLGALLTICLLLFPITALLMDGDQPADRPAERMEDDISSEVHRLLERR**HPPCCLYGKCRRYPGCSSASCCQR**G |
|  | PIIIG | LITLQLDADQPVERYAEDKQDLNPNERMGFILPALR**QWGCCPVNACRSCHCC**GRSTSVALCWADSTATAVVDHVYYRAHVSCLRMTN |
|  | PIIIH | MLKMGVLLFTFLVLFPLATLQLDADQPVERYAENKQDLKPDERREIILPALGQR**KCCPLTACKLGSGCKCCE** |
|  | PIII-I | MMFKLGVLLTICLLLVPLTAIPLDGDQPVDQPAERMEDGKSTPNHPWFDPVKR**CCQAYCSRYHCLPCC** |
| O1 | PVIA | MKLTCVMIVAVLFLTAWTFVTADDSKNGLENHFWKARDEMKNREASKLDKK**EACYAPGTFCGIKPGLCCSEFCLPGVCFGG** |
|  |  | MKLTCVMIVAVLFLTAWTFVTADDSKNGLENHFWKARDEMKNREASKLDKK**EACYAPGTFCGIKPGLCCSEFCLPGVCFGG** |
|  | PVIB | MKLTCVVIVAVLFLTACQLITADDSRRTQKHRALRSTTKLSMLTR**QCTPYGGSCGVDSTCCGRCNVPRNKCE** |
|  | PVIC | MKLTCVMIVAVLFLTAWTFVTADDSKNGLENHFWKARDEMKNREASKLDKK**EACYAPGTFCGIKPGLCCSALCLPAVCID**G |
|  | PVID | MKLTCVVIVAVLFLTACQLITADDSRRTQKHRALRSTTKRARSNR**PCKKSGRKCFPHQKDCCGRACIITICP** |
|  | PVIE | MKLTCVLIIAVLFLTACQLITAGYSRDKQVYRAVRLGDKMLR**VGEFRGCAHINQACNPPQCCRGYTCQSSYIPSCQL** |
|  | PVIG | MKLTCVVIVAVLFLTACQLITADDSRRTQKHRALRSTTK**GATSNRPCKIPGRKCFPHQKDCCGRACIITICP** |
|  | PVIIA | MKLTCVVIVAVLFLTACQLITADDSRRTQKHRALRSTTKLSLSTR**CRIPNQKCFQHLDDCCSRKCNRFNKCV** |
|  |  | MKLTCVVIVAVLFLTACQLITADDSRRTQKHRALRSTTKLSLSTR**CRIPNQKCFQHLDDCCSRKCNRFNKCV** |
| O2 | Contryphan-P | MGKLTILLLVAAVLLSTQVMVQGDGDQPAYRNAAPRDDNPGGAIGKFMNVLRRS**GCPWDPWC**G |
| O3 | PIIA | MSRFGIMVLTFLLLVSMATSHRYARGKQATRRNAINIRRRSTPKTEACEEVCELEEKH**CCCIRSDGPKCSRKCLSSFFC** |
| S | PVIIIA | MMSKMGAMFVLLLLFTLASSQQEGDVQARKTRLTRDFYRTLPVSTR**GCSGSPCFKNKTCRDECICGGLSNCWCGYGGSRGCKCTCRE** |
|  |  | MMSKMGAMFVLLLLFTLASSQQEGDVQARKTRLTRDFYRTLPVSTR**GCSGSPCFKNKTCRDECICGGLSNCWCGYGGSRGCKCTCRE** |
| T | PVA | MRCLPVFVILLLLIASAPSVDAHPKTKDDMPLASFHDNAKGTLQRLWKKR**GCCPKQMRCCTL**G |
|  | PVB | MHCLPVFVILLLLIPSAPCVDAHPKTKDDMPLASFHDNAKRTLQRFWKKR**DCCPEKMWCCPL**G |
|  |  | MRCLPVFVILLLLIASAPSVDAHPKTKDDMPLASFHDNAKRTLQRFWKKR**DCCPEKMWCCPL**G |
| Con-ikot-ikot | p21b | MNMSMTLSMFVMVVVAATVTG**FELLPSQDRSCCIRKTLECLENYPGQESQRAHYCQQDATTNCPDTYDFGCCPGYATCMSINAQNNVRPAHDTCINRLCFDPGF** |

**Table S3-** Conopeptide transcripts from transcriptome A (unshaded) and B (shaded)

|  | | Specimen 1 | | Specimen 14 | |
| --- | --- | --- | --- | --- | --- |
| Superfamily | Conopeptide | Transcriptome A (TPM) | MS Relative Intensity | Transcriptome B (TPM) | MS Relative Intensity |
| A | α-PIA | 0.00 | 0.00 | 0.00 | 0.00 |
| A | α-PIB | 0.00 | 0.00 | 0.00 | 0.00 |
| A | α-PIC | 6657.49 | 0.00 | 1214.88 | 0.00 |
| A | PID | 139.43 | 0.00 | 0.00 | 67.16 |
| A | PIE | 25.81 | 0.00 | 50.15 | 0.00 |
| A | PIF | 0.00 | 0.00 | 0.00 | 0.00 |
| A | PIG | 0.00 | 0.00 | 0.00 | 0.00 |
| A | α-PIVA | 8002.60 | 0.00 | 7115.58 | 1000.00 |
| A | κ-PIVE | 0.00 | 0.00 | 1043.68 | 0.22 |
| A | κ-PIVF | 0.00 | 989.76 | 0.00 | 0.00 |
| A | PIVH | 3962.08 | 1000.00 | 0.00 | 0.00 |
| B2 | Linear-P | 7081.46 | 0.00 | 2802.38 | 0.00 |
| M | Ile-Contryphan-P | 1985.81 | 1.23 | 0.00 | 0.38 |
| M | Contryphan-P3 | 0.00 | 0.00 | 512.22 | 0.87 |
| ND | Contryphan-P4 | 0.00 | 1.29 | 0.00 | 0.00 |
| M | ψ-PIIIE | 1926.10 | 0.00 | 770.99 | 186.94 |
| M | PIIIG | 6.84 | 0.00 | 0.00 | 0.00 |
| M | PIIIH | 0.00 | 0.00 | 2988.25 | 97.70 |
| M | PIIII | 0.00 | 0.02 | 8.32 | 0.00 |
| O1 | δ-PVIA | 2642.86 | 13.74 | 3651.60 | 0.00 |
| O1 | PVIB | 0.00 | 58.13 | 257.39 | 0.75 |
| O1 | PVIC | 632.78 | 14.82 | 0.00 | 0.00 |
| O1 | PVID | 363.92 | 3.02 | 0.00 | 0.00 |
| O1 | PVIE | 0.00 | 0.00 | 152.04 | 0.01 |
| ND | PVIF | 0.00 | 0.00 | 0.00 | 0.00 |
| O1 | PVIG | 0.00 | 0.00 | 569.10 | 0.00 |
| O1 | κ-PVIIA | 934.23 | 935.37 | 1387.09 | 21.09 |
| O2 | Contryphan-P | 57382.30 | 0.00 | 4647.95 | 0.00 |
| O3 | PIIA | 22.33 | 0.00 | 0.00 | 0.00 |
| S | PVIIIA | 4082.19 | 0.00 | 1193.34 | 74.22 |
| T | PVA | 340.10 | 0.00 | 0.00 | 2.68 |
| T | PVB | 73.58 | 0.00 | 403.84 | 0.83 |
| Con-ikot-ikot | P21b | 0.00 | 0.00 | 71.93 | 4.46 |

**Table S4-** Comparison of transcriptome expression (TPM) and MS identification from venom sample (relative intensity) from the two specimen that were dissected for RNAseq analysis of the venom duct (Specimen 1-Transcritpme A, Specimen 14- Transcriptome B). Differential expression patterns of expression are shown by shading. Conopeptides expressed in the venom duct but not identified in the injected venom sample are shown in grey. Conopeptides identified in injected venom sample, but not expressed in venom duct transcriptome are shown in blue. ND = not determined

| α-PIA | R | R | D | P | C | C | S | N | P | V | C | T | V | H | N | P | Q | I | C | G |
| --- | --- | --- | --- | --- | --- | --- | --- | --- | --- | --- | --- | --- | --- | --- | --- | --- | --- | --- | --- | --- |
|  |  |  |  | **O** |  |  |  |  | **O** |  |  |  |  |  | **D** | **O** | **E** |  | * | * |

| Annotated Sequence | MH+ [Da] |
| --- | --- |
| **CC**SNPV**C**TVHNPQI**C*** | 1844.77 |
| P**CC**SNPV**C**TVHNPQI**C*** | 1941.82 |
| P**CC**SNPV**C**TVHNPQI**C**G* | 1998.84 |
| DP**CC**SNPV**C**TVHNPQI**C*** | 2056.84 |
| DP**CC**SNPV**C**TVHNPQI**C** | 2057.83 |
| DP**CC**SNPV**C**TVHN**O**QI**C*** | 2072.84^+^ |
| DP**CC**SN**O**V**C**TVHNPQI**C*** | 2072.84^+^ |
| DP**CC**SNPV**C**TVHNPQI**C**G* | 2113.87 |
| RDP**CC**SNPV**C**TVHNPQI**C*** | 2212.95 |
| RDP**CC**SNPV**C**TVHNPQI**C** | 2213.93 |
| RDP**CC**SNPV**C**TVHN**O**QI**C*** | 2228.94^+^ |
| RDP**CC**SN**O**V**C**TVHNPQI**C*** | 2228.94^+^ |
| RDP**CC**SN**O**V**C**TVHNPQI**C** | 2229.92 |
| RDP**CC**SNPV**C**TVHN**O**QI**C** | 2229.93 |
| RDP**CC**SNPV**C**TVH**DO**QI**C*** | 2229.94^+^ |
| RDP**CC**SNPV**C**TVHN**OE**I**C*** | 2229.94^+^ |
| RDP**CC**SN**O**V**C**TVHN**O**QI**C*** | 2244.94^+^ |
| RD**OCC**SN**O**V**C**TVHNPQI**C*** | 2244.94^+^ |
| RDP**CC**SNPV**C**TVHNPQI**C**G* | 2269.97 |
| RDP**CC**SNPV**C**TVHNPQI**C**G | 2270.96 |
| RDP**CC**SN**O**V**C**TVHNPQI**C**G* | 2285.96 |
| RRDP**CC**SNPV**C**TVHNPQI**C*** | 2369.06 |

**Table S5-** α-PIA Toxiforms. **O** = hydroxyproline; * = amidated C-terminus; **Z** = pyroglutamate; **γ** = γ−carboxyglutamate; **m** = methionine sulfoxide; **D** = hydrolyze Asn, **E** = hydrolyzed Gln. ^+^ = positional assignment of the PTMs was confirmed manually. This convention for the PTMs applies to the rest of the tables.

| **α-PIB** | **Q** | **S** | **P** | **G** | **C** | **C** | **W** | **N** | **P** | **A** | **C** | **V** | **K** | **N** | **R** | **C** |
| --- | --- | --- | --- | --- | --- | --- | --- | --- | --- | --- | --- | --- | --- | --- | --- | --- |
|  | **Z** |  | **O** |  |  |  |  |  | **O** |  |  |  |  | **D** |  | ***** |

| Annotated Sequence | MH+ [Da] |
| --- | --- |
| G**CC**WNPA**C**VKNR**C*** | 1680.70 |
| **O**G**CC**WNPA**C**VKNR**C*** | 1793.74 |
| **Z**SPG**CC**WNPA**C**VKNR**C*** | 1975.82 |
| **Z**S**O**G**CC**WNPA**C**VKNR**C*** | 1991.81 |
| **Z**S**O**G**CC**WNPA**C**VK**D**R**C*** | 1992.79 |
| **Z**S**O**G**CC**WN**O**A**C**VKNR**C*** | 2007.80 |

**Table S6-** α-PIB Toxiforms

| **α-PIC** | **S** | **G** | **C** | **C** | **K** | **P** | **A** | **C** | **G** | **K** | **N** | **R** | **C** |
| --- | --- | --- | --- | --- | --- | --- | --- | --- | --- | --- | --- | --- | --- |

| Annotated Sequence | Thγo. MH+ [Da] |
| --- | --- |
| SG**CC**KHPA**C**GKNR**C** | 1691.70 |

**Table S7-** α-PIC Toxiforms

| **PID** | **R** | **D** | **P** | **C** | **C** | **S** | **N** | **P** | **A** | **C** | **N** | **V** | **N** | **N** | **P** | **Q** | **I** | **C** | **G** |
| --- | --- | --- | --- | --- | --- | --- | --- | --- | --- | --- | --- | --- | --- | --- | --- | --- | --- | --- | --- |
|  |  |  | **O** |  |  |  |  | **O** |  |  |  |  |  |  | **O** | **E** |  | ***** |  |

| **Annotated Sequence** | **MH+ [Da]** |
| --- | --- |
| **CC**SNPA**C**NVNNPQI**C*** | 1806.71 |
| P**CC**SNPA**C**NVNNPQI**C*** | 1903.77 |
| DP**CC**SNPA**C**NVNNPQI**C*** | 2018.79 |
| RDP**CC**SNPA**C**NVNNPQI**C*** | 2174.90 |
| RDP**CC**SNPA**C**NVNNPQI**C** | 2175.88 |
| RDP**CC**SNPA**C**NVNN**O**QI**C*** | 2190.89^+^ |
| RDP**CC**SN**O**A**C**NVNNPQI**C*** | 2190.89^+^ |
| RD**OCC**SN**O**A**C**NVNNPQI**C*** | 2206.88^+^ |
| RDP**CC**SN**O**A**C**NVNN**O**QI**C*** | 2206.89^+^ |
| RDP**CC**SNPA**C**NVNNPQI**C**G | 2232.90 |
| RDP**CC**SNPA**C**NVNN**OE**I**C**GRR**C**SRTLTKTy**CO**LPD | 4348.78^-^ |
| RDP**CC**SNPA**C**NVNNP**E**I**C**GRR**C**SRTLTKTy**CO**L**O**D | 4348.79^-^ |

**Table S8-** PID Toxiforms

| **PIE** | **N** | **A** | **A** | **A** | **K** | **A** | **F** | **D** | **L** | **T** | **A** | **P** | **T** | **A** | **G** | **E** | **G** | **C** | **C** | **F** | **N** | **P** | **A** | **C** | **A** | **N** | **N** | **P** | **N** | **I** | **C** |
| --- | --- | --- | --- | --- | --- | --- | --- | --- | --- | --- | --- | --- | --- | --- | --- | --- | --- | --- | --- | --- | --- | --- | --- | --- | --- | --- | --- | --- | --- | --- | --- |
|  |  |  |  |  |  |  |  |  |  |  |  | **O** |  |  |  |  |  |  |  |  |  | **O** |  |  |  |  |  |  |  |  |  |

| Annotated Sequence | MH+ [Da] |
| --- | --- |
| NAAAKAFDLTAPTAGEG**CC**FN**O**A**C**AVNNPNI**C** | 3412.48^-^ |
| NAAAKAFDLTA**O**TAGEG**CC**FNPA**C**AVNNPNI**C** | 3412.48^-^ |

**Table S9-** PIE Toxiforms

| PIF | Q | E | P | G | C | C | R | N | P | A | C | V | K | H | R | C |
| --- | --- | --- | --- | --- | --- | --- | --- | --- | --- | --- | --- | --- | --- | --- | --- | --- |
|  | **Z** | **γ** | **O** |  |  |  |  | **D** | **O** |  |  |  |  |  |  | ***** |

| Annotated Sequence | MH+ [Da] |
| --- | --- |
| G**CC**RNPA**C**VKHR**C*** | 1673.73 |
| PG**CC**RNPA**C**VKHR**C*** | 1770.79 |
| PG**CC**RN**O**A**C**VKHR**C*** | 1786.78 |
| **O**G**CC**RNPA**C**VKHR**C*** | 1786.79 |
| EPG**CC**RNPA**C**VKHR**C*** | 1899.83 |
| **Z**EPG**CC**RNPA**C**VKHR**C*** | 2010.86 |
| **Z**EPG**CC**R**D**PA**C**VKHR**C*** | 2011.84^+^ |
| **Z**EPG**CC**RNPA**C**VKHR**C** | 2011.85^+^ |
| **Z**EPG**CC**RN**O**A**C**VKHR**C*** | 2026.86^+^ |
| **Z**E**O**G**CC**RNPA**C**VKHR**C*** | 2026.86^+^ |
| **Z**E**O**G**CC**RN**O**A**C**VKHR**C*** | 2042.85 |
| **Zγ**PG**CC**RNPA**C**VKHR**C*** | 2054.85 |
| **ZγO**G**CC**RNPA**C**VKHR**C*** | 2070.85^-^ |
| **Zγ**PG**CC**RN**O**A**C**VKHR**C*** | 2070.85^-^ |

**Table S10-** PIF Toxiforms

| **PIG** | **P** | **C** | **C** | **S** | **N** | **P** | **V** | **C** | **T** | **V** | **H** | **G** | **G** | **P** | **Q** | **L** | **C** |
| --- | --- | --- | --- | --- | --- | --- | --- | --- | --- | --- | --- | --- | --- | --- | --- | --- | --- |
|  |  |  |  |  |  |  |  |  |  |  |  |  |  |  |  |  | ***** |

| Annotated Sequence | MH+ [Da] |
| --- | --- |
| **CC**SNPV**C**TVHGGPQL**C*** | 1844.77 |
| P**CC**SNPV**C**TVHGGPQL**C*** | 1941.82 |

**Table S11-** PIG Toxiforms

| **α-PIVA** | **G** | **C** | **C** | **G** | **S** | **Y** | **P** | **N** | **A** | **A** | **C** | **H** | **P** | **C** | **S** | **C** | **K** | **D** | **R** | **P** | **S** | **Y** | **C** | **G** | **Q** | **G** |
| --- | --- | --- | --- | --- | --- | --- | --- | --- | --- | --- | --- | --- | --- | --- | --- | --- | --- | --- | --- | --- | --- | --- | --- | --- | --- | --- |
|  |  |  |  |  |  | **B** | **O** | **D** |  |  |  |  | **O** |  |  |  |  |  |  | **O** |  |  | ***** | ***** | **E*** | ***** |

| Annotated Sequence | MH+ [Da] |
| --- | --- |
| G**CC**GSYPNAA**C**HP**C**S**C**KDRPSY**C** | 2764.03 |
| G**CC**GSYP**D**AA**C**HP**C**S**C**KDRPSY**C** | 2765.03 |
| G**CC**GSYPNAA**C**HP**C**S**C**KDR**O**SY**C*** | 2779.03 |
| G**CC**GSYPNAA**C**HP**C**S**C**KDR**O**SY**C** | 2780.02^+^ |
| G**CC**GSYPNAA**C**H**OC**S**C**KDRPSY**C** | 2780.02^-^ |
| G**CC**GSYP**D**AA**C**H**OC**S**C**KDRPSY**C** | 2781.00^-^ |
| G**CC**GSYP**D**AA**C**HP**C**S**C**KDR**O**SY**C** | 2781.02^+^ |
| G**CC**GSY**O**NAA**C**H**OC**S**C**KDRPSY**C*** | 2795.03 |
| G**CC**GSY**O**NAA**C**H**OC**S**C**KDRPSY**C** | 2796.01 |
| G**CC**GSYPNAA**C**H**OC**S**C**KDR**O**SY**C** | 2796.01 |
| G**CC**GSY**OD**AA**C**H**OC**S**C**KDRPSY**C** | 2797.00 |
| G**CC**GSY**O**NAA**C**H**OC**S**C**KDR**O**SY**C** | 2812.01 |
| G**CC**GSY**OD**AA**C**H**OC**S**C**KDR**O**SY**C** | 2813.01 |
| G**CC**GSYPNAA**C**HP**C**S**C**KDRPSY**C**G | 2821.05 |
| G**CC**GSYPNAA**C**HP**C**S**C**KDR**O**SY**C**G | 2837.04^+^ |
| G**CC**GSYPNAA**C**H**OC**S**C**KDRPSY**C**G | 2837.04^-^ |
| G**CC**GSYP**D**AA**C**HP**C**S**C**KDR**O**SY**C**G | 2838.04 |
| G**CC**GSYP**D**AA**C**H**OC**S**C**KDRPSY**C**G | 2838.04 |
| G**CC**GSYPNAA**C**H**OC**S**C**KDR**O**SY**C**G | 2853.03^+^ |
| G**CC**GSY**O**NAA**C**H**OC**S**C**KDRPSY**C**G | 2853.04^+^ |
| G**CC**GSY**O**NAA**C**H**OC**S**C**KDR**O**SY**C**G | 2869.03 |
| G**CC**GSY**OD**AA**C**H**OC**S**C**KDR**O**SY**C**G | 2870.03 |
| **CC**GSYPNAA**C**HP**C**S**C**KDRPSY**C**GQ* | 2891.10 |
| **CC**GSYPNAA**C**H**OC**S**C**KDRPSY**C**GQ* | 2907.09 |
| **CC**GSY**O**NAA**C**HP**C**S**C**KDRPSY**C**GQ* | 2907.09 |
| **CC**GSYPNAA**C**HP**C**S**C**KDR**O**SY**C**GQ* | 2907.10 |
| **CC**GSYP**D**AA**C**HP**C**S**C**KDR**O**SY**C**GQ* | 2908.09 |
| **CC**GSY**O**NAA**C**H**OC**S**C**KDRPSY**C**GQ* | 2923.09 |
| **CC**GSYPNAA**C**H**OC**S**C**KDR**O**SY**C**GQ* | 2923.09 |
| **CC**GSY**OD**AA**C**H**OC**S**C**KDRPSY**C**GQ* | 2924.08 |
| **CC**GSYP**D**AA**C**H**OC**S**C**KDR**O**SY**C**GQ* | 2924.09 |
| **CC**GSY**O**NAA**C**H**OC**S**C**KDR**O**SY**C**GQ* | 2939.08 |
| G**CC**GSYPNAA**C**HP**C**S**C**KDRPSY**C**GQ* | 2948.12 |
| G**CC**GSYPNAA**C**HP**C**S**C**KDRPSY**C**GQ | 2949.11 |
| G**CC**GSYPNAA**C**HP**C**S**C**KDRPSY**C**GQ* | 2950.10 |
| G**CC**GSYP**D**AA**C**HP**C**S**C**KDRPSY**C**GQ | 2950.11 |
| **CC**GSYPNAA**C**HP**C**S**C**KDR**O**SY**C**GQG* | 2964.11 |
| G**CC**GSYPNAA**C**H**OC**S**C**KDRPSY**C**GQ* | 2964.11 |
| G**CC**GSYPNAA**C**HP**C**S**C**KDR**O**SY**C**GQ* | 2964.12 |
| G**CC**GSY**O**NAA**C**HP**C**S**C**KDRPSY**C**GQ* | 2964.13 |
| G**CC**GSY**OD**AA**C**HP**C**S**C**KDRPSY**C**GQ* | 2965.10 |
| G**CC**GSYP**D**AA**C**H**OC**S**C**KDRPSY**C**GQ* | 2965.10 |
| G**CC**GSYPNAA**C**HP**C**S**C**KDR**O**SY**C**GQ | 2965.11 |
| G**CC**GSY**O**NAA**C**HP**C**S**C**KDRPSY**C**GQ | 2965.11 |
| G**CC**GSYPNAA**C**H**OC**S**C**KDRPSY**C**GQ | 2965.11 |
| G**CC**GSYP**D**AA**C**HP**C**S**C**KDR**O**SY**C**GQ* | 2965.12 |
| G**CC**GSY**O**NAA**C**HP**C**S**C**KDRPSY**C**GQ* | 2966.10 |
| G**CC**GSY**OD**AA**C**HP**C**S**C**KDRPSY**C**GQ* | 2967.09 |
| **CC**GSYPNAA**C**H**OC**S**C**KDR**O**SY**C**GQG* | 2980.08 |
| **CC**GSY**O**NAA**C**H**OC**S**C**KDRPSY**C**GQG* | 2980.11 |
| G**CC**GSY**O**NAA**C**H**OC**S**C**KDRPSY**C**GQ* | 2980.11 |
| G**CC**GSYPNAA**C**H**OC**S**C**KDR**O**SY**C**GQ* | 2980.11 |
| G**CC**GSYP**D**AA**C**H**OC**S**C**KDR**O**SY**C**GQ* | 2981.10 |
| G**CC**GSY**O**NAA**C**H**OC**S**C**KDRPSY**C**GQ | 2981.10 |
| G**CC**GSYPNAA**C**H**OC**S**C**KDR**O**SY**C**GQ | 2981.10 |
| G**CC**GSY**OD**AA**C**H**OC**S**C**KDRPSY**C**GQ* | 2981.11 |
| G**CC**GSY**O**NAA**C**H**OC**S**C**KDRPSY**C**GQ* | 2982.10 |
| G**CC**GSY**O**NAA**C**H**OC**S**C**KDR**O**SY**C**GQ* | 2996.11 |
| G**CC**GSY**OD**AA**C**H**OC**S**C**KDR**O**SY**C**GQ* | 2997.09 |
| G**CC**GSY**O**NAA**C**H**OC**S**C**KDR**O**SY**C**GQ | 2997.10 |
| G**CC**GSYPNAA**C**HP**C**S**C**KDRPSY**C**GQG* | 3005.14 |
| G**CC**GSYPNAA**C**HP**C**S**C**KDRPSY**C**GQG | 3006.14 |
| G**CC**GSYP**D**AA**C**HP**C**S**C**KDRPSY**C**G**E**G | 3008.10 |
| G**CC**GSYPNAA**C**HP**C**S**C**KDR**O**SY**C**GQG* | 3021.14 |
| G**CC**GSYPNAA**C**H**OC**S**C**KDRPSY**C**GQG* | 3021.14 |
| G**CC**GSY**O**NAA**C**HP**C**S**C**KDRPSY**C**GQG* | 3021.14 |
| G**CC**GSYPNAA**C**H**OC**S**C**KDRPSY**C**GQG | 3022.12^+^ |
| G**CC**GSYPNAA**C**H**OC**S**C**KDRPSY**C**G**E**G* | 3022.12 |
| G**CC**GSYPNAA**C**HP**C**S**C**KDR**O**SY**C**GQG | 3022.13^+^ |
| G**CC**GSYPNAA**C**HP**C**S**C**KDR**O**SY**C**G**E**G* | 3022.13 |
| G**CC**GSYP**D**AA**C**HP**C**S**C**KDR**O**SY**C**GQG* | 3022.13 |
| G**CC**GSYP**D**AA**C**H**OC**S**C**KDRPSY**C**GQG* | 3022.13 |
| G**CC**GSYPNAA**C**HP**C**S**C**KDR**O**SY**C**G**E**G | 3023.12 |
| G**CC**GSYPNAA**C**H**OC**S**C**KDRPSY**C**G**E**G | 3023.12 |
| G**CC**GSYP**D**AA**C**H**OC**S**C**KDRPSY**C**G**E**G* | 3023.12 |
| G**CC**GSYP**D**AA**C**H**OC**S**C**KDRPSY**C**GQG | 3023.12 |
| G**CC**GSYP**D**AA**C**HP**C**S**C**KDR**O**SY**C**G**E**G* | 3023.12 |
| G**CC**GSYP**D**AA**C**HP**C**S**C**KDR**O**SY**C**GQG | 3023.12 |
| G**CC**GSY**O**NAA**C**HP**C**S**C**KDRPSY**C**G**E**G | 3023.12 |
| G**CC**GSY**OD**AA**C**HP**C**S**C**KDRPSY**C**G**E**G | 3024.10 |
| G**CC**GSY**O**NAA**C**H**OC**S**C**KDRPSY**C**GQG* | 3037.13 |
| G**CC**GSYPNAA**C**H**OC**S**C**KDR**O**SY**C**GQG* | 3037.13 |
| G**CC**GSYPNAA**C**H**OC**S**C**KDR**O**SY**C**GQG | 3038.11^-^ |
| G**CC**GSYPNAA**C**H**OC**S**C**KDR**O**SY**C**G**E**G* | 3038.11 |
| G**CC**GSY**O**NAA**C**H**OC**S**C**KDRPSY**C**G**E**G* | 3038.12 |
| G**CC**GSY**O**NAA**C**H**OC**S**C**KDRPSY**C**GQG | 3038.12^+^ |
| G**CC**GSY**OD**AA**C**H**OC**S**C**KDRPSY**C**GQG* | 3038.12 |
| G**CC**GSYP**D**AA**C**H**OC**S**C**KDR**O**SY**C**GQG* | 3038.12 |
| G**CC**GSY**O**NAA**C**H**OC**S**C**KDR**O**SY**C**GQG* | 3053.13 |
| G**CC**GSY**OD**AA**C**H**OC**S**C**KDR**O**SY**C**GQG* | 3054.11 |
| G**CC**GSY**O**NAA**C**H**OC**S**C**KDR**O**SY**C**G**E**G* | 3054.12 |
| G**CC**GSY**O**NAA**C**H**OC**S**C**KDR**O**SY**C**GQG | 3054.12 |
| **CC**GS**BO**NAA**C**H**OC**S**C**KDR**O**SY**C**GQG* | 3074.04 |
| RG**CC**GSYPNAA**C**HP**C**S**C**KDR**O**SY**C**GQ* | 3120.22 |
| RG**CC**GSYP**D**AA**C**HP**C**S**C**KDR**O**SY**C**GQ* | 3121.17 |
| RG**CC**GSYP**D**AA**C**HP**C**S**C**KDR**O**SY**C**GQG* | 3178.19 |
| RG**CC**GSYPNAA**C**HP**C**S**C**KDR**O**SY**C**G**E**G* | 3178.19 |
| RG**CC**GSYPNAA**C**HP**C**S**C**KDR**O**SY**C**GQG | 3178.19 |
| G**CC**GSYPNAA**C**H**OC**S**C**KDRPSY**C**G**E**GR | 3179.20 |
| G**CC**GSYPNAA**C**HP**C**S**C**KDR**O**SY**C**G**E**GR | 3179.20 |
| SHVVRG**CC**GSYP**D**AA**C**HP**C**S**C**KDR**O**SY**C**GQ* | 3543.45 |
| SHVVRG**CC**GSY**O**NAA**C**H**OC**S**C**KDRPSY**C**GQ* | 3558.44 |
| SHVVRG**CC**GSYPNAA**C**H**OC**S**C**KDR**O**SY**C**GQ* | 3558.44 |
| LLSHVVRG**CC**GSY**O**NAA**C**H**OC**S**C**KDRPSY**C**GQ* | 3784.60 |

**Table S12-** α-PIVA Toxiforms

| **κ-PIVE** | **R** | **D** | **C** | **C** | **G** | **V** | **K** | **L** | **E** | **M** | **C** | **H** | **P** | **C** | **L** | **C** | **D** | **N** | **S** | **C** | **K** | **N** | **Y** | **G** | **K** | **G** |
| --- | --- | --- | --- | --- | --- | --- | --- | --- | --- | --- | --- | --- | --- | --- | --- | --- | --- | --- | --- | --- | --- | --- | --- | --- | --- | --- |
|  |  |  |  |  |  |  |  |  | **γ** | **m** |  |  | **O** |  |  |  |  | **D** |  |  |  | **D** | ***** | ***** | ***** | ***** |

| Annotated Sequence | MH+ [Da] |
| --- | --- |
| D**CC**GVKLEM**C**HP**C**L**C**DNS**C**KN | 2657.02 |
| D**CC**GVKLEM**C**HP**C**L**C**D**D**S**C**KN | 2658.00 |
| D**CC**GVKLEM**C**HP**C**L**C**DNS**C**K**D** | 2658.00 |
| D**CC**GVKLE**mC**HP**C**L**C**DNS**C**KN | 2673.01 |
| D**CC**GVKLEM**C**H**OC**L**C**DNS**C**KN | 2673.01 |
| D**CC**GVKLE**mC**H**OC**L**C**DNS**C**KN | 2689.01 |
| D**CC**GVKL**γ**M**C**HP**C**L**C**DNS**C**KN | 2701.02 |
| **CC**GVKLEM**C**HP**C**L**C**DNS**C**KNY | 2705.06 |
| **CC**GVKLEM**C**HP**C**L**C**DNS**C**KNYG | 2762.07 |
| D**CC**GVKLEM**C**HP**C**L**C**DNS**C**KNY* | 2819.10 |
| D**CC**GVKLEM**C**HP**C**L**C**DNS**C**KNY | 2820.08 |
| D**CC**GVKLEM**C**HP**C**L**C**D**D**S**C**K**D**Y* | 2821.07 |
| D**CC**GVKLEM**C**HP**C**L**C**D**D**S**C**KNY | 2821.07 |
| D**CC**GVKLE**mC**HP**C**L**C**DNS**C**KNY | 2836.08 |
| D**CC**GVKLEM**C**H**OC**L**C**DNS**C**K**D**Y* | 2836.08 |
| D**CC**GVKLEM**C**H**OC**L**C**DNS**C**KNY | 2836.08 |
| D**CC**GVKLE**mC**HP**C**L**C**DNS**C**K**D**Y* | 2836.09 |
| D**CC**GVKLE**mC**HP**C**L**C**D**D**S**C**KNY* | 2836.09 |
| D**CC**GVKL**γ**M**C**HP**C**L**C**DNS**C**KNY | 2864.09 |
| D**CC**GVKL**γ**M**C**HP**C**L**C**DNS**C**K**D**Y* | 2864.09 |
| D**CC**GVKLEM**C**HP**C**L**C**DNS**C**KNYG | 2877.10 |
| D**CC**GVKLEM**C**HP**C**L**C**D**D**S**C**KNYG | 2878.09 |
| D**CC**GVKLEM**C**HP**C**L**C**D**D**S**C**K**D**YG* | 2878.09 |
| D**CC**GVKLEM**C**HP**C**L**C**DNS**C**K**D**YG | 2878.09 |
| **CC**GVKLEM**C**HP**C**L**C**DNS**C**KNYGK* | 2889.19 |
| **CC**GVKLEM**C**HP**C**L**C**DNS**C**KNYGK | 2890.19 |
| D**CC**GVKLE**mC**HP**C**L**C**DNS**C**K**D**YG* | 2893.10 |
| D**CC**GVKLE**mC**HP**C**L**C**DNS**C**KNYG | 2893.10 |
| D**CC**GVKLEM**C**H**OC**L**C**DNS**C**KNYG | 2893.10 |
| D**CC**GVKLEM**C**H**OC**L**C**DNS**C**K**D**YG* | 2893.10 |
| D**CC**GVKL**γ**M**C**HP**C**L**C**DNS**C**KNYG* | 2920.11 |
| D**CC**GVKL**γ**M**C**HP**C**L**C**DNS**C**K**D**YG* | 2921.09 |
| D**CC**GVKL**γ**M**C**HP**C**L**C**DNS**C**KNYG | 2921.11 |
| **CC**GVKL**γ**M**C**HP**C**L**C**DNS**C**KNYGK* | 2933.19 |
| D**CC**GVKLEM**C**HP**C**L**C**DNS**C**KNYGK* | 3004.21 |
| D**CC**GVKLEM**C**HP**C**L**C**DNS**C**KNYGK | 3005.20 |
| D**CC**GVKLEM**C**HP**C**L**C**D**D**S**C**K**D**YGK* | 3006.20 |
| D**CC**GVKLE**mC**HP**C**L**C**DNS**C**KNYGK* | 3020.20 |
| D**CC**GVKLEM**C**H**OC**L**C**DNS**C**KNYGK* | 3020.20 |
| D**CC**GVKLEM**C**H**OC**L**C**DNS**C**K**D**YGK* | 3021.20^+^ |
| D**CC**GVKLE**mC**HP**C**L**C**DNS**C**K**D**YGK* | 3021.20^+^ |
| D**CC**GVKLE**mC**HP**C**L**C**D**D**S**C**KNYGK* | 3021.21^+^ |
| D**CC**GVKLEM**C**H**OC**L**C**D**D**S**C**KNYGK* | 3021.21^+^ |
| D**CC**GVKLE**mC**H**OC**L**C**DNS**C**KNYGK* | 3036.20 |
| D**CC**GVKLE**mC**H**OC**L**C**D**D**S**C**KNYGK* | 3037.21 |
| D**CC**GVKLE**mC**H**OC**L**C**D**D**S**C**K**D**YGK* | 3038.20 |
| D**CC**GVKL**γ**M**C**HP**C**L**C**DNS**C**KNYGK* | 3048.20 |
| D**CC**GVKL**γ**M**C**HP**C**L**C**DNS**C**K**D**YGK* | 3049.21^+^ |
| D**CC**GVKL**γ**M**C**HP**C**L**C**D**D**S**C**KNYGK* | 3049.21^+^ |
| D**CC**GVKLEM**C**HP**C**L**C**DNS**C**KNYGKG* | 3061.23 |
| D**CC**GVKLEM**C**HP**C**L**C**DNS**C**KNYGKG | 3062.22 |
| D**CC**GVKL**γmC**HP**C**L**C**DNS**C**KNYGK* | 3064.22 |
| D**CC**GVKL**γ**M**C**H**OC**L**C**DNS**C**KNYGK* | 3064.22 |
| D**CC**GVKLE**mC**HP**C**L**C**DNS**C**KNYGKG* | 3077.23 |
| D**CC**GVKL**γmC**H**OC**L**C**DNS**C**KNYGK* | 3080.21 |
| D**CC**GVKL**γ**M**C**HP**C**L**C**DNS**C**KNYGKG* | 3105.24 |
| RD**CC**GVKLEM**C**HP**C**L**C**D**D**S**C**K**D**YGK* | 3162.25 |
| RD**CC**GVKLE**mC**HP**C**L**C**DNS**C**KNYGK* | 3176.28 |
| RD**CC**GVKLEM**C**H**OC**L**C**DNS**C**KNYGK* | 3176.28 |
| RD**CC**GVKLEM**C**H**OC**L**C**D**D**S**C**KNYGK* | 3177.27 |
| RD**CC**GVKLE**mC**HP**C**L**C**D**D**S**C**KNYGK* | 3177.28 |
| RD**CC**GVKLE**mC**H**OC**L**C**DNS**C**KNYGK* | 3192.27 |
| RD**CC**GVKLE**mC**H**OC**L**C**D**D**S**C**KNYGK* | 3193.26 |
| RD**CC**GVKL**γmC**HP**C**L**C**DNS**C**KNYGK* | 3220.28 |
| D**CC**GVKLEM**C**HP**C**L**C**DNS**C**KNYGKGKKEY* | 3609.53 |
| D**CC**GVKLEM**C**HP**C**L**C**DNS**C**KNYGKGKKEY | 3610.53 |
| D**CC**GVKLEM**C**HP**C**L**C**D**D**S**C**K**D**YGKGKKEY* | 3611.53 |
| D**CC**GVKL**γ**M**C**HP**C**L**C**DNS**C**KNYGKGKKEY* | 3653.54 |
| NAAVNDKASHLIDNVIRD**CC**GVKLEM**C**HP**C**L**C**DNS**C**KNYGK* | 4835.18 |

**Table S13-** κ-PIVE Toxiforms

| κ-PIVF | D | C | C | G | V | K | L | E | M | C | H | P | C | L | C | D | N | S | C | K | K | S | G | K |
| --- | --- | --- | --- | --- | --- | --- | --- | --- | --- | --- | --- | --- | --- | --- | --- | --- | --- | --- | --- | --- | --- | --- | --- | --- |
|  |  |  |  |  |  |  |  | **γ** | **O** |  |  | **O** |  |  |  |  | **D** |  |  |  | ***** | ***** | ***** | ***** |

| Annotated Sequence | MH+ [Da] |
| --- | --- |
| D**CC**GVKLEM**C**HP**C**L**C**DNS**C**KK | 2671.07 |
| D**CC**GVKLEM**C**HP**C**L**C**D**D**S**C**KK | 2672.05 |
| D**CC**GVKLE**mC**HP**C**L**C**DNS**C**KK | 2687.06 |
| D**CC**GVKLE**mC**HP**C**L**C**D**D**S**C**KK* | 2687.06 |
| D**CC**GVKLE**mC**HP**C**L**C**D**D**S**C**KK | 2688.07 |
| D**CC**GVKLEM**C**H**OC**L**C**D**D**S**C**KK | 2688.07 |
| D**CC**GVKL**γ**M**C**HP**C**L**C**DNS**C**KK | 2715.06 |
| D**CC**GVKL**γ**M**C**HP**C**L**C**D**D**S**C**KK | 2716.06 |
| D**CC**GVKL**γmC**HP**C**L**C**DNS**C**KK | 2731.07 |
| D**CC**GVKLEM**C**HP**C**L**C**DNS**C**KKS* | 2757.12 |
| D**CC**GVKLEM**C**HP**C**L**C**DNS**C**KKS | 2758.10 |
| D**CC**GVKLE**mC**HP**C**L**C**DNS**C**KKS* | 2773.11 |
| D**CC**GVKL**γ**M**C**HP**C**L**C**DNS**C**KKS* | 2801.12 |
| D**CC**GVKL**γ**M**C**HP**C**L**C**DNS**C**KKS | 2802.11 |
| D**CC**GVKLEM**C**HP**C**L**C**DNS**C**KKSG* | 2814.13 |
| D**CC**GVKLEM**C**HP**C**L**C**DNS**C**KKSG | 2815.12 |
| D**CC**GVKLEM**C**HP**C**L**C**D**D**S**C**KKSG | 2816.12 |
| **CC**GVKLEM**C**HP**C**L**C**DNS**C**KKSGK* | 2827.20 |
| **CC**GVKLEM**C**HP**C**L**C**DNS**C**KKSGK | 2828.21 |
| D**CC**GVKL**γ**M**C**HP**C**L**C**DNS**C**KKSG* | 2858.14 |
| D**CC**GVKLEM**C**HP**C**L**C**DNS**C**KKSGK* | 2942.23 |
| D**CC**GVKLEM**C**HP**C**L**C**DNS**C**KKSGK | 2943.24 |
| D**CC**GVKLEM**C**H**OC**L**C**DNS**C**KKSGK* | 2958.22^+^ |
| D**CC**GVKLE**mC**HP**C**L**C**DNS**C**KKSGK* | 2958.23^+^ |
| D**CC**GVKLEM**C**H**OC**L**C**D**D**S**C**KKSGK* | 2959.23 |
| D**CC**GVKLE**mC**HP**C**L**C**D**D**S**C**KKSGK* | 2959.23 |
| D**CC**GVKLE**mC**H**OC**L**C**DNS**C**KKSGK* | 2974.22 |
| D**CC**GVKLE**mC**H**OC**L**C**D**D**S**C**KKSGK* | 2975.22 |
| D**CC**GVKL**γ**M**C**HP**C**L**C**DNS**C**KKSGK* | 2986.23 |
| D**CC**GVKL**γ**M**C**HP**C**L**C**D**D**S**C**KKSGK* | 2987.23 |
| D**CC**GVKL**γmC**HP**C**L**C**DNS**C**KKSGK* | 3002.23^+^ |
| D**CC**GVKL**γ**M**C**H**OC**L**C**DNS**C**KKSGK* | 3002.24^+^ |

**Table S14-** κ-PIVF Toxiforms

| **PIVH** | **R** | **D** | **C** | **C** | **G** | **V** | **V** | **M** | **E** | **E** | **C** | **H** | **K** | **C** | **L** | **C** | **N** | **Q** | **T** | **C** | **K** | **K** | **K** | **G** |
| --- | --- | --- | --- | --- | --- | --- | --- | --- | --- | --- | --- | --- | --- | --- | --- | --- | --- | --- | --- | --- | --- | --- | --- | --- |
|  |  |  |  |  |  |  |  | **O** | **γ** | **γ** |  |  |  |  |  |  | **D** | **E** |  | ***** | ***** | ***** | ***** | ***** |

| Annotated Sequence | MH+ [Da] |
| --- | --- |
| D**CC**GVVMEE**C**HK**C**L**C**NQT**C** | 2459.90 |
| D**CC**GVVMEE**C**HK**C**L**C**N**E**T**C** | 2460.90 |
| D**CC**GVVMEE**C**HK**C**L**CD**QT**C** | 2460.90 |
| D**CC**GVV**m**EE**C**HK**C**L**C**NQT**C** | 2475.89 |
| D**CC**GVVM**γ**E**C**HK**C**L**C**NQT**C*** | 2502.90 |
| D**CC**GVVME**γC**HK**C**L**C**NQT**C*** | 2502.90 |
| D**CC**GVVME**γC**HK**C**L**C**NQT**C** | 2503.91 |
| D**CC**GVVM**γ**E**C**HK**C**L**C**NQT**C** | 2503.91 |
| D**CC**GVVMEE**C**HK**C**L**C**NQT**C**K | 2587.99 |
| D**CC**GVVMEE**C**HK**C**L**CD**QT**C**K | 2589.00 |
| D**CC**GVVMEE**C**HK**C**L**C**N**E**T**C**K | 2589.00 |
| D**CC**GVVM**γ**E**C**HK**C**L**C**NQT**C**K* | 2631.00 |
| D**CC**GVVME**γC**HK**C**L**C**NQT**C**K* | 2631.00 |
| D**CC**GVVME**γC**HK**C**L**C**NQT**C**K | 2632.00 |
| D**CC**GVVM**γ**E**C**HK**C**L**C**NQT**C**K | 2632.00 |
| D**CC**GVVMEE**C**HK**C**L**C**NQT**C**KK* | 2715.11 |
| D**CC**GVVMEE**C**HK**C**L**C**NQT**C**KK | 2716.09 |
| D**CC**GVVMEE**C**HK**C**L**C**N**E**T**C**KK | 2717.09 |
| D**CC**GVVMEE**C**HK**C**L**CD**QT**C**KK | 2717.09 |
| **CC**GVVMEE**C**HK**C**L**C**NQT**C**KKK* | 2728.17 |
| **CC**GVVMEE**C**HK**C**L**C**NQT**C**KKK | 2729.18 |
| D**CC**GVVME**γC**HK**C**L**C**NQT**C**KK* | 2759.10 |
| D**CC**GVVM**γ**E**C**HK**C**L**C**NQT**C**KK* | 2759.10 |
| D**CC**GVVM**γ**E**C**HK**C**L**C**NQT**C**KK | 2760.10 |
| **CC**GVV**mγγC**HK**C**L**C**NQT**C**KKK* | 2832.18 |
| D**CC**GVVMEE**C**HK**C**L**C**NQT**C**KKK* | 2843.20 |
| D**CC**GVVMEE**C**HK**C**L**C**NQT**C**KKK | 2844.20 |
| D**CC**GVV**m**EE**C**HK**C**L**C**NQT**C**KKK* | 2859.19 |
| D**CC**GVV**m**EE**C**HK**C**L**C**N**E**T**C**KKK* | 2860.20 |
| D**CC**GVV**m**EE**C**HK**C**L**CD**QT**C**KKK* | 2860.20 |
| D**CC**GVV**m**EE**C**HK**C**L**CDE**T**C**KKK* | 2861.18 |
| D**CC**GVVM**γ**E**C**HK**C**L**C**NQT**C**KKK* | 2887.19 |
| D**CC**GVVME**γC**HK**C**L**C**NQT**C**KKK* | 2887.19 |
| D**CC**GVVME**γC**HK**C**L**CD**QT**C**KKK* | 2888.20 |
| D**CC**GVVM**γ**E**C**HK**C**L**CD**QT**C**KKK* | 2888.20 |
| D**CC**GVVMEE**C**HK**C**L**C**NQT**C**KKKG* | 2900.22 |
| D**CC**GVVMEE**C**HK**C**L**C**NQT**C**KKKG | 2901.23 |
| D**CC**GVVMEE**C**HK**C**L**CDE**T**C**KKKG* | 2902.20 |
| D**CC**GVVMEE**C**HK**C**L**CD**QT**C**KKKG | 2902.20^-^ |
| D**CC**GVVMEE**C**HK**C**L**C**N**E**T**C**KKKG | 2902.20^-^ |
| D**CC**GVV**m**EE**C**HK**C**L**C**NQT**C**KKKG* | 2916.22 |
| D**CC**GVV**m**EE**C**HK**C**L**CD**QT**C**KKKG | 2918.20 |
| D**CC**GVV**m**EE**C**HK**C**L**C**N**E**T**C**KKKG | 2918.20 |
| D**CC**GVV**m**EE**C**HK**C**L**CDE**T**C**KKKG* | 2918.20 |
| D**CC**GVVME**γC**HK**C**L**C**NQT**C**KKKG* | 2944.22^-^ |
| D**CC**GVVM**γ**E**C**HK**C**L**C**NQT**C**KKKG* | 2944.22^-^ |
| RD**CC**GVV**m**EE**C**HK**C**L**C**NQT**C**KKK* | 3015.27 |

**Table S15—**PIVH Toxiforms

| **LINEAR-P** | **F** | **Q** | **P** | **S** | **A** | **E** | **N** | **E** | **E** | **G** | **K** | **F** | **R** | **F** | **F** | **D** | **K** | **Q** | **Q** |
| --- | --- | --- | --- | --- | --- | --- | --- | --- | --- | --- | --- | --- | --- | --- | --- | --- | --- | --- | --- |
|  |  | **Z** | **O** |  |  |  |  |  |  |  |  |  |  |  |  |  |  | **E** | ***** |

| Annotated Sequence | MH+ [Da] |
| --- | --- |
| **Z**PSAENEEGKFRFFDKQ | 2039.94 |
| **ZO**SAENEEGKFRFFDKQ | 2055.94 |
| **Z**PSAENEEGKFRFFDKQQ | 2168.00^+^ |
| **Z**PSAENEEGKFRFFDK**E**Q* | 2168.00^-^ |
| FQPSAENEEGKFRFFDKQ | 2204.04 |
| FQPSAENEEGKFRFFDKQQ | 2332.10 |

**Table S16-** Linear-P Toxiforms

| **Ile-Contryphan-P** | **A** | **T** | **S** | **L** | **G** | **C** | **V** | **I** | **W** | **P** | **W** | **C** |
| --- | --- | --- | --- | --- | --- | --- | --- | --- | --- | --- | --- | --- |
|  |  |  |  |  |  |  |  |  |  | **O** |  | * |

| Annotated Sequence | MH+ [Da] |
| --- | --- |
| **C**VIWPW**C** | 1020.44 |
| **C**VIW**O**W**C** | 1036.44 |
| G**C**VIWPW**C*** | 1076.48 |
| G**C**VIWPW**C** | 1077.46 |
| G**C**VIW**O**W**C** | 1093.46 |
| LG**C**VIWPW**C** | 1190.55 |
| SLG**C**VIWPW**C** | 1277.58 |
| ATSLG**C**VIWPW**C** | 1449.66 |

**Table S17-** Ile-Contryphan-P Toxiforms

| **Contryphan-P3** | **A** | **T** | **S** | **L** | **A** | **C** | **A** | **I** | **W** | **T** | **K** | **C** |
| --- | --- | --- | --- | --- | --- | --- | --- | --- | --- | --- | --- | --- |
|  |  |  |  |  |  |  |  |  |  |  |  |  |

| Annotated Sequence | MH+ [Da] |
| --- | --- |
| **C**AIWTK**C** | 938.42 |
| A**C**AIWTK**C** | 1009.46 |
| ATSLA**C**AIWTK**C** | 1381.66 |

**Table S18-** Contryphan-P3 Toxiforms

| Contryphan-P4 | C | V | Y | W | R | K | C |
| --- | --- | --- | --- | --- | --- | --- | --- |

| Annotated Sequence | MH+ [Da] |
| --- | --- |
| **C**VYWRK**C** | 1071.49 |

**Table S19-** Contryphan-P4 Toxiforms

| **Ψ-PIIIE** | **R** | **H** | **P** | **P** | **C** | **C** | **L** | **Y** | **G** | **K** | **C** | **R** | **R** | **Y** | **P** | **G** | **C** | **S** | **S** | **A** | **S** | **C** | **C** | **Q** | **R** |
| --- | --- | --- | --- | --- | --- | --- | --- | --- | --- | --- | --- | --- | --- | --- | --- | --- | --- | --- | --- | --- | --- | --- | --- | --- | --- |
|  |  |  | **O** | **O** |  |  |  |  |  |  |  |  |  |  | **O** |  |  |  |  |  |  |  |  | **E** | ***** |

| Annotated Sequence | MH+ [Da] |
| --- | --- |
| **OCC**LYGK**C**RRY**O**G**C**SSAS**CC**Q | 2659.04 |
| **CC**LYGK**C**RRY**O**G**C**SSAS**CC**QR* | 2701.11 |
| H**OOCC**LYGK**C**RRY**O**G**C**SSAS**CC** | 2781.09 |
| P**CC**LYGK**C**RRYPG**C**SSAS**CC**QR* | 2782.17 |
| P**CC**LYGK**C**RRY**O**G**C**SSAS**CC**QR* | 2798.16 |
| **OCC**LYGK**C**RRYPG**C**SSAS**CC**QR* | 2798.16 |
| **OCC**LYGK**C**RRY**O**G**C**SSAS**CC**QR* | 2814.16 |
| **OCC**LYGK**C**RRY**O**G**C**SSAS**CCE**R* | 2815.16 |
| **OCC**LYGK**C**RRY**O**G**C**SSAS**CC**QR | 2815.16 |
| H**OOCC**LYGK**C**RRY**O**G**C**SSAS**CC**Q | 2909.16 |
| **OOCC**LYGK**C**RRY**O**G**C**SSAS**CC**QR* | 2927.20 |
| **OOCC**LYGK**C**RRY**O**G**C**SSAS**CCE**R* | 2928.20 |
| **OOCC**LYGK**C**RRY**O**G**C**SSAS**CC**QR | 2928.20 |
| HPP**CC**LYGK**C**RRYPG**C**SSAS**CC**QR* | 3016.28 |
| HP**OCC**LYGK**C**RRYPG**C**SSAS**CC**QR* | 3032.27^+^ |
| H**O**P**CC**LYGK**C**RRYPG**C**SSAS**CC**QR* | 3032.28^+^ |
| HPP**CC**LYGK**C**RRY**O**G**C**SSAS**CC**QR* | 3032.28^-^ |
| H**OOCC**LYGK**C**RRYPG**C**SSAS**CC**QR* | 3048.27 |
| HP**OCC**LYGK**C**RRY**O**G**C**SSAS**CC**QR* | 3048.27 |
| H**OOCC**LYGK**C**RRY**O**G**C**SSAS**CC**QR* | 3064.26 |
| H**OOCC**LYGK**C**RRY**O**G**C**SSAS**CCE**R* | 3065.26 |
| H**OOCC**LYGK**C**RRY**O**G**C**SSAS**CC**QR | 3065.26 |
| RHP**OCC**LYGK**C**RRY**O**G**C**SSAS**CC**QR* | 3204.37 |
| RH**OOCC**LYGK**C**RRYPG**C**SSAS**CC**QR* | 3204.37 |
| RH**OOCC**LYGK**C**RRY**O**G**C**SSAS**CC**QR* | 3220.38 |
| RH**OOCC**LYGK**C**RRY**O**G**C**SSAS**CCE**R* | 3221.36 |
| RH**OOCC**LYGK**C**RRY**O**G**C**SSAS**CC**QR | 3221.36 |

**Table S20-** ψ-PIIIE Toxiforms

| **PIIIG** | **Q** | **W** | **G** | **C** | **C** | **P** | **V** | **N** | **A** | **C** | **R** | **S** | **C** | **H** | **C** | **C** |
| --- | --- | --- | --- | --- | --- | --- | --- | --- | --- | --- | --- | --- | --- | --- | --- | --- |
|  | **Z** |  |  |  |  |  |  | **D** |  |  |  |  |  |  |  | ***** |

| Annotated Sequence | MH+ [Da] |
| --- | --- |
| **Z**WG**CC**PVNA**C**RS**C**H**CC*** | 2093.75 |
| **Z**WG**CC**PV**D**A**C**RS**C**H**CC*** | 2094.74 |

**Table S21-** PIIIG Toxiforms

| **PIIIH** | **E** | **I** | **I** | **L** | **P** | **A** | **L** | **G** | **Q** | **R** | **K** | **C** | **C** | **P** | **L** | **T** | **A** | **C** | **K** | **L** | **G** | **S** | **G** | **C** | **K** | **C** | **C** | **E** |
| --- | --- | --- | --- | --- | --- | --- | --- | --- | --- | --- | --- | --- | --- | --- | --- | --- | --- | --- | --- | --- | --- | --- | --- | --- | --- | --- | --- | --- |
|  | **Z** |  |  |  |  |  |  |  |  |  |  |  |  | **O** |  |  |  |  |  |  |  |  |  |  |  |  |  |  |

| Annotated Sequence | MH+ [Da] |
| --- | --- |
| K**CC**PLTA**C**KLGSG**C**K**CC** | 2059.87 |
| **CC**PLTA**C**KLGSG**C**K**CC**E | 2060.81 |
| K**CC**PLTA**C**KLGSG**C**K**CC**E | 2188.91 |
| K**CCO**LTA**C**KLGSG**C**K**CC**E | 2204.90 |
| LGQRK**CC**PLTA**C**KLGSG**C**K**CC**E | 2643.17 |
| **Z**IILPALGQRK**CC**PLTA**C**KLGSG**C**K**CC**E | 3261.55 |
| EIILPALGQRK**CC**PLTA**C**KLGSG**C**K**CC**E | 3279.56 |

**Table S22-** PIIIH Toxiforms

| **PIIII** | **C** | **C** | **Q** | **A** | **Y** | **C** | **S** | **R** | **Y** | **H** | **C** | **L** | **P** | **C** | **C** |
| --- | --- | --- | --- | --- | --- | --- | --- | --- | --- | --- | --- | --- | --- | --- | --- |

| Annotated Sequence | MH+ [Da] |
| --- | --- |
| **CC**QAY**C**SRYH**C**LP**CC** | 2094.75 |

**Table S23-** PIIII Toxiforms

| δ-PVIA | E | A | C | Y | A | P | G | T | F | C | G | I | K | P | G | L | C | C | S | E | F | C | L | P | G | V | C | F | G |
| --- | --- | --- | --- | --- | --- | --- | --- | --- | --- | --- | --- | --- | --- | --- | --- | --- | --- | --- | --- | --- | --- | --- | --- | --- | --- | --- | --- | --- | --- |
|  |  |  |  |  |  | **O** |  |  |  |  |  |  |  | **O** |  |  |  |  |  |  |  |  |  |  |  |  |  |  | ***** |

| Annotated Sequence | MH+ [Da] |
| --- | --- |
| EA**C**YA**O**GTF**C**GIK**O**GL**CC**SEF**C**LPGV**C** | 3142.29 |
| **C**YA**O**GTF**C**GIK**O**GL**CC**SEF**C**LPGV**C**FG* | 3145.31 |
| EASKLDKKEA**C**YA**O**GTF**C**GIK**O**GL**CC**SEF**C**LPGV**C**FG* | 4244.90 |

**Table S24-** δ-PVIA Toxiforms

| **PVIB** | **Q** | **C** | **T** | **P** | **Y** | **G** | **G** | **S** | **C** | **G** | **V** | **D** | **S** | **T** | **C** | **C** | **G** | **R** | **C** | **N** | **V** | **P** | **R** | **N** | **K** | **C** | **E** | **L** |
| --- | --- | --- | --- | --- | --- | --- | --- | --- | --- | --- | --- | --- | --- | --- | --- | --- | --- | --- | --- | --- | --- | --- | --- | --- | --- | --- | --- | --- |
|  | **Z** |  |  | **O** |  |  |  |  |  |  |  |  |  |  |  |  |  |  |  | **D** |  | **O** |  | **D** |  |  | **γ*** | ***** |

| Annotated Sequence | MH+ [Da] |
| --- | --- |
| **ZC**TPYGGS**C**GVDST**CC**GR**C**NVPR**D**K**C*** | 3033.20 |
| **ZC**TPYGGS**C**GVDST**CC**GR**C**NVPRNK**C** | 3033.20 |
| **ZC**TPYGGS**C**GVDST**CC**GR**CD**VPRNK**C** | 3034.20 |
| **ZC**TPYGGS**C**GVDST**CC**GR**CD**VPR**D**K**C*** | 3034.20 |
| **ZC**T**O**YGGS**C**GVDST**CC**GR**C**NVPR**D**K**C*** | 3049.19 |
| **ZC**T**O**YGGS**C**GVDST**CC**GR**C**NVPRNK**C** | 3049.19 |
| **ZC**T**O**YGGS**C**GVDST**CC**GR**CD**VPR**D**K**C*** | 3050.19 |
| **ZC**T**O**YGGS**C**GVDST**CC**GR**CD**VPRNK**C** | 3050.19 |
| **C**TPYGGS**C**GVDST**CC**GR**C**NVPRNK**C**E | 3051.21 |
| **C**T**O**YGGS**C**GVDST**CC**GR**C**NVPRNK**C**E | 3067.20 |
| **C**T**O**YGGS**C**GVDST**CC**GR**C**NVPR**D**K**C**E* | 3067.20 |
| **C**T**O**YGGS**C**GVDST**CC**GR**C**NV**O**RNK**C**E | 3083.20 |
| **C**T**O**YGGS**C**GVDST**CC**GR**C**NV**O**R**D**K**C**E* | 3083.20 |
| **C**T**O**YGGS**C**GVDST**CC**GR**CD**V**O**RNK**C**E* | 3083.20 |
| **ZC**TPYGGS**C**GVDST**CC**GR**C**NVPR**D**K**C**E* | 3162.24 |
| **ZC**TPYGGS**C**GVDST**CC**GR**CD**VPRNK**C**E* | 3162.24 |
| **ZC**TPYGGS**C**GVDST**CC**GR**C**NVPRNK**C**E | 3162.25 |
| **ZC**TPYGGS**C**GVDST**CC**GR**CD**VPR**D**K**C**E* | 3163.24 |
| **ZC**TPYGGS**C**GVDST**CC**GR**CD**VPRNK**C**E | 3163.24 |
| **ZC**TPYGGS**C**GVDST**CC**GR**C**NVPR**D**K**C**E | 3163.24 |
| **ZC**TPYGGS**C**GVDST**CC**GR**CD**VPR**D**K**C**E | 3164.23 |
| **ZC**T**O**YGGS**C**GVDST**CC**GR**C**NVPRNK**C**E | 3178.23 |
| **ZC**T**O**YGGS**C**GVDST**CC**GR**C**NVPR**D**K**C**E* | 3178.23 |
| **ZC**TPYGGS**C**GVDST**CC**GR**C**NV**O**R**D**K**C**E* | 3178.23 |
| **ZC**TPYGGS**C**GVDST**CC**GR**C**NV**O**RNK**C**E | 3178.23 |
| **ZC**TPYGGS**C**GVDST**CC**GR**CD**V**O**RNK**C**E* | 3178.23 |
| **ZC**TPYGGS**C**GVDST**CC**GR**C**NV**O**R**D**K**C**E | 3179.23 |
| **ZC**TPYGGS**C**GVDST**CC**GR**CD**V**O**RNK**C**E | 3179.23 |
| **ZC**TPYGGS**C**GVDST**CC**GR**CD**V**O**R**D**K**C**E* | 3179.23 |
| **ZC**T**O**YGGS**C**GVDST**CC**GR**CD**VPRNK**C**E | 3179.24 |
| **ZC**T**O**YGGS**C**GVDST**CC**GR**CD**VPR**D**K**C**E* | 3179.24 |
| **ZC**T**O**YGGS**C**GVDST**CC**GR**C**NVPR**D**K**C**E | 3179.24 |
| Q**C**TPYGGS**C**GVDST**CC**GR**C**NVPRNK**C**E | 3179.24 |
| **ZC**T**O**YGGS**C**GVDST**CC**GR**CD**VPR**D**K**C**E | 3180.22 |
| Q**C**TPYGGS**C**GVDST**CC**GR**C**NVPR**D**K**C**E | 3180.22 |
| **ZC**TPYGGS**C**GVDST**CC**GR**CD**V**O**R**D**K**C**E | 3180.22 |
| Q**C**TPYGGS**C**GVDST**CC**GR**CD**VPR**D**K**C**E* | 3180.24 |
| Q**C**TPYGGS**C**GVDST**CC**GR**CD**VPRNK**C**E | 3180.24 |
| **ZC**TPYGGS**C**GVDST**CC**GR**C**NVPRNK**C**E | 3180.24 |
| **ZC**TPYGGS**C**GVDST**CC**GR**C**NVPR**D**K**C**E* | 3180.24 |
| Q**C**TPYGGS**C**GVDST**CC**GR**CD**VPR**D**K**C**E | 3181.21 |
| **ZC**T**O**YGGS**C**GVDST**CC**GR**C**NV**O**RNK**C**E* | 3193.25 |
| **ZC**T**O**YGGS**C**GVDST**CC**GR**C**NV**O**RNK**C**E | 3194.23 |
| **ZC**T**O**YGGS**C**GVDST**CC**GR**C**NV**O**R**D**K**C**E* | 3194.23 |
| **ZC**T**O**YGGS**C**GVDST**CC**GR**CD**V**O**RNK**C**E* | 3194.24 |
| **ZC**T**O**YGGS**C**GVDST**CC**GR**C**NV**O**R**D**K**C**E | 3195.22 |
| **ZC**T**O**YGGS**C**GVDST**CC**GR**CD**V**O**RNK**C**E | 3195.23 |
| Q**C**TPYGGS**C**GVDST**CC**GR**C**NV**O**R**D**K**C**E* | 3195.23 |
| Q**C**TPYGGS**C**GVDST**CC**GR**C**NV**O**RNK**C**E | 3195.23 |
| **ZC**T**O**YGGS**C**GVDST**CC**GR**C**NVPRNK**Cγ*** | 3222.22 |
| **ZC**TPYGGS**C**GVDST**CC**GR**C**NV**O**RNK**Cγ** | 3222.25 |
| **ZC**T**O**YGGS**C**GVDST**CC**GR**CD**VPRNK**Cγ** | 3223.22 |
| **ZC**TPYGGS**C**GVDST**CC**GR**C**NVPR**D**K**C**EL | 3276.28 |
| **ZC**TPYGGS**C**GVDST**CC**GR**CD**VPRNK**C**EL | 3276.28 |
| **ZC**TPYGGS**C**GVDST**CC**GR**CD**VPR**D**K**C**EL* | 3276.28 |
| **ZC**TPYGGS**C**GVDST**CC**GR**CD**VPR**D**K**C**EL | 3277.29 |
| **ZC**T**O**YGGS**C**GVDST**CC**GR**C**NVPR**D**K**C**EL | 3292.27^-^ |
| **ZC**T**O**YGGS**C**GVDST**CC**GR**CD**VPR**D**K**C**EL* | 3292.27 |
| **ZC**T**O**YGGS**C**GVDST**CC**GR**CD**VPRNK**C**EL | 3292.27^-^ |
| **ZC**TPYGGS**C**GVDST**CC**GR**CD**V**O**RNK**C**EL | 3292.27^-^ |
| **ZC**TPYGGS**C**GVDST**CC**GR**CD**V**O**R**D**K**C**EL* | 3292.27 |
| **ZC**T**O**YGGS**C**GVDST**CC**GR**CD**VPR**D**K**C**EL | 3293.27 |
| **ZC**T**O**YGGS**C**GVDST**CC**GR**C**NV**O**R**D**K**C**EL | 3308.27 |
| **ZC**T**O**YGGS**C**GVDST**CC**GR**CD**V**O**RNK**C**EL | 3308.27 |
| **ZC**T**O**YGGS**C**GVDST**CC**GR**CD**V**O**R**D**K**C**EL | 3309.27 |
| **ZC**TPYGGS**C**GVDST**CC**GR**C**NV**O**RNK**Cγ**L | 3335.31 |
| **ZC**TPYGGS**C**GVDST**CC**GR**C**NV**O**R**D**K**Cγ**L* | 3335.31 |
| **ZC**TPYGGS**C**GVDST**CC**GR**CD**V**O**RNK**Cγ**L* | 3335.31 |
| **ZC**T**O**YGGS**C**GVDST**CC**GR**CD**VPR**D**K**Cγ**L | 3337.27 |

**Table S25-** PVIB Toxiforms

| **PVIC** | **E** | **A** | **C** | **Y** | **A** | **P** | **G** | **T** | **F** | **C** | **G** | **I** | **K** | **P** | **G** | **L** | **C** | **C** | **S** | **A** | **L** | **C** | **L** | **P** | **A** | **V** | **C** | **I** | **D** |
| --- | --- | --- | --- | --- | --- | --- | --- | --- | --- | --- | --- | --- | --- | --- | --- | --- | --- | --- | --- | --- | --- | --- | --- | --- | --- | --- | --- | --- | --- |
|  | **Z** |  |  |  |  | **O** |  |  |  |  |  |  |  | **O** |  | ***** |  |  |  |  |  |  |  |  |  |  |  |  | ***** |

**Table S26**- PVIC sites of modification **There were no spectral matches to the full PVIC mature peptide. Sites of modification were determined by matches to peptide fragments

| **PVID** | **S** | **N** | **R** | **P** | **C** | **K** | **K** | **S** | **G** | **R** | **K** | **C** | **F** | **P** | **H** | **Q** | **K** | **D** | **C** | **C** | **G** | **R** | **A** | **C** | **I** | **I** | **T** | **I** | **C** | **P** |
| --- | --- | --- | --- | --- | --- | --- | --- | --- | --- | --- | --- | --- | --- | --- | --- | --- | --- | --- | --- | --- | --- | --- | --- | --- | --- | --- | --- | --- | --- | --- |
|  |  | **D** |  | **O** |  |  |  |  |  |  |  |  |  | **O** |  | **E** |  |  |  |  |  |  |  |  |  |  |  |  |  | ***** |

| Annotated Sequence | MH+ [Da] |
| --- | --- |
| SNR**OC**KKSGRK**C**F**O**HQKD**CC**GRA**C**IITI**C**P | 3723.74 |
| SNR**OC**KKSGRK**C**F**O**H**E**KD**CC**GRA**C**IITI**C**P* | 3723.74 |
| S**D**R**OC**KKSGRK**C**F**O**HQKD**CC**GRA**C**IITI**C**P* | 3723.74 |

**Table S27-** PVID Toxiforms

| **PVIE** | **V** | **G** | **E** | **F** | **R** | **G** | **C** | **A** | **H** | **I** | **N** | **Q** | **A** | **C** | **N** | **P** | **P** | **Q** | **C** | **C** | **R** | **G** | **Y** | **T** | **C** | **Q** | **S** | **S** | **Y** | **I** | **P** | **S** | **C** | **Q** | **L** |
| --- | --- | --- | --- | --- | --- | --- | --- | --- | --- | --- | --- | --- | --- | --- | --- | --- | --- | --- | --- | --- | --- | --- | --- | --- | --- | --- | --- | --- | --- | --- | --- | --- | --- | --- | --- |
|  |  |  |  |  |  |  |  |  |  |  | **D** | **E** |  |  | **D** | **O** | **O** | **E** |  |  |  |  |  |  |  |  |  |  |  |  | **O** |  |  | **E** | ***** |

| Annotated Sequence | MH+ [Da] |
| --- | --- |
| G**C**AHINQA**C**N**OO**Q**CC**RGYT**C**QSSYI**O**S**C**QL | 3635.47 |
| G**C**AHINQA**C**N**OO**Q**CC**RGYT**C**QSSYI**O**S**CE**L* | 3635.47 |
| VGEFRG**C**AHINQA**C**NPPQ**CC**RGYT**C**QSSYI**O**S**C**QL | 4191.78 |
| VGEFRG**C**AHINQA**C**NPPQ**CC**RGYT**C**QSSYI**O**S**CE**L* | 4191.78 |
| VGEFRG**C**AHINQA**C**NP**O**Q**CC**RGYT**C**QSSYI**O**S**C**QL | 4207.77^+^ |
| VGEFRG**C**AHINQA**C**NP**O**Q**CC**RGYT**C**QSSYI**O**S**CE**L* | 4207.77 |
| VGEFRG**C**AHINQA**C**NP**OECC**RGYT**C**QSSYI**O**S**C**QL* | 4207.77 |
| VGEFRG**C**AHINQA**CD**P**O**Q**CC**RGYT**C**QSSYI**O**S**C**QL* | 4207.77 |
| VGEFRG**C**AHINQA**C**N**OO**Q**CC**RGYT**C**QSSYIPS**C**QL | 4207.77^-^ |
| VGEFRG**C**AHINQA**C**N**OO**Q**CC**RGYT**C**QSSYIPS**CE**L* | 4207.77 |
| VGEFRG**C**AHINQA**C**N**OO**Q**CC**RGYT**C**QSSYI**O**S**C**QL | 4223.76 |
| VGEFRG**C**AHINQA**C**N**OO**Q**CC**RGYT**C**QSSYI**O**S**CE**L* | 4223.76 |
| VGEFRG**C**AHIN**E**A**C**N**OO**Q**CC**RGYT**C**QSSYI**O**S**C**QL* | 4223.77 |
| VGEFRG**C**AHINQA**CDOO**Q**CC**RGYT**C**QSSYI**O**S**C**QL* | 4223.77 |
| VGEFRG**C**AHI**D**QA**C**N**OO**Q**CC**RGYT**C**QSSYI**O**S**C**QL* | 4223.77 |
| VGEFRG**C**AHINQA**C**N**OOECC**RGYT**C**QSSYI**O**S**C**QL* | 4223.77 |

**Table S28-** PVIE Toxiforms

| PVIF | A | T | S | N | R | P | C | K | K | T | G | R | K | C | F | P | H | Q | K | D | C | C | G | R | A | C | I | I | T | I | C | P |
| --- | --- | --- | --- | --- | --- | --- | --- | --- | --- | --- | --- | --- | --- | --- | --- | --- | --- | --- | --- | --- | --- | --- | --- | --- | --- | --- | --- | --- | --- | --- | --- | --- |
|  |  |  |  | **D** |  | **O** |  |  |  |  |  |  |  |  |  | **O** |  | **E** |  |  |  |  |  |  |  |  |  |  |  |  |  | ***** |

| Annotated Sequence | MH+ [Da] |
| --- | --- |
| ATSNR**OC**KKTGRK**C**F**O**H**E**KD**CC**GRA**C**IITI**C**P* | 3909.83 |
| ATS**D**R**OC**KKTGRK**C**F**O**HQKD**CC**GRA**C**IITI**C**P* | 3909.83 |
| ATSNR**OC**KKTGRK**C**F**O**HQKD**CC**GRA**C**IITI**C**P | 3909.83 |

**Table S29-** PVIF Toxiforms

| **PVIG** | **S** | **T** | **T** | **K** | **G** | **A** | **T** | **S** | **N** | **R** | **P** | **C** | **K** | **I** | **P** | **G** | **R** | **K** | **C** | **F** | **P** | **H** | **Q** | **K** | **D** | **C** | **C** | **G** | **R** | **A** | **C** | **I** | **I** | **T** | **I** | **C** | **P** |
| --- | --- | --- | --- | --- | --- | --- | --- | --- | --- | --- | --- | --- | --- | --- | --- | --- | --- | --- | --- | --- | --- | --- | --- | --- | --- | --- | --- | --- | --- | --- | --- | --- | --- | --- | --- | --- | --- |
|  |  |  |  |  |  |  |  |  | **D** |  | **O** |  |  |  | **O** |  |  |  |  |  | **O** |  | **E** |  |  |  |  |  |  |  |  |  |  |  |  |  | ***** |

| Annotated Sequence | MH+ [Da] |
| --- | --- |
| GATSNR**OC**KI**O**GRK**C**FPHEKD**CC**GRA**C**IITI**C**P* | 3947.85 |
| GATSNRP**C**KI**O**GRK**C**F**O**HEKD**CC**GRA**C**IITI**C**P* | 3947.85 |
| GATSNR**OC**KI**O**GRK**C**FPHQKD**CC**GRA**C**IITI**C**P | 3947.85^+^ |
| GATSNRP**C**KI**O**GRK**C**F**O**HQKD**CC**GRA**C**IITI**C**P | 3947.85^+^ |
| GATSNR**OC**KI**O**GRK**C**FPHEKD**CC**GRA**C**IITI**C**P | 3948.84 |
| GATSNRP**C**KI**O**GRK**C**F**O**HEKD**CC**GRA**C**IITI**C**P | 3948.84 |
| GATS**D**R**OC**KI**O**GRK**C**F**O**HQKD**CC**GRA**C**IITI**C**P* | 3963.83 |
| GATSNR**OC**KI**O**GRK**C**F**O**HEKD**CC**GRA**C**IITI**C**P* | 3963.85 |
| GATSNR**OC**KI**O**GRK**C**F**O**HQKD**CC**GRA**C**IITI**C**P | 3963.85 |
| GATS**D**R**OC**KI**O**GRK**C**F**O**HEKD**CC**GRA**C**IITI**C**P | 3965.82 |
| STTKGATSNR**OC**KI**O**GRK**C**FPHEKD**CC**GRA**C**IITI**C**P* | 4365.07 |
| STTKGATS**D**R**OC**KI**O**GRK**C**FPHQKD**CC**GRA**C**IITI**C**P* | 4365.07 |
| STTKGATS**D**RP**C**KI**O**GRK**C**F**O**HQKD**CC**GRA**C**IITI**C**P* | 4365.07 |
| STTKGATSNR**OC**KI**O**GRK**C**FPHQKD**CC**GRA**C**IITI**C**P | 4365.07 |
| STTKGATSNR**OC**KI**O**GRK**C**F**O**HEKD**CC**GRA**C**IITI**C**P* | 4381.06 |
| STTKGATS**D**R**OC**KI**O**GRK**C**F**O**HQKD**CC**GRA**C**IITI**C**P* | 4381.07 |

**Table S30-** PVIG Toxiforms

| **κ-PVIIA** | **C** | **R** | **I** | **P** | **N** | **Q** | **K** | **C** | **F** | **Q** | **H** | **L** | **D** | **D** | **C** | **C** | **S** | **R** | **K** | **C** | **N** | **R** | **F** | **N** | **K** | **C** | **V** |
| --- | --- | --- | --- | --- | --- | --- | --- | --- | --- | --- | --- | --- | --- | --- | --- | --- | --- | --- | --- | --- | --- | --- | --- | --- | --- | --- | --- |
|  |  |  |  | **O** | **D** | **E** |  |  |  | **E** |  |  |  |  |  |  |  |  |  |  | **D** |  |  | D |  |  | ***** |

| Annotated Sequence | MH+ [Da] |
| --- | --- |
| **C**RIPNQK**C**FQHLDD**CC**SRK**C**NRFNK**C**V | 3600.58 |
| **C**RIPNQK**C**FQHLDD**CC**SRK**CD**RFNK**C**V | 3601.57 |
| **C**RIPNQK**C**FQHLDD**CC**SRK**C**NRF**D**K**C**V | 3601.57 |
| **C**RIPNQK**C**FQHLDD**CC**SRK**CD**RF**D**K**C**V* | 3601.57 |
| **C**RIPNQK**C**F**E**HLDD**CC**SRK**C**NRFNK**C**V | 3601.58 |
| **C**RIP**D**QK**C**FQHLDD**CC**SRK**CD**RFNK**C**V | 3602.57 |
| **C**RI**O**NQK**C**FQHLDD**CC**SRK**CD**RFNK**C**V* | 3616.60 |
| **C**RI**O**NQK**C**FQHLDD**CC**SRK**C**NRF**D**K**C**V* | 3616.60 |
| **C**RI**O**NQK**C**FQHLDD**CC**SRK**C**NRFNK**C**V | 3616.60 |
| **C**RI**OD**QK**C**FQHLDD**CC**SRK**CD**RFNK**C**V* | 3617.59 |
| **C**RI**OD**QK**C**FQHLDD**CC**SRK**C**NRFNK**C**V | 3617.59 |
| **C**RI**O**NQK**C**FQHLDD**CC**SRK**CD**RF**D**K**C**V* | 3617.60 |
| **C**RI**O**NQK**C**FQHLDD**CC**SRK**CD**RFNK**C**V | 3617.60 |
| **C**RI**O**NQK**C**FQHLDD**CC**SRK**C**NRF**D**K**C**V | 3617.60 |
| **C**RI**O**N**E**K**C**FQHLDD**CC**SRK**C**NRFNK**C**V | 3617.60 |
| **C**RI**O**NQK**C**F**E**HLDD**CC**SRK**C**NRFNK**C**V | 3617.61 |
| **C**RI**O**N**E**K**C**FQHLDD**CC**SRK**C**NRF**D**K**C**V* | 3617.61 |
| **C**RI**O**NQK**C**FQHLDD**CC**SRK**CD**RF**D**K**C**V | 3618.59 |

**Table S31-** κ-PVIIA Toxiforms

| **Contryphan-P** | **G** | **C** | **P** | **W** | **D** | **P** | **W** | **C** |
| --- | --- | --- | --- | --- | --- | --- | --- | --- |
|  |  |  | **O** |  |  |  |  | ***** |

| Annotated Sequence | Thγo. MH+ [Da] |
| --- | --- |
| G**CO**WDPW**C*** | 1092.403 |

**Table S32-** Contryphan-P Toxiforms

| PIIA | C | C | C | I | R | S | D | G | P | K | C | S | R | K | C | L | S | S | F | F | C |
| --- | --- | --- | --- | --- | --- | --- | --- | --- | --- | --- | --- | --- | --- | --- | --- | --- | --- | --- | --- | --- | --- |
|  |  |  |  |  |  |  |  |  |  |  |  |  |  |  |  |  |  |  |  |  |  |

| Annotated Sequence | MH+ [Da] |
| --- | --- |
| **CC**IRSDGPK**C**SRK**C**LSSFF**C** | 2525.10 |
| **CCC**IRSDGPK**C**SRK**C**LSSFF**C** | 2685.13 |

**Table S33-** PIIA Toxiforms

| **PVIIIA** | **G** | **C** | **S** | **G** | **S** | **P** | **C** | **F** | **K** | **N** | **K** | **T** | **C** | **R** | **D** | **E** | **C** | **I** | **C** | **G** | **G** | **L** | **S** | **N** | **C** | **W** |
| --- | --- | --- | --- | --- | --- | --- | --- | --- | --- | --- | --- | --- | --- | --- | --- | --- | --- | --- | --- | --- | --- | --- | --- | --- | --- | --- |
|  |  |  |  |  |  | **O** |  |  |  | **D** |  |  |  |  |  | **γ** |  |  |  |  |  |  |  | **D** |  |  |

| **G** | **Y** | **G** | **G** | **S** | **R** | **C** | **G** | **C** | **K** | **C** | **T** | **C** | **R** | **E** |
| --- | --- | --- | --- | --- | --- | --- | --- | --- | --- | --- | --- | --- | --- | --- |
|  |  |  |  |  |  |  |  |  |  |  |  |  |  |  |

| Annotated Sequence | MH+ [Da] |
| --- | --- |
| G**C**SGS**OC**FK**D**KT**C**RDE**C**I**C**GGLSN**C**W**C**GYGGSRG**C**K**C**T**C** | 4630.78^+^ |
| G**C**SGS**OC**FKNKT**C**RDE**C**I**C**GGLS**DC**W**C**GYGGSRG**C**K**C**T**C** | 4630.80^-^ |
| G**C**SGS**OC**FK**D**KT**C**RDE**C**I**C**GGLS**DC**W**C**GYGGSRG**C**K**C**T**C** | 4631.79 |
| G**C**SGS**OC**FK**D**KT**C**RD**γC**I**C**GGLS**DC**W**C**GYGGSRG**C**K**C**T**C** | 4675.78 |
| **C**SGSP**C**FKNKT**C**RDE**C**I**C**GGLSN**C**W**C**GYGGSRG**C**K**C**T**C**RE | 4841.91 |
| **C**SGSP**C**FKNKT**C**RDE**C**I**C**GGLS**DC**W**C**GYGGSRG**C**K**C**T**C**RE | 4842.89^-^ |
| **C**SGSP**C**FK**D**KT**C**RDE**C**I**C**GGLSN**C**W**C**GYGGSRG**C**K**C**T**C**RE | 4842.92^-^ |
| **C**SGSP**C**FK**D**KT**C**RDE**C**I**C**GGLS**DC**W**C**GYGGSRG**C**K**C**T**C**RE | 4843.91 |
| **C**SGS**OC**FKNKT**C**RDE**C**I**C**GGLSN**C**W**C**GYGGSRG**C**K**C**T**C**RE | 4857.92 |
| **C**SGS**OC**FKNKT**C**RDE**C**I**C**GGLS**DC**W**C**GYGGSRG**C**K**C**T**C**RE | 4858.90 |
| **C**SGS**OC**FK**D**KT**C**RDE**C**I**C**GGLSN**C**W**C**GYGGSRG**C**K**C**T**C**RE | 4858.90 |
| **C**SGS**OC**FK**D**KT**C**RDE**C**I**C**GGLS**DC**W**C**GYGGSRG**C**K**C**T**C**RE | 4859.92 |
| **C**SGSP**C**FKNKT**C**RD**γC**I**C**GGLSN**C**W**C**GYGGSRG**C**K**C**T**C**RE | 4885.87 |
| G**C**SGSP**C**FKNKT**C**RDE**C**I**C**GGLSN**C**W**C**GYGGSRG**C**K**C**T**C**RE | 4898.92 |
| G**C**SGSP**C**FKNKT**C**RDE**C**I**C**GGLS**DC**W**C**GYGGSRG**C**K**C**T**C**RE | 4899.93^+^ |
| G**C**SGSP**C**FK**D**KT**C**RDE**C**I**C**GGLSN**C**W**C**GYGGSRG**C**K**C**T**C**RE | 4899.93^+^ |
| G**C**SGSP**C**FK**D**KT**C**RDE**C**I**C**GGLS**DC**W**C**GYGGSRG**C**K**C**T**C**RE | 4900.92 |
| **C**SGS**OC**FKNKT**C**RD**γC**I**C**GGLSN**C**W**C**GYGGSRG**C**K**C**T**C**RE | 4901.90 |
| **C**SGS**OC**FK**D**KT**C**RD**γC**I**C**GGLSN**C**W**C**GYGGSRG**C**K**C**T**C**RE | 4902.93 |
| **C**SGS**OC**FKNKT**C**RD**γC**I**C**GGLS**DC**W**C**GYGGSRG**C**K**C**T**C**RE | 4902.93 |
| **C**SGS**OC**FK**D**KT**C**RD**γC**I**C**GGLS**DC**W**C**GYGGSRG**C**K**C**T**C**RE | 4903.91 |
| G**C**SGS**OC**FKNKT**C**RDE**C**I**C**GGLSN**C**W**C**GYGGSRG**C**K**C**T**C**RE | 4914.93 |
| G**C**SGS**OC**FK**D**KT**C**RDE**C**I**C**GGLSN**C**W**C**GYGGSRG**C**K**C**T**C**RE | 4915.93^+^ |
| G**C**SGS**OC**FKNKT**C**RDE**C**I**C**GGLS**DC**W**C**GYGGSRG**C**K**C**T**C**RE | 4915.94^+^ |
| G**C**SGS**OC**FK**D**KT**C**RDE**C**I**C**GGLS**DC**W**C**GYGGSRG**C**K**C**T**C**RE | 4916.93 |
| G**C**SGSP**C**FKNKT**C**RD**γC**I**C**GGLSN**C**W**C**GYGGSRG**C**K**C**T**C**RE | 4942.91 |
| G**C**SGSP**C**FK**D**KT**C**RD**γC**I**C**GGLSN**C**W**C**GYGGSRG**C**K**C**T**C**RE | 4943.91^+^ |
| G**C**SGSP**C**FKNKT**C**RD**γC**I**C**GGLS**DC**W**C**GYGGSRG**C**K**C**T**C**RE | 4943.92^+^ |
| G**C**SGSP**C**FK**D**KT**C**RD**γC**I**C**GGLS**DC**W**C**GYGGSRG**C**K**C**T**C**RE | 4944.92 |
| G**C**SGS**OC**FKNKT**C**RD**γC**I**C**GGLSN**C**W**C**GYGGSRG**C**K**C**T**C**RE | 4958.93 |
| G**C**SGS**OC**FKNKT**C**RD**γC**I**C**GGLS**DC**W**C**GYGGSRG**C**K**C**T**C**RE | 4959.91^+^ |
| G**C**SGS**OC**FK**D**KT**C**RD**γC**I**C**GGLSN**C**W**C**GYGGSRG**C**K**C**T**C**RE | 4959.92^+^ |
| G**C**SGS**OC**FK**D**KT**C**RD**γC**I**C**GGLS**DC**W**C**GYGGSRG**C**K**C**T**C**RE | 4960.91 |

**Table S34-** PVIIIA Toxiforms

| **PVA** | **G** | **C** | **C** | **P** | **K** | **Q** | **M** | **R** | **C** | **C** | **T** | **L** |
| --- | --- | --- | --- | --- | --- | --- | --- | --- | --- | --- | --- | --- |
|  |  |  |  |  |  |  |  |  |  |  |  | ***** |

| Annotated Sequence | MH+ [Da] |
| --- | --- |
| G**CC**PKQMR**CC**TL | 1570.64 |
| G**CC**PKQMR**CC**TL* | 1569.66 |

**Table 35-** PVA Toxiforms

| **PVB** | **R** | **D** | **C** | **C** | **P** | **E** | **K** | **M** | **W** | **C** | **C** | **P** | **L** |
| --- | --- | --- | --- | --- | --- | --- | --- | --- | --- | --- | --- | --- | --- |
|  |  |  |  |  | **O** |  |  | **O** |  |  |  | **O** | ***** |

| Annotated Sequence | MH+ [Da] |
| --- | --- |
| D**CC**PEKMW**CC**P | 1542.53 |
| D**CCO**EK**m**W**CC**P | 1574.52 |
| D**CCO**EKMW**CC**P | 1558.52 |
| D**CC**PEK**m**W**CC**P | 1558.52 |
| D**CCO**EKMW**CC**PL* | 1670.62 |
| D**CC**PEK**m**W**CC**PL* | 1670.62 |
| D**CC**PEKMW**CC**PL* | 1654.63 |
| D**CCO**EKMW**CCO**L* | 1686.62 |
| D**CCO**EK**m**W**CC**PL* | 1686.62 |
| RD**CC**PEKMW**CC**P | 1698.63 |
| RD**CC**PEKMW**CC**PL* | 1810.73 |

**Table 36-** PVB Toxiforms

| **p21b** | **F** | **E** | **L** | **L** | **P** | **S** | **Q** | **D** | **R** | **S** | **C** | **C** | **I** | **R** | **K** | **T** | **L** | **E** | **C** | **L** | **E** | **N** | **Y** | **P** | **G** | **Q** | **E** | **S** | **Q** | **R** | **A** | **H** | **Y** |
| --- | --- | --- | --- | --- | --- | --- | --- | --- | --- | --- | --- | --- | --- | --- | --- | --- | --- | --- | --- | --- | --- | --- | --- | --- | --- | --- | --- | --- | --- | --- | --- | --- | --- |
| **A** |  |  |  |  | **O** |  | **E** |  |  |  |  |  |  |  |  |  |  |  |  |  |  |  |  | **O** |  |  |  |  | **E** |  |  |  | * |

| **p21b** | **S** | **I** | **N** | **A** | **Q** | **N** | **N** | **V** | **R** | **P** | **A** | **H** | **D** | **T** | **C** | **I** | **N** | **R** | **L** | **C** | **F** | **D** | **P** | **G** | **F** |
| --- | --- | --- | --- | --- | --- | --- | --- | --- | --- | --- | --- | --- | --- | --- | --- | --- | --- | --- | --- | --- | --- | --- | --- | --- | --- |
| **B** |  |  |  |  |  |  |  |  |  | **O** |  |  |  |  |  |  |  |  |  |  |  |  |  |  |  |

**Table S37-** p21b sites of modification on 2 identified peptides (A and B) **There were no spectral matches to the full PVIC mature peptide. Sites of modification were determined by matches to peptide fragments

**
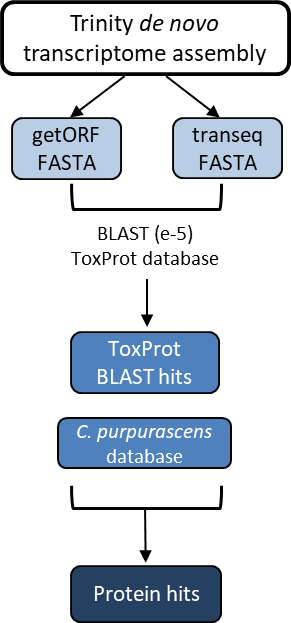
**

**Figure S1-** Workflow of the database search strategy


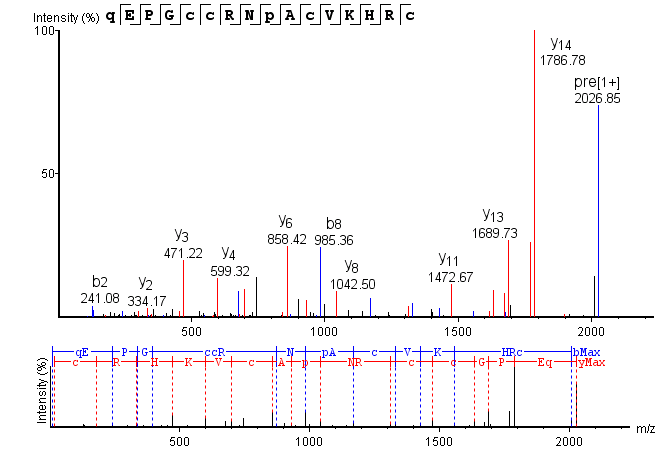


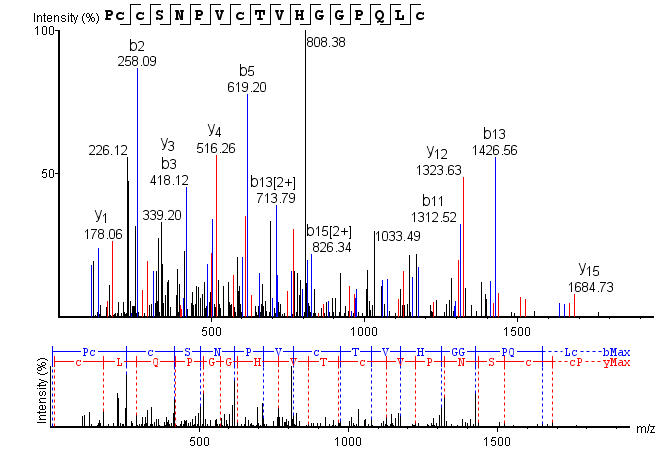


**Figure S2**- MS/MS spectra PIF, PIG, and Contryphan-P4


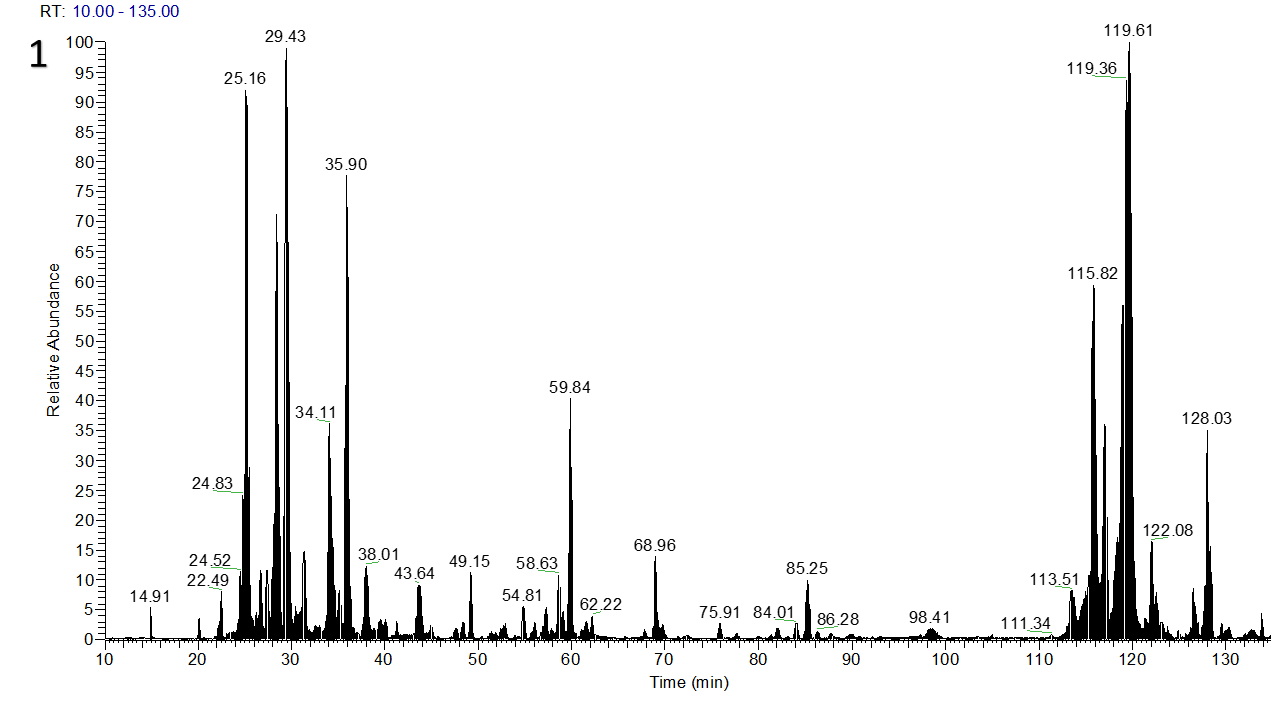


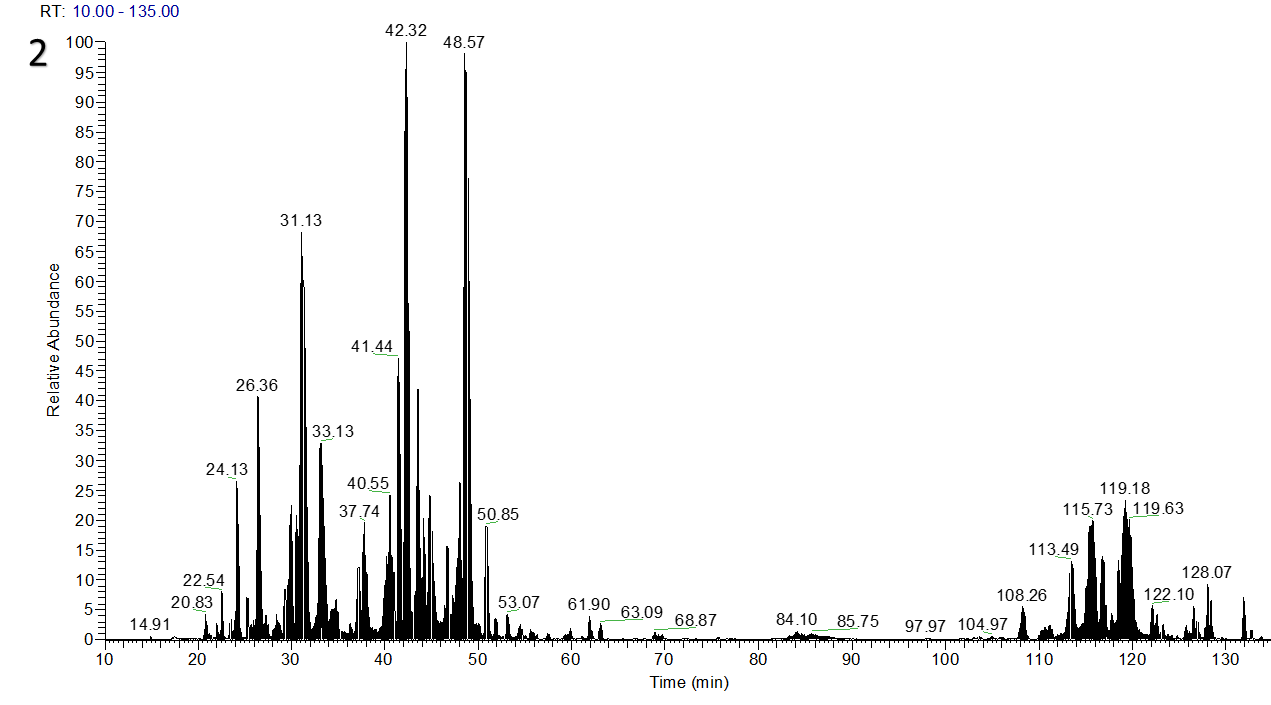


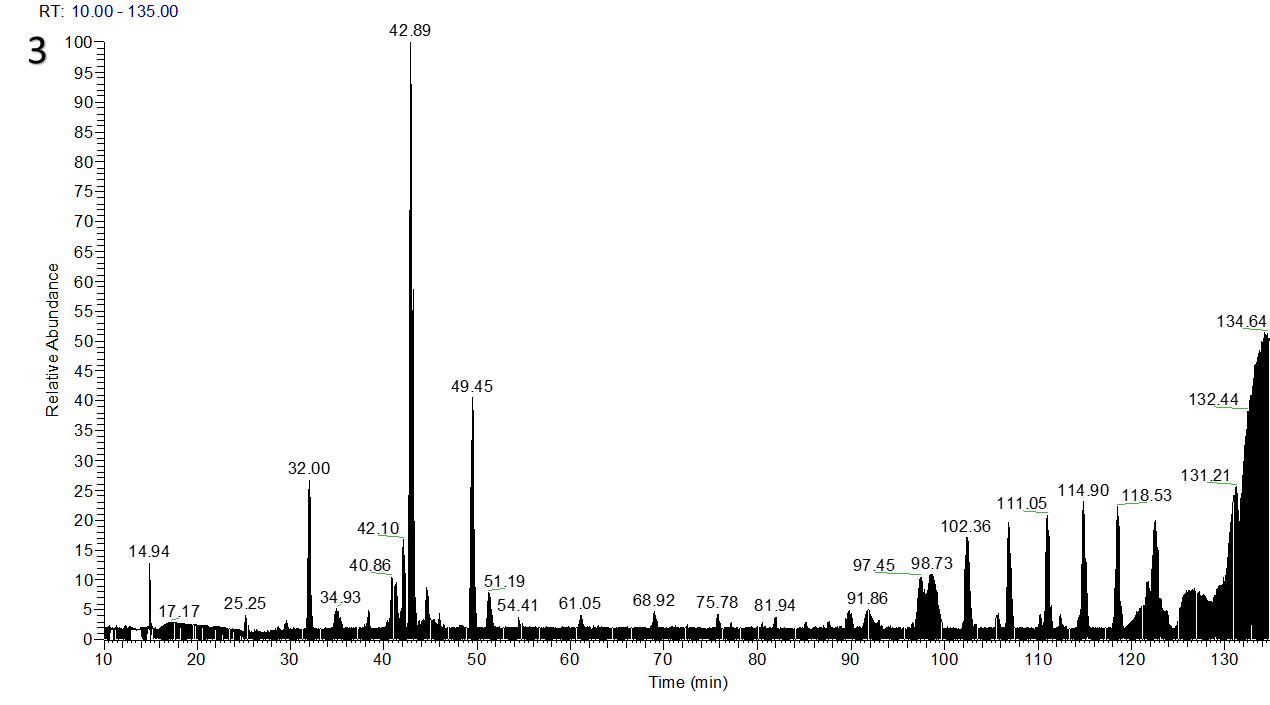


**Figure S3** Chromatograms (TIC) of injected venom samples from *C. purpurascens* specimens 1-27


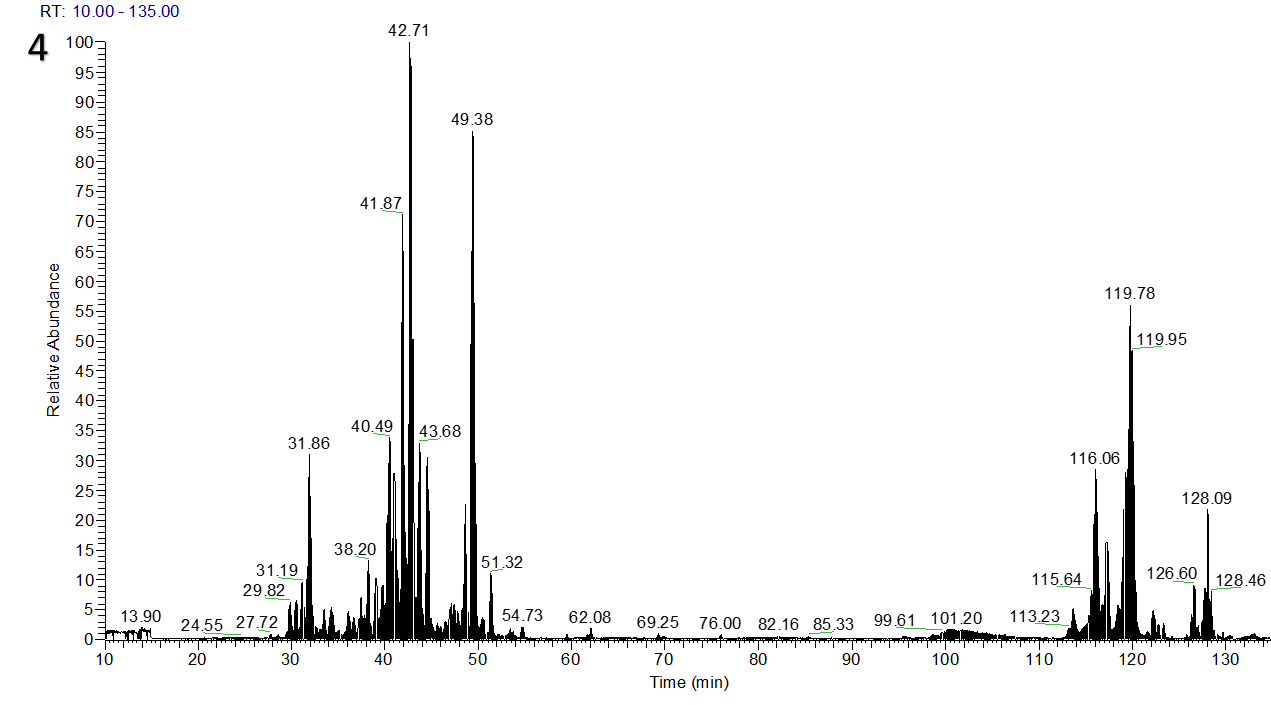


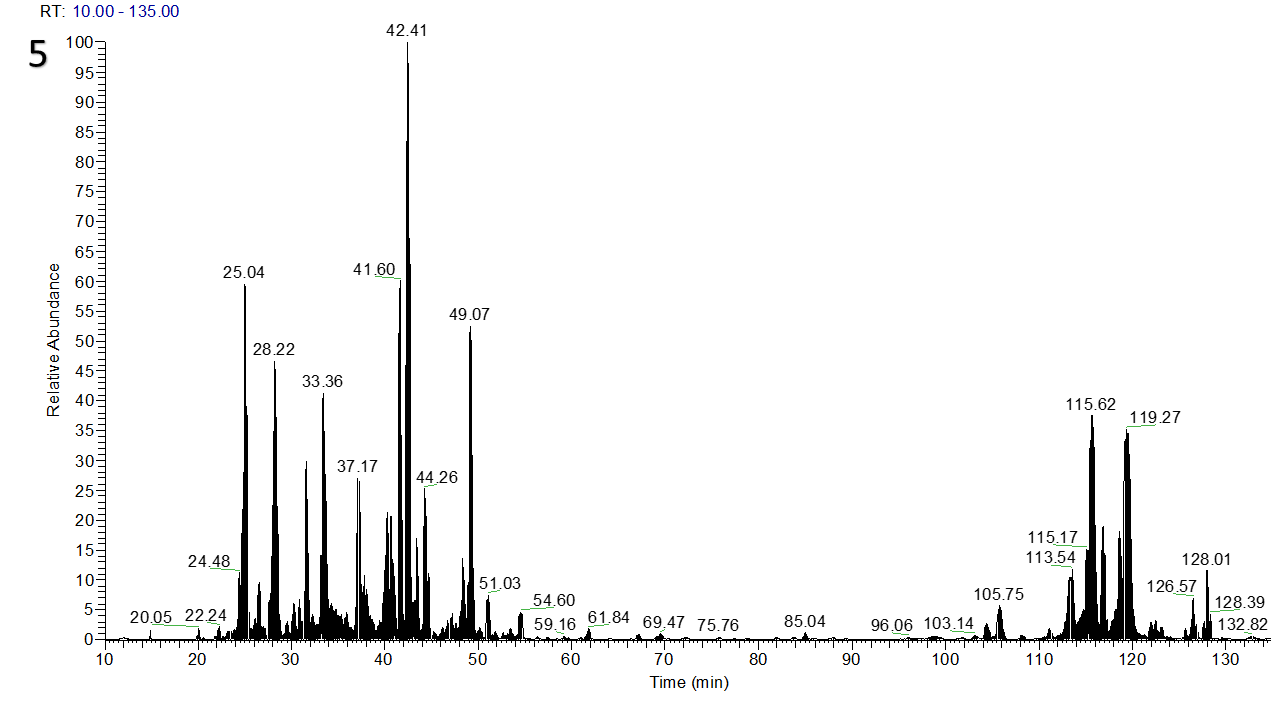


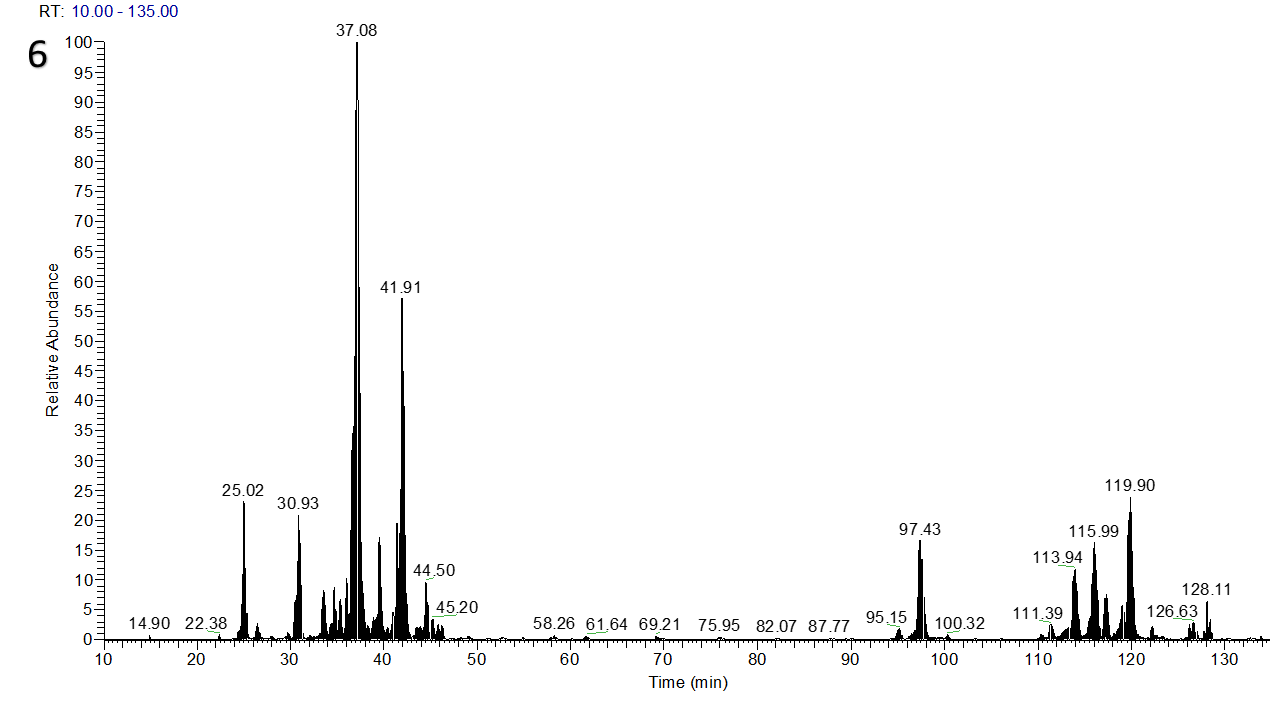


**Figure S3 (Cont.)**


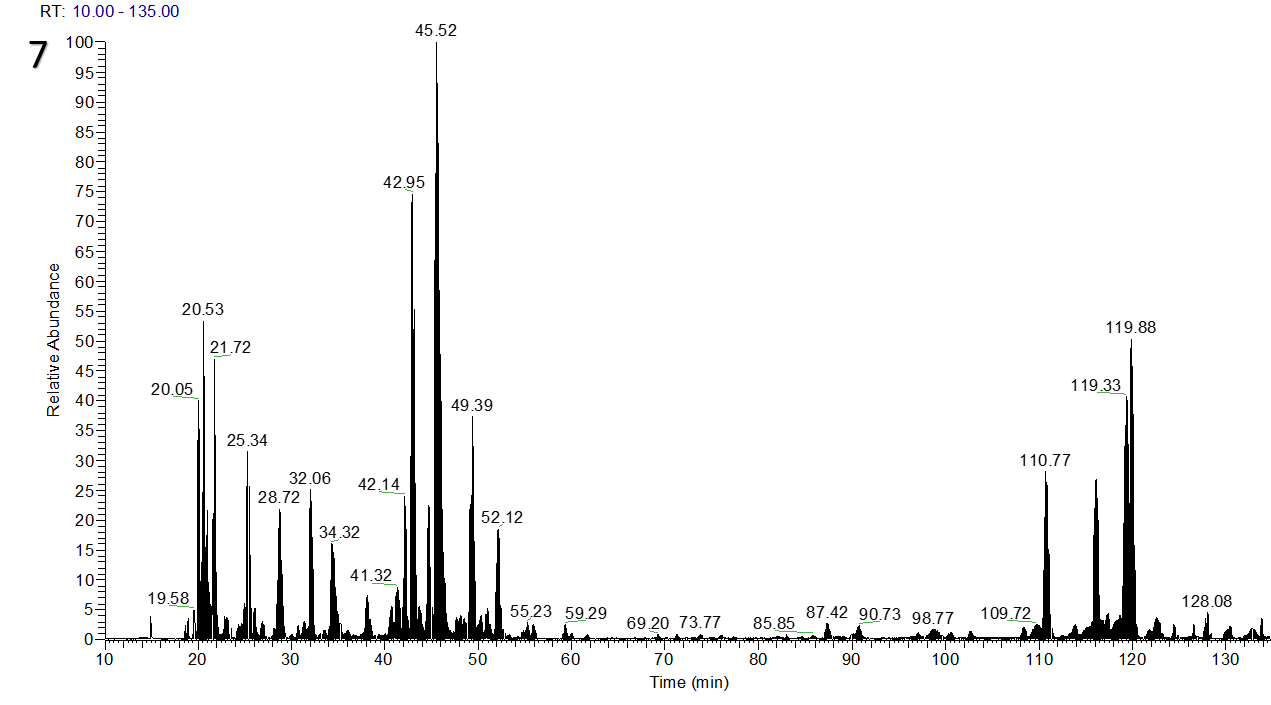


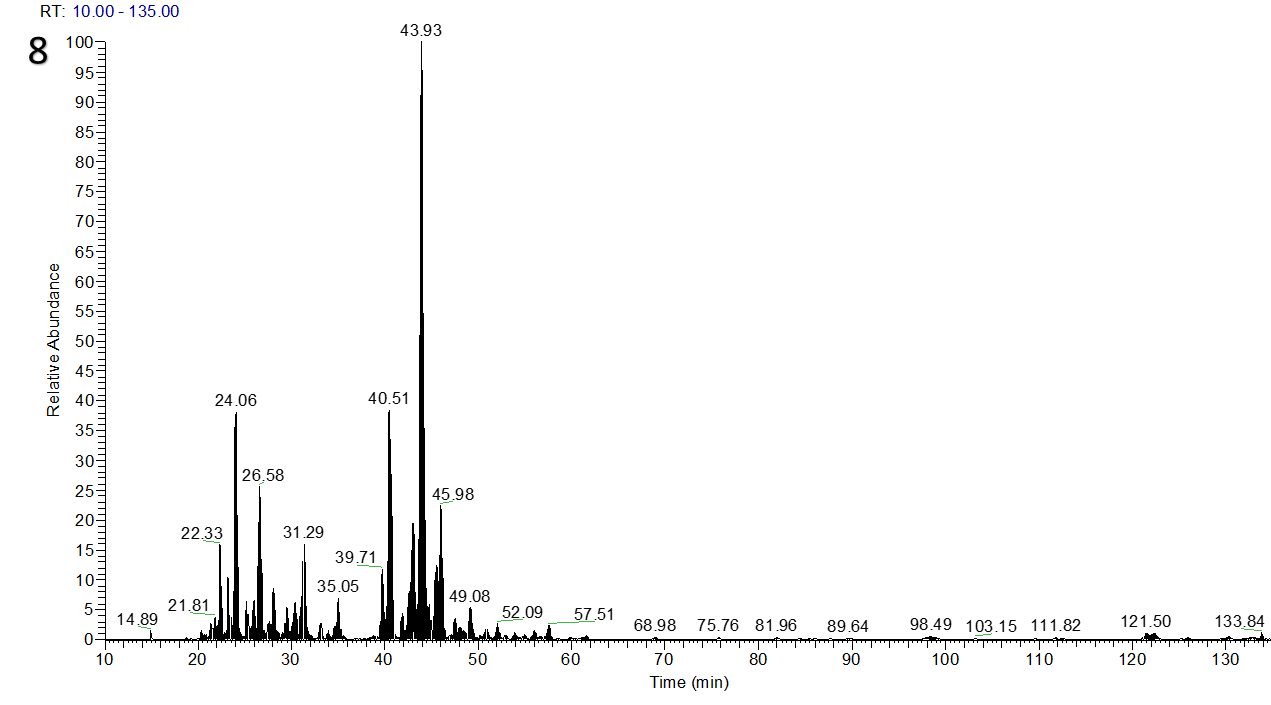

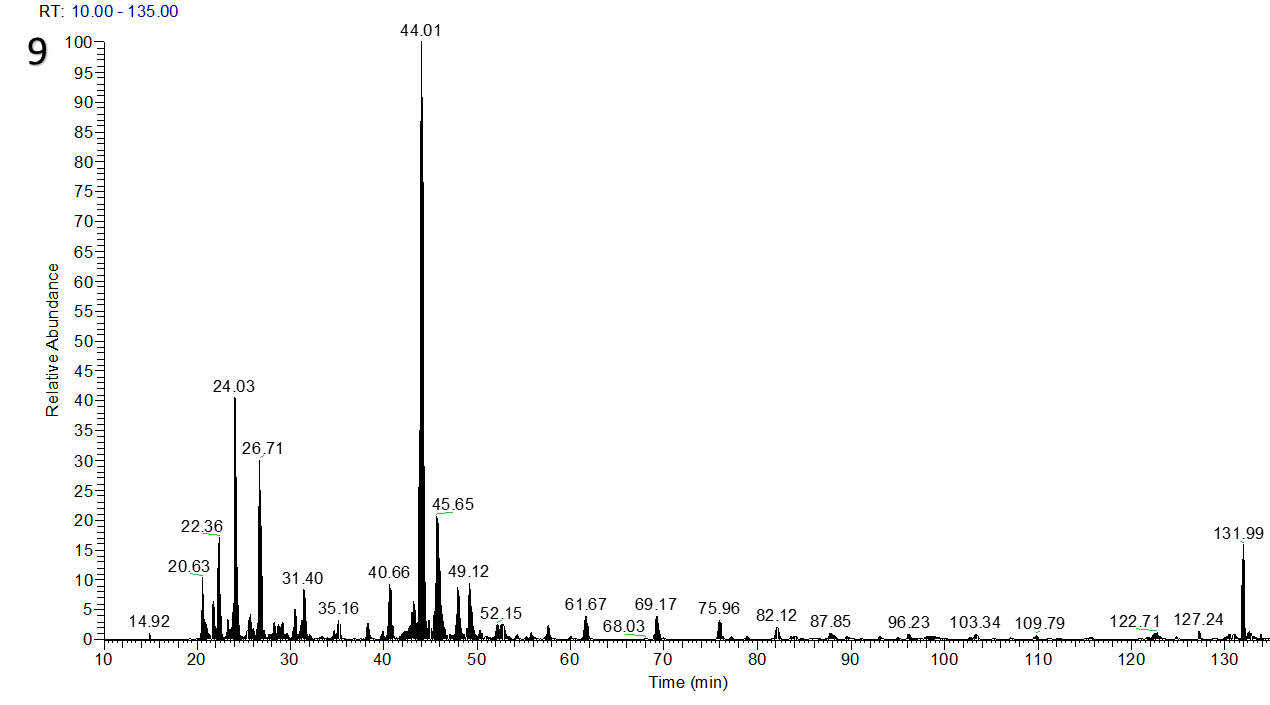


**Figure S3 (Cont.)**


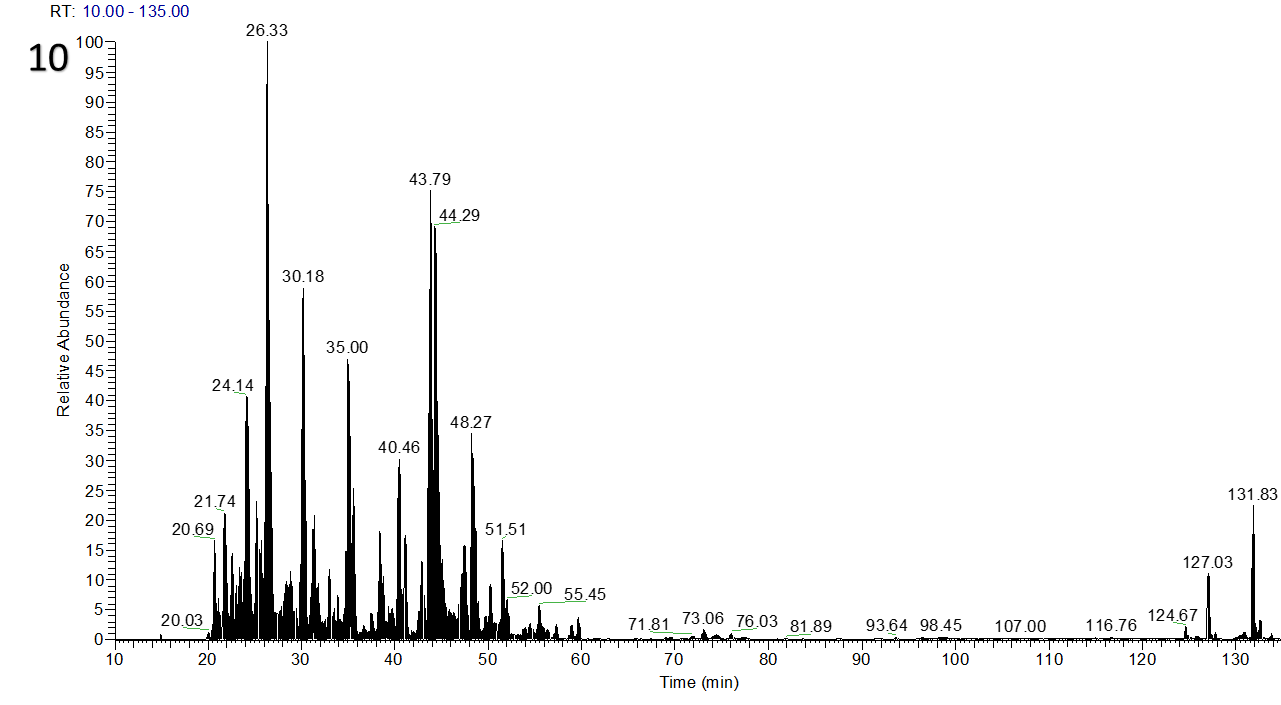


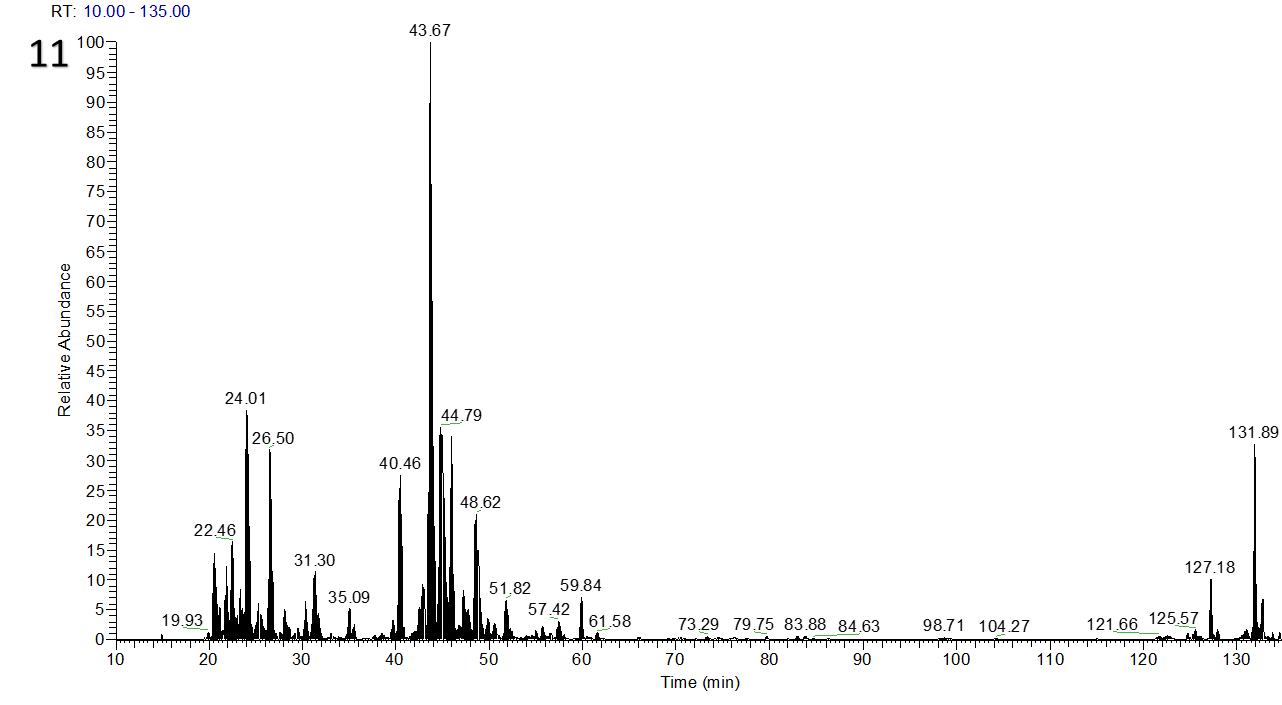


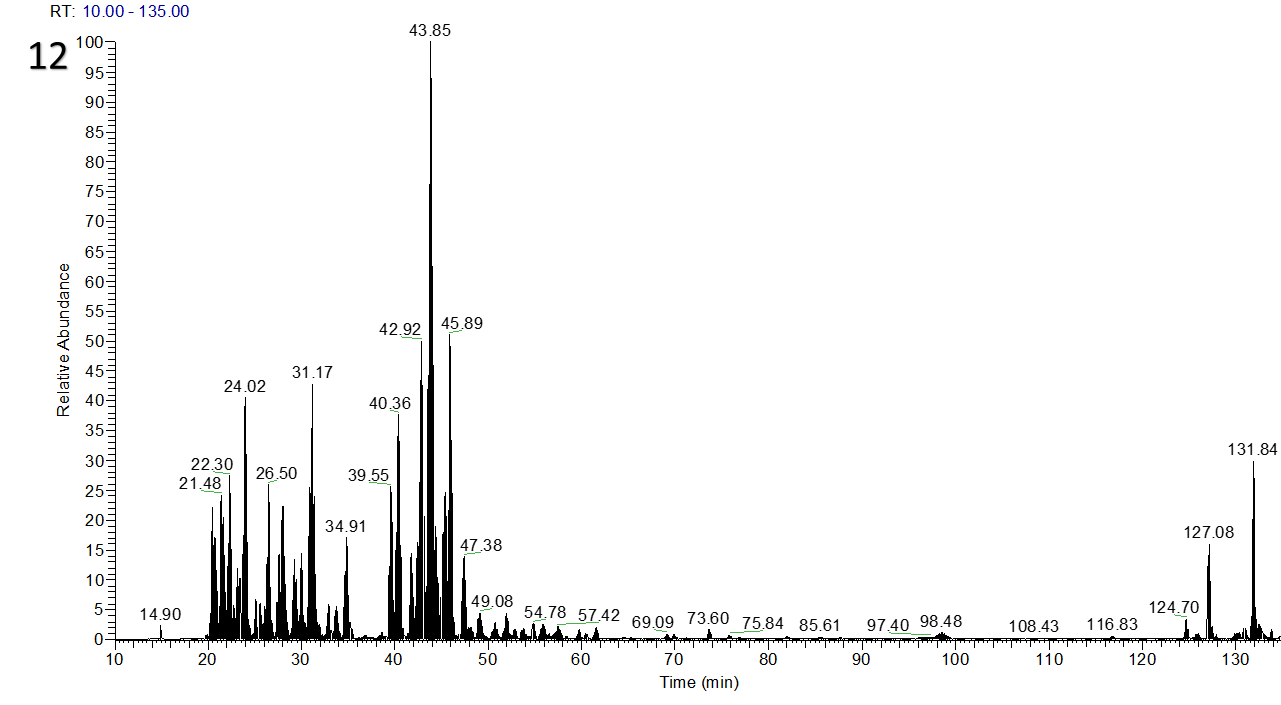


**Figure S3 (Cont.)**


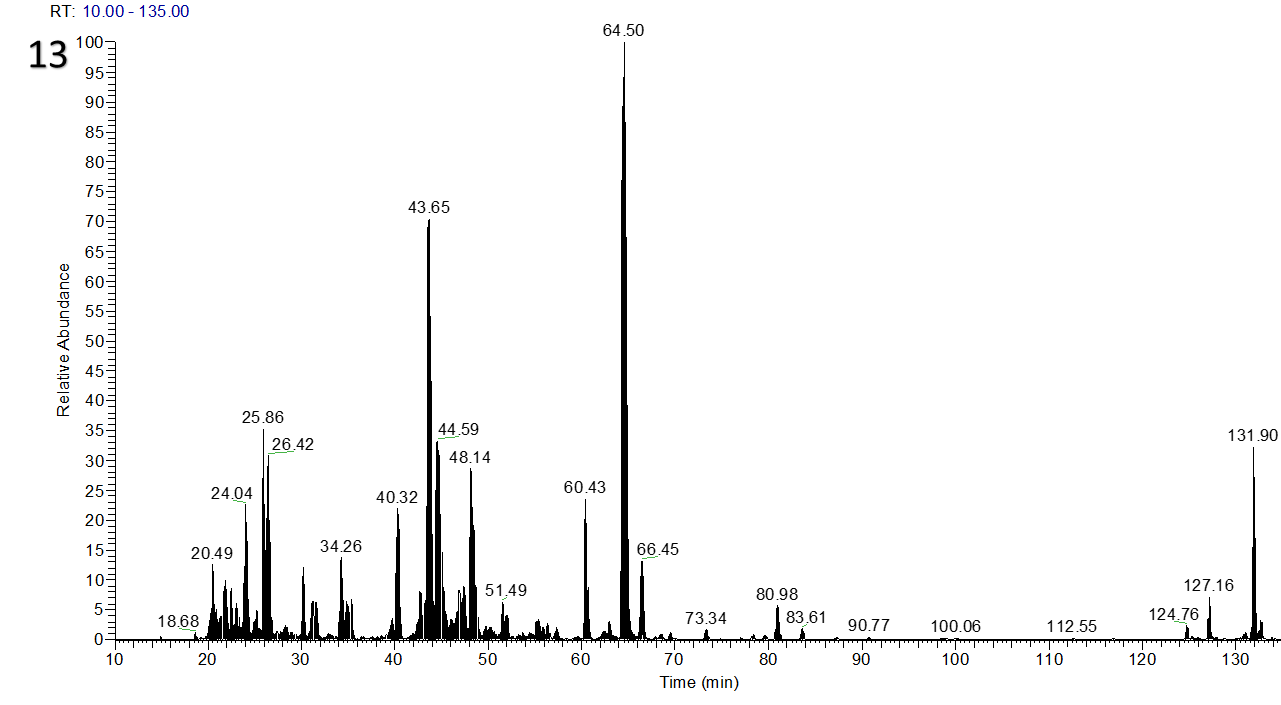


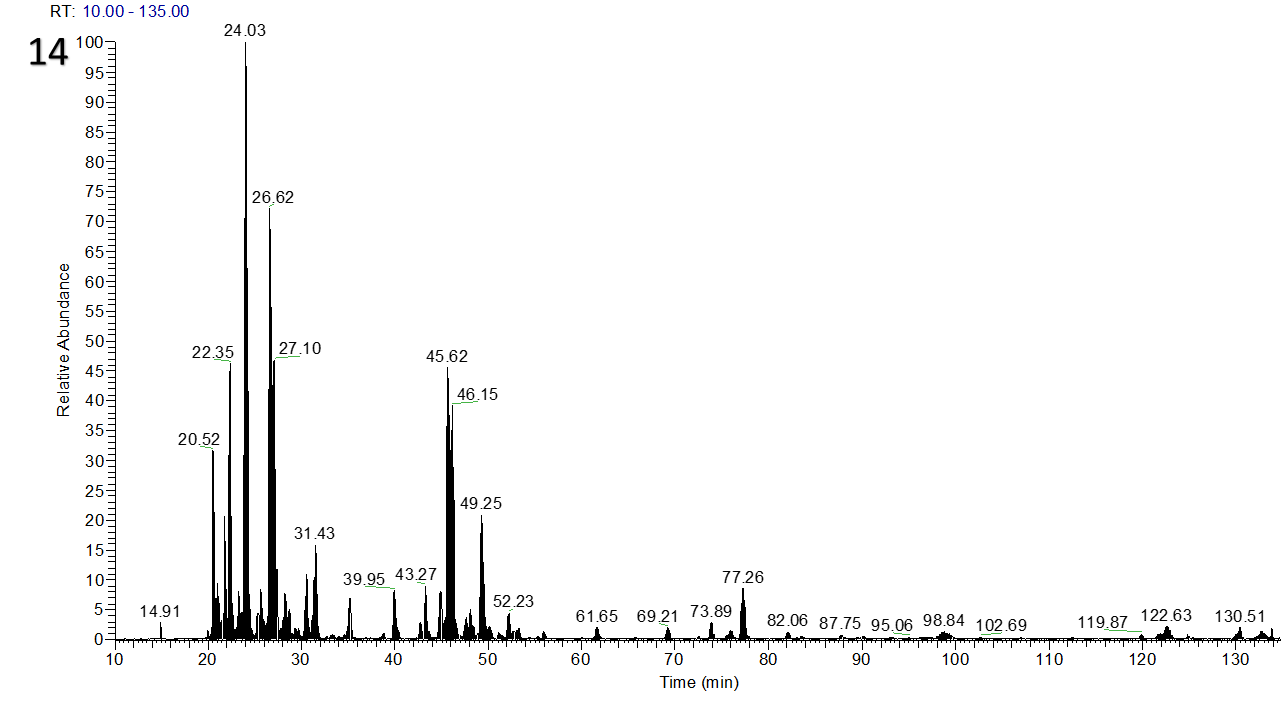


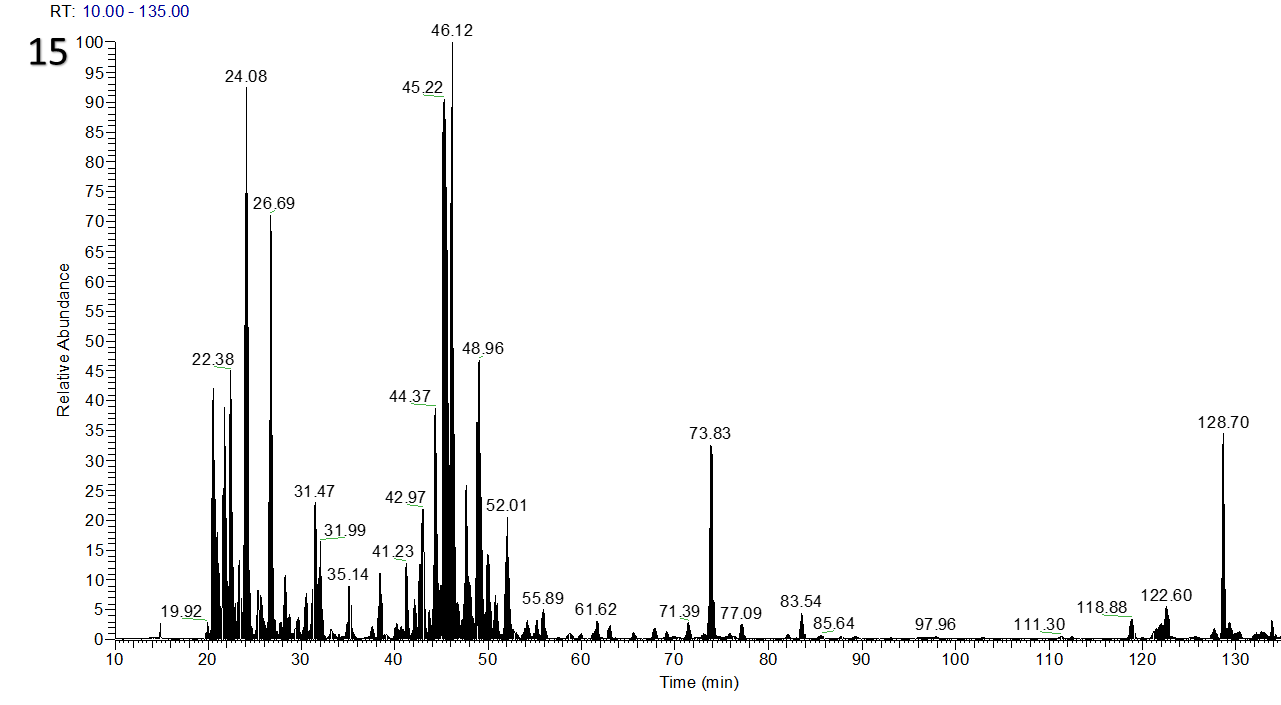


**Figure S3 (Cont.)**


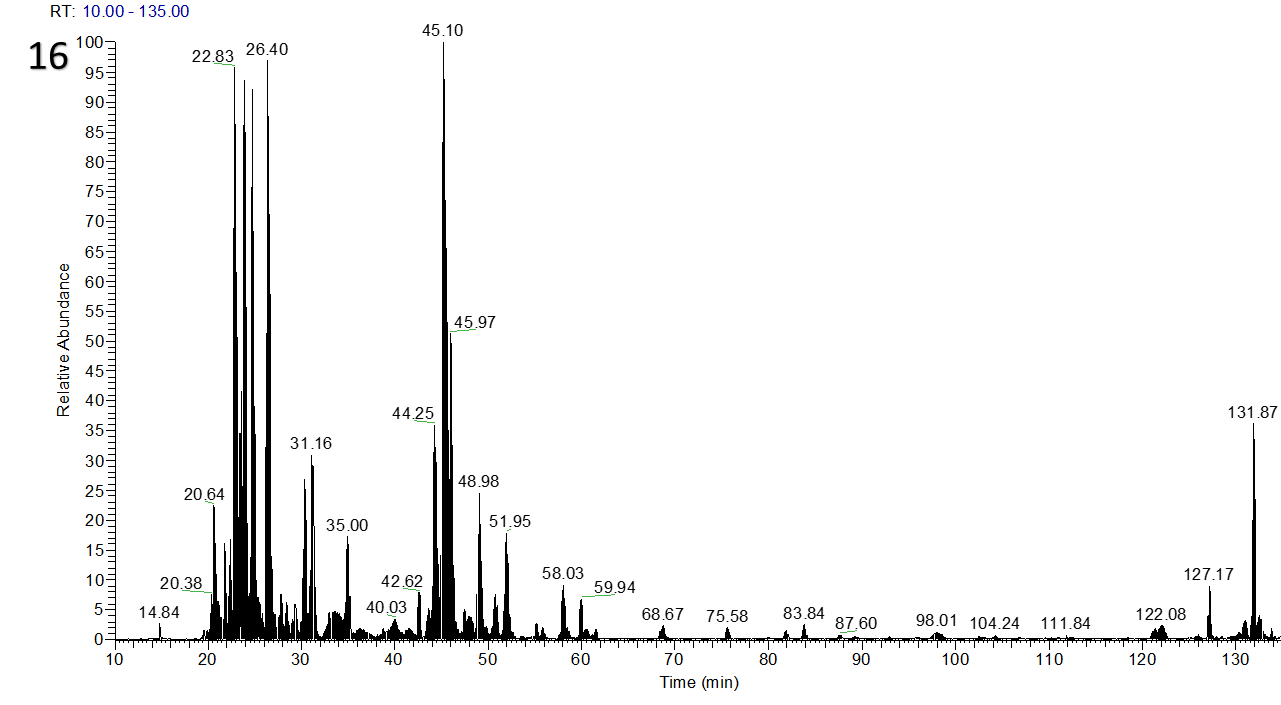


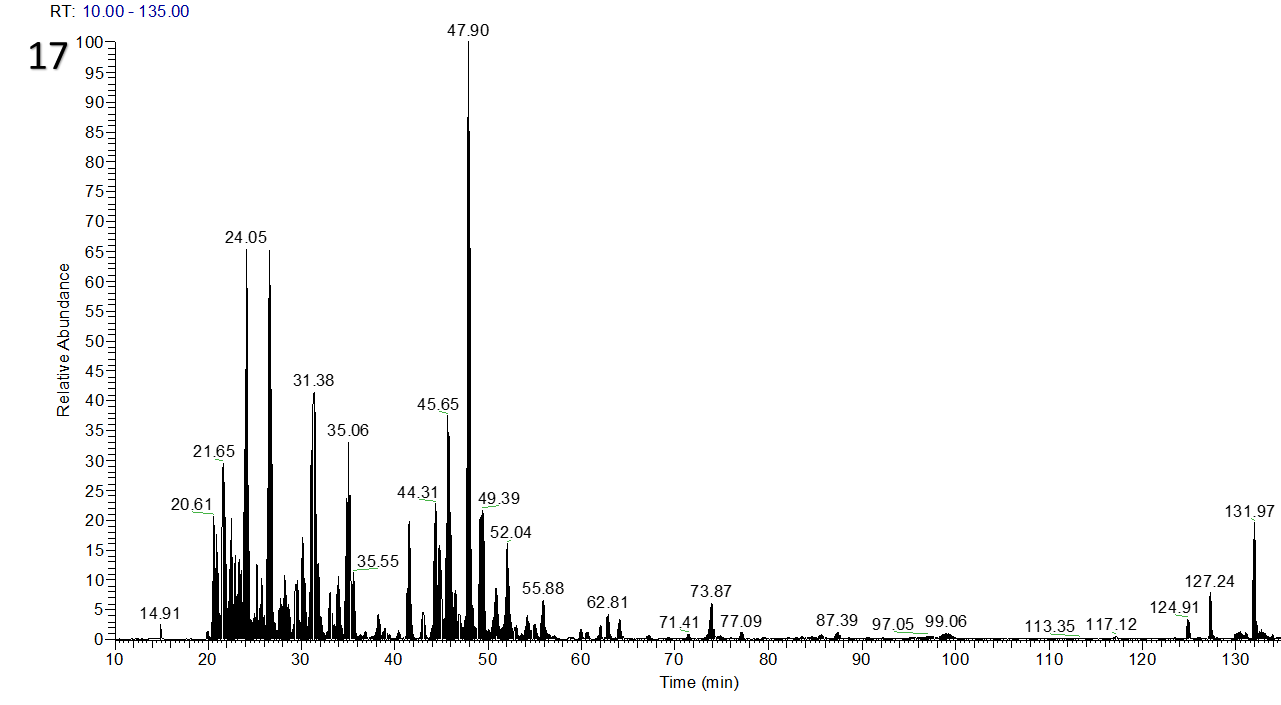


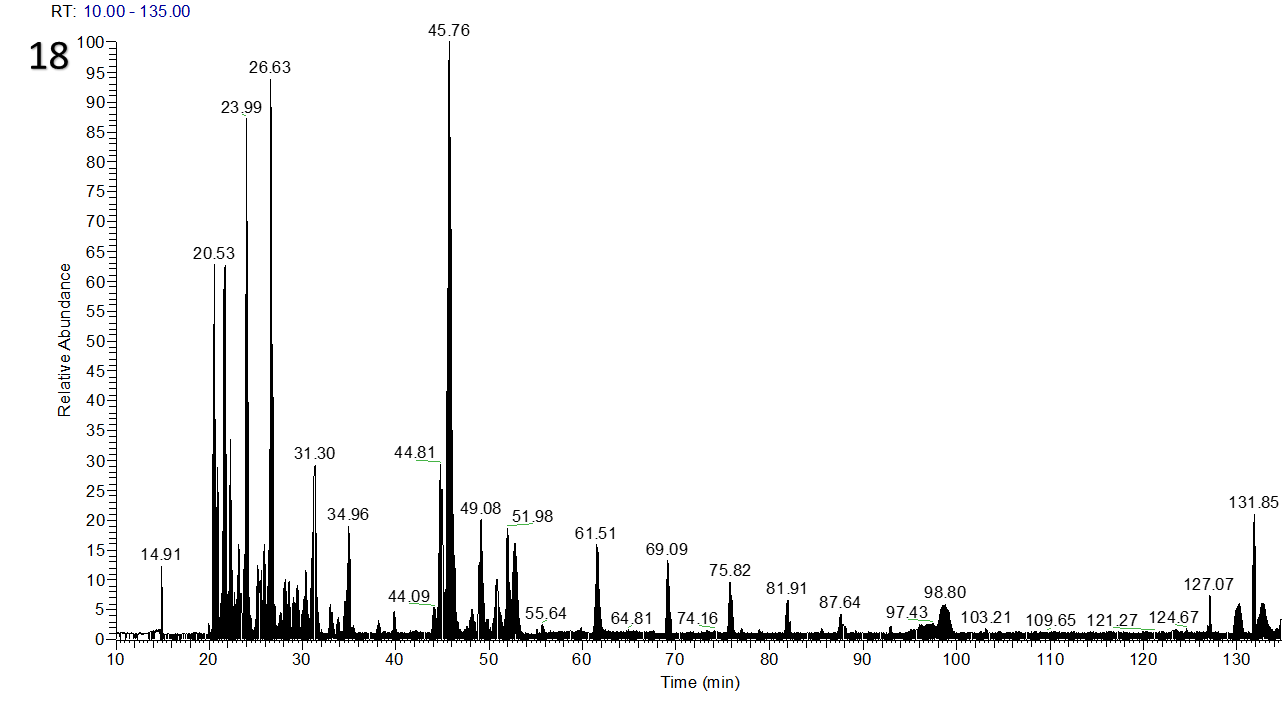


**Figure S3 (Cont.)**


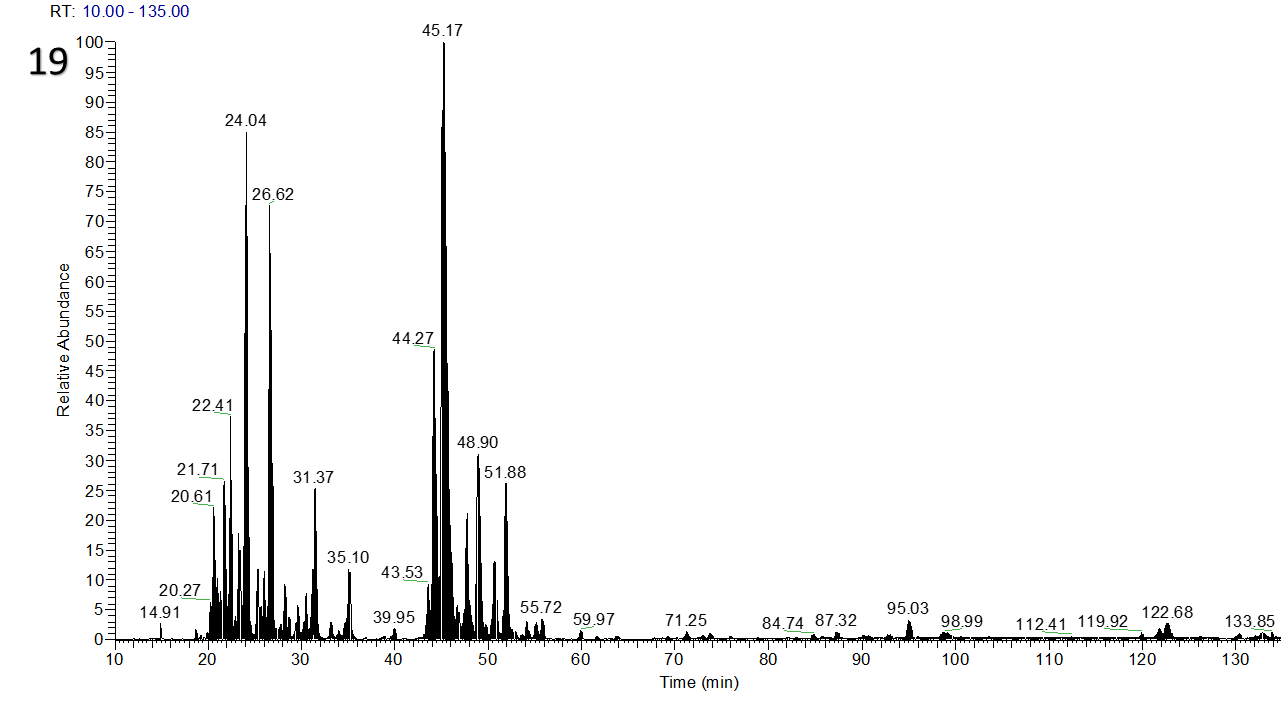


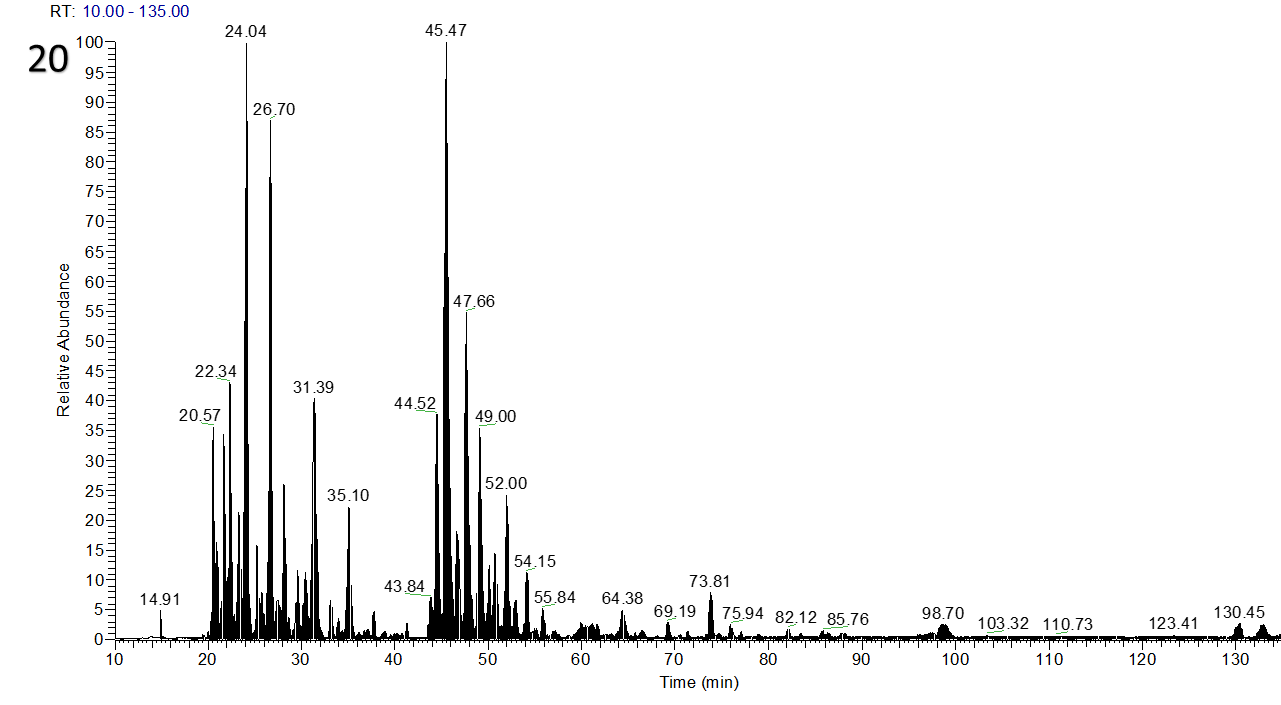


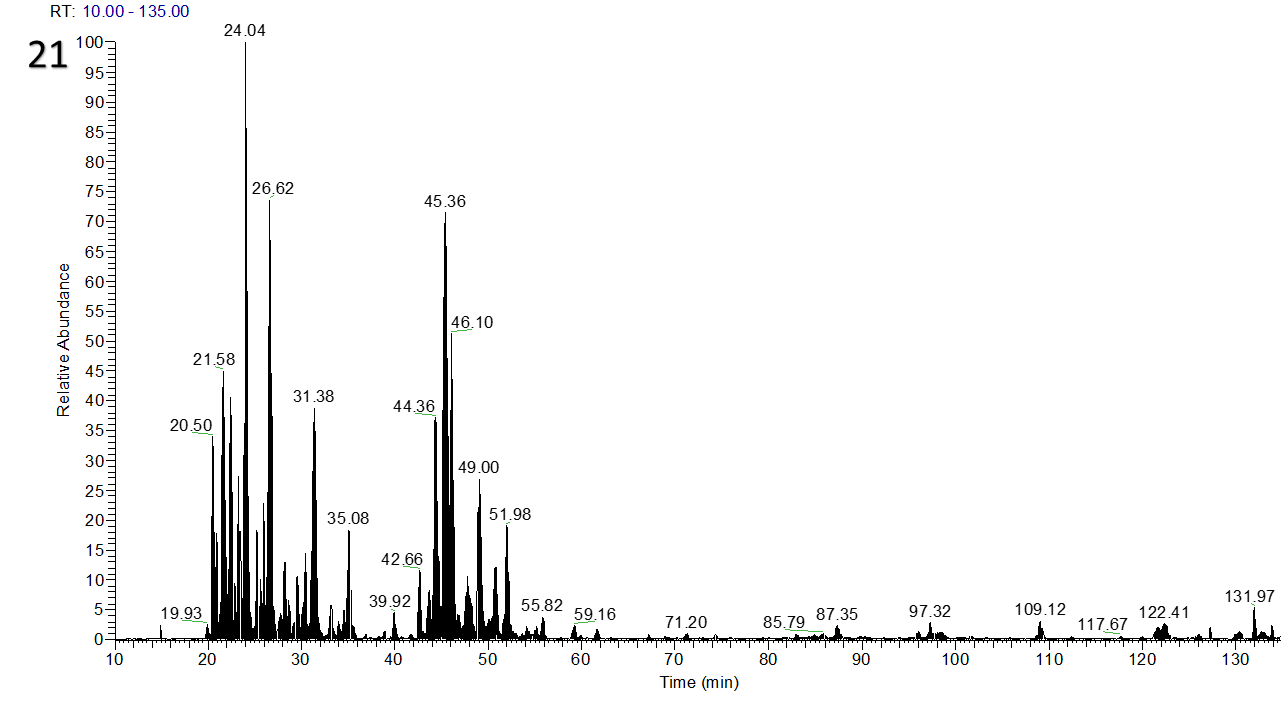


**Figure S3 (Cont.)**


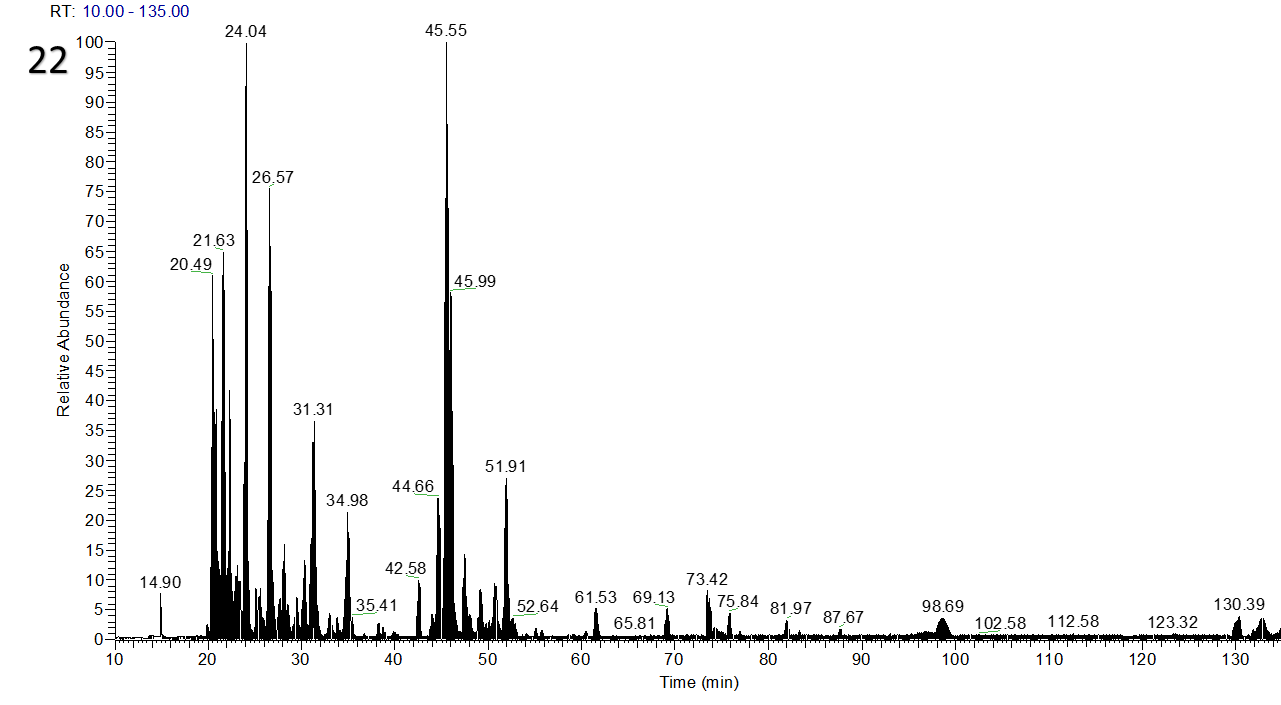


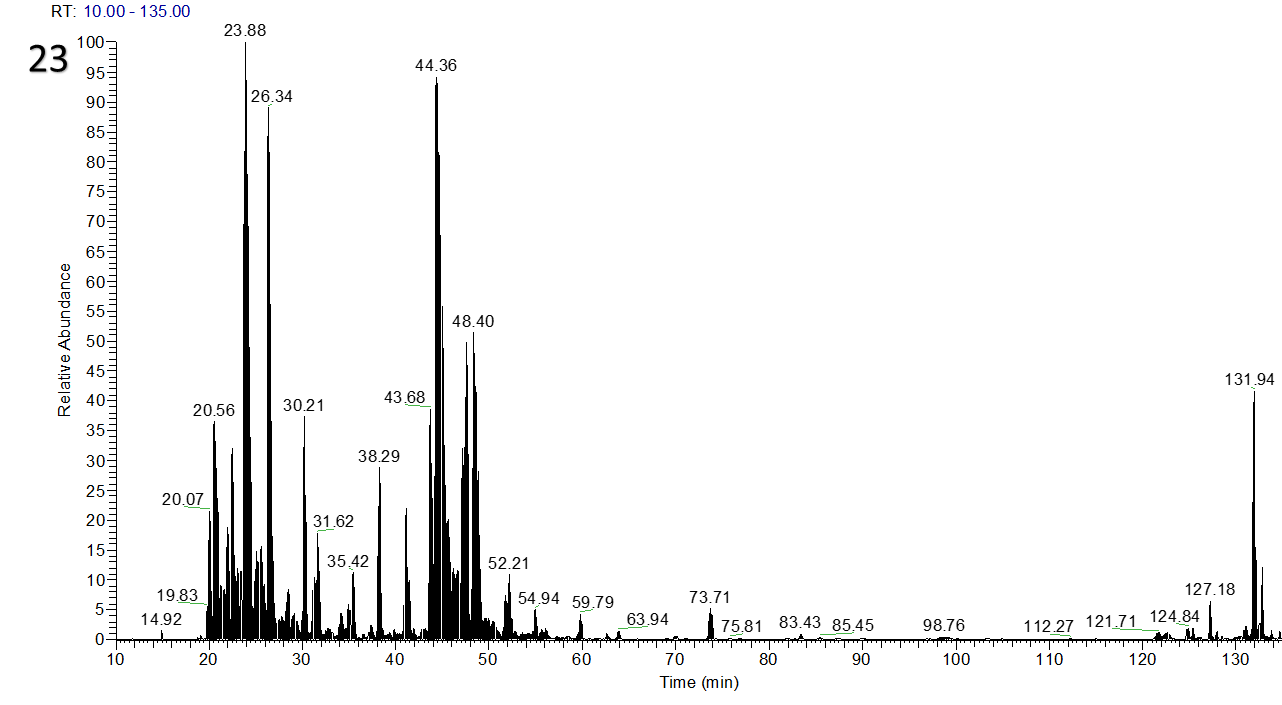


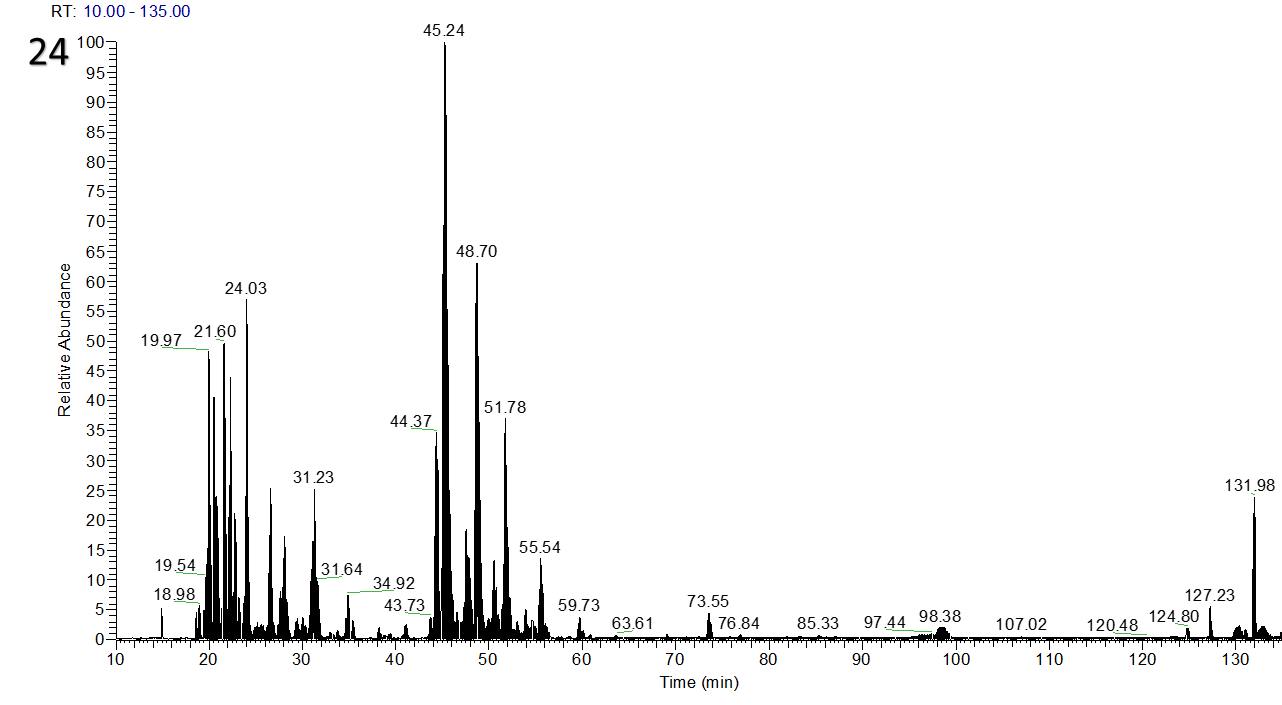


**Figure S3 (Cont.)**


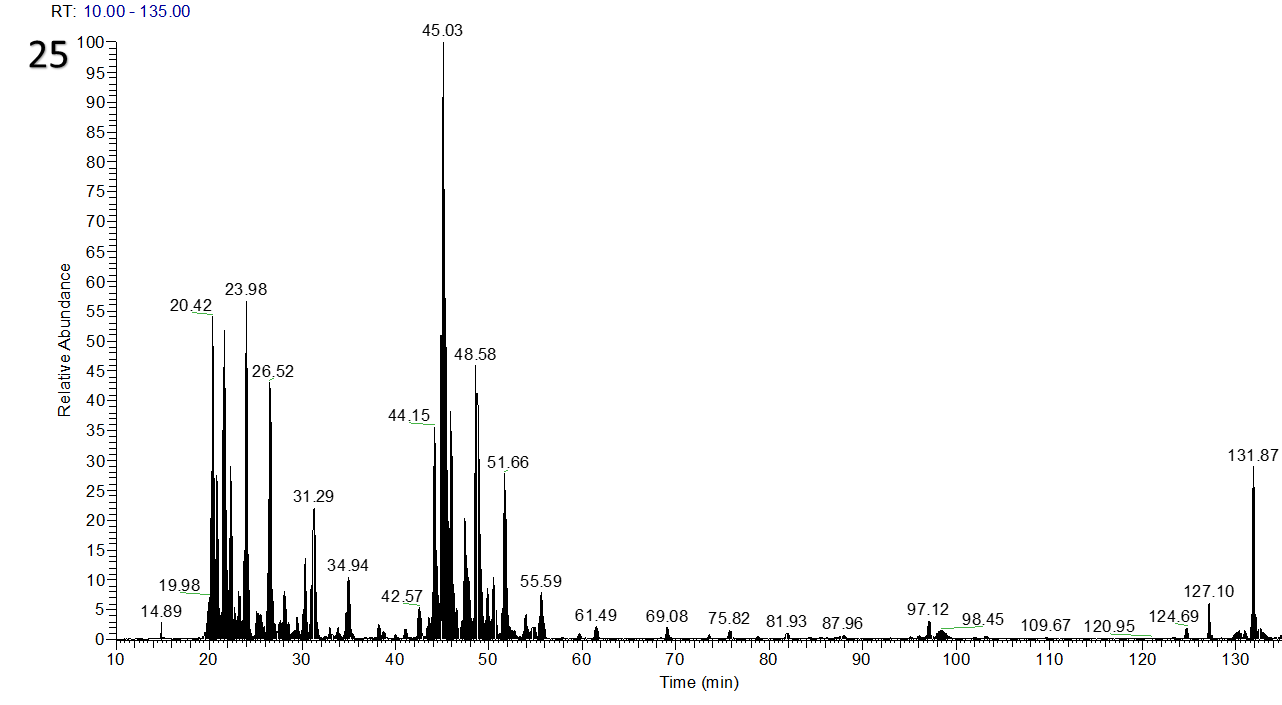


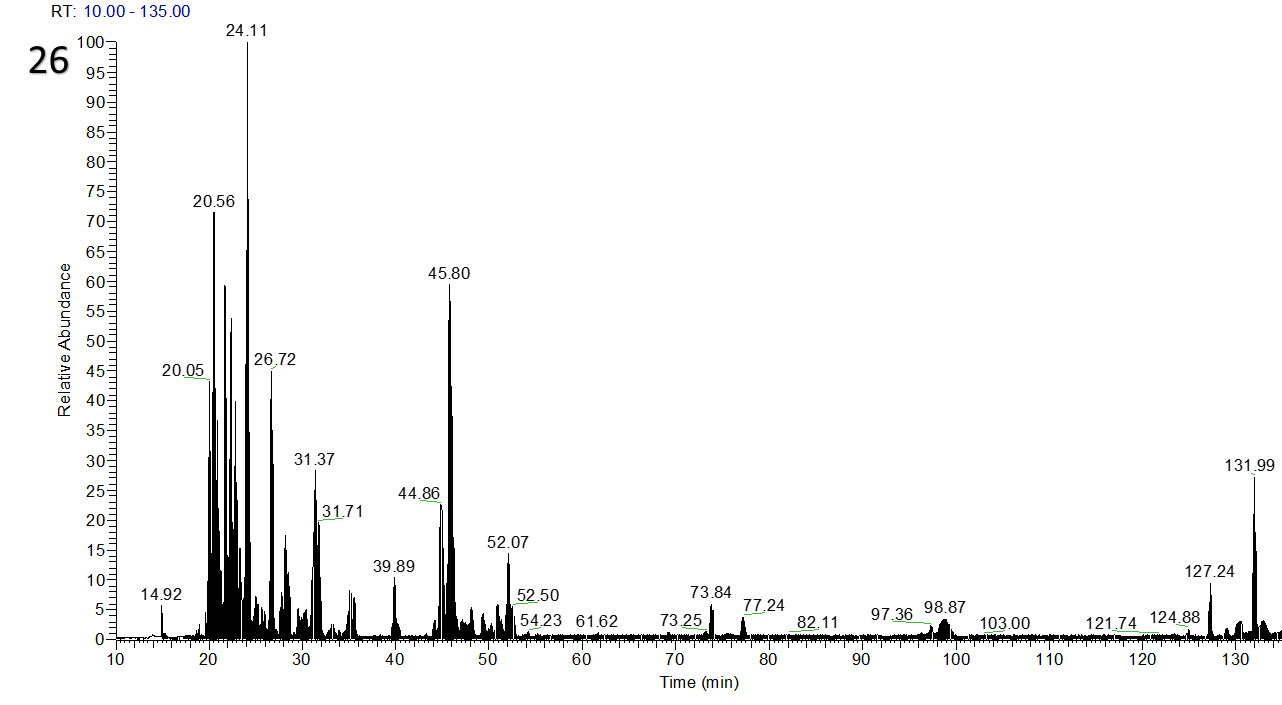

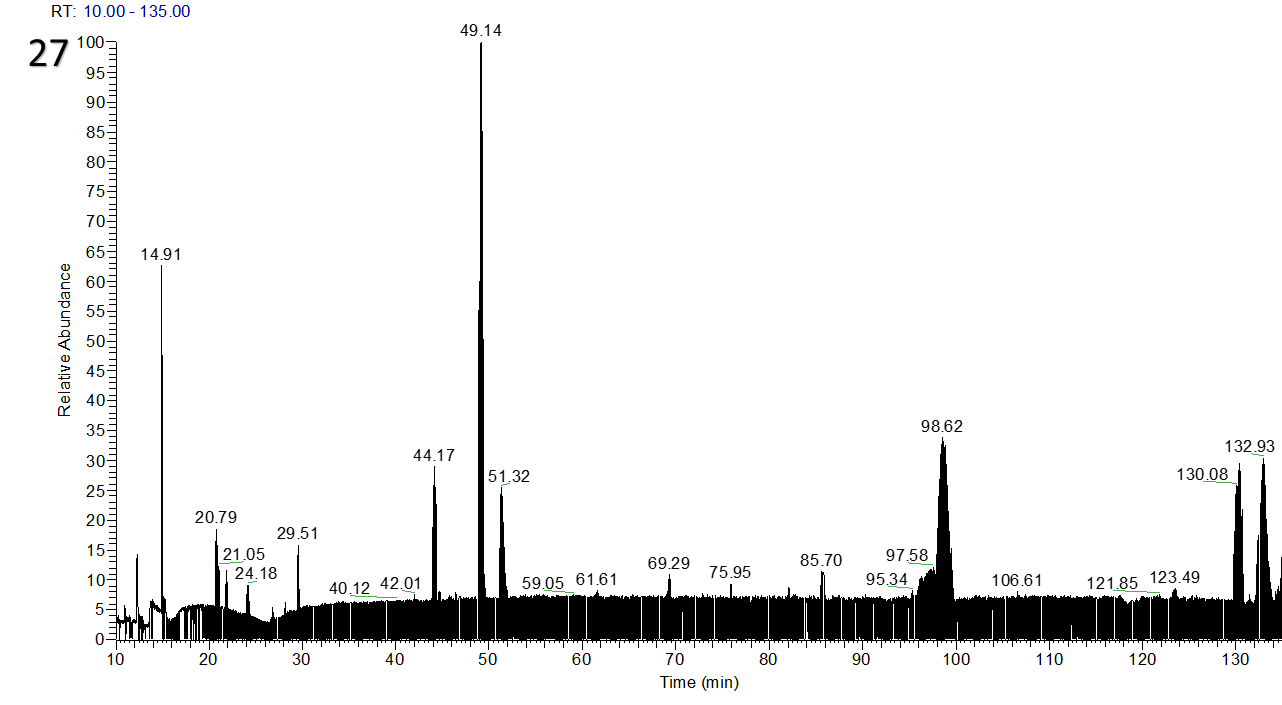


**Figure S3 (Cont.)**

**Features of the New Base Conopeptides in *Conus purpurascens***

Here we describe the main features of newly discovered conopeptides in the injected venom of *C. purpurascens*. We include the sequence of the precursor proteins from the transcriptome and highlight the corresponding signal and mature sequences. We also show the annotated MS/MS spectra. When possible, we compare the novel *C. purpurascens* sequences to known conopeptides, which can confer putative structural and functional characteristics to these newly described peptides.

**Linear**

**B2 linear** belongs to the B2 Superfamily. This family of linear peptides is expressed in other *Conus* species based on sequences deposited in NCBI. A similar sequence, differing in a single residue, is expressed by *C. ermineus* (Sequence ID: AXL95472) (1)*,* a close relative to *C. purpurascens*. This is the first evidence of these B2 linear peptides in injected venom.


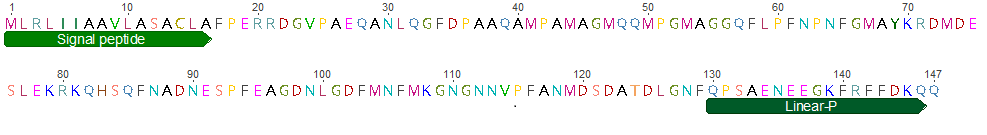


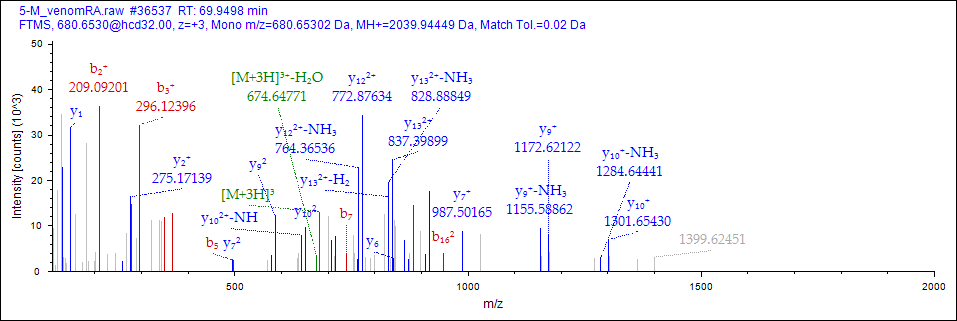


**Figure S4** Linear-P 1) full transcript with annotated signal sequence and mature peptide regions and 2) annotated MS/MS spectra

**One disulfide (C-C)**

**Ile-contryphan-P** belongs to the M superfamily. Ile-contryphan-P exhibits sequence homology to previously described leu-contryphan-P, except for a switch from L5 to W5. In general, contryphans are characterized by a conserved motif containing *D-*tryptophan or leucine and a single disulfide bond. Contryphans typically classify as part of the O2 superfamily based on their signal sequence, however Ile-contryphan-P does not follow this trend. The molecular target of contryphans remain unclear. Here, Ile-contryphan-P was identified in 25 of the 27 venom samples, suggesting it has an important function that needs to be discerned.


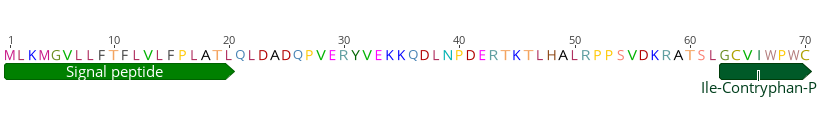


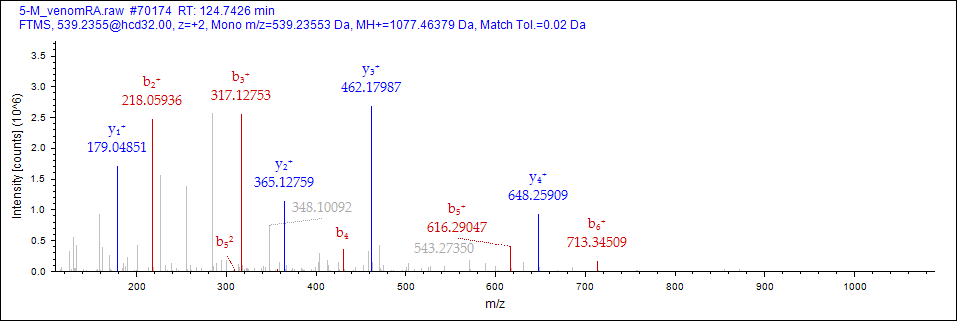


**Figure S5** Ile-Contryphan-P 1) full transcript with annotated signal sequence and mature peptide regions and 2) annotated MS/MS spectra

**Contryphan-P3** belongs to the M superfamily. Based on precursor analysis, these peptides form a new group of one disulfide peptides within the M superfamily (2). Contryphan-P3 is also expressed by *C. ermineus* (Sequence ID: AXL95407) (1), but this is the first instance in venom.


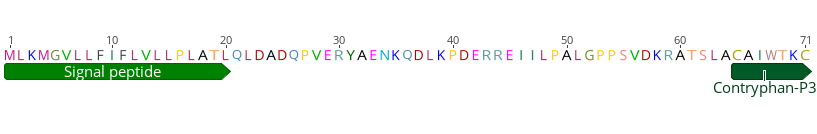


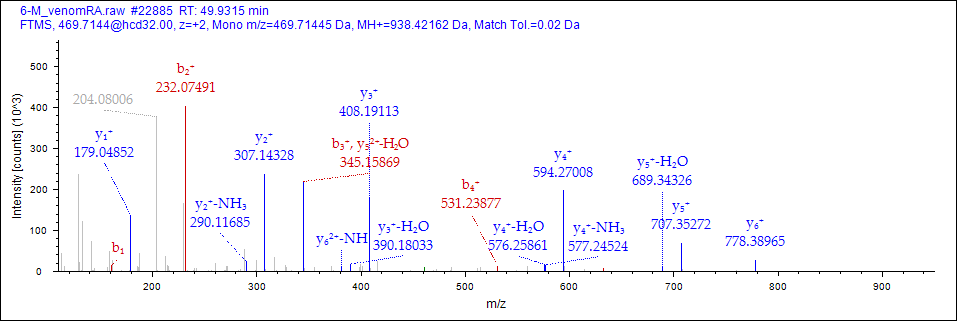


**Figure S6** Contryphan-P 1) full transcript with annotated signal sequence and mature peptide regions and 2) annotated MS/MS spectra

**Contryphan-P4** is not expressed in the venom duct, it was first discovered by *de novo* sequencing using PEAKS software (Unpublished data from our lab). Like Contryphan P3, the same sequence is found in *C. ermineus* venom duct transcriptome and belongs to the M superfamily (Sequence ID: AXL95569). Contryphan-P3 and P4 have different expression patterns and cluster into separate cabals. (Figure 3).


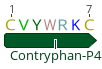


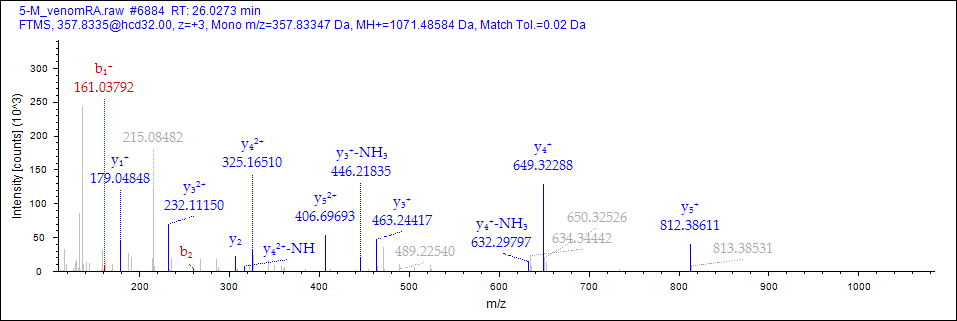


**Figure S7** Contryphan-P3 1) mature peptide sequence and 2) annotated MS/MS spectra

**Framework I (CC-C-C)**

**PID** belongs to superfamily A. The sequence of PID has been previously reported as a nucleic acid sequence from *C. ermineus*, E1.1 precursor (P03002, Conoserver). Here we provide the first evidence for the mature peptide in milked venom from *C. purpurascens*. The mature peptide exhibits homology to α-PIA (*C. purpurascens*) and to α-GID (*C. geographus,* P60274), suggesting PID will inhibit the nAChR (3,4).


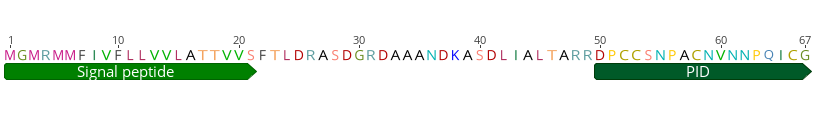


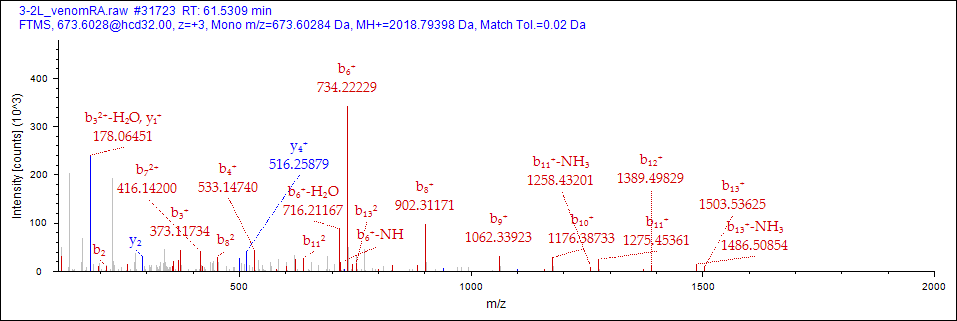


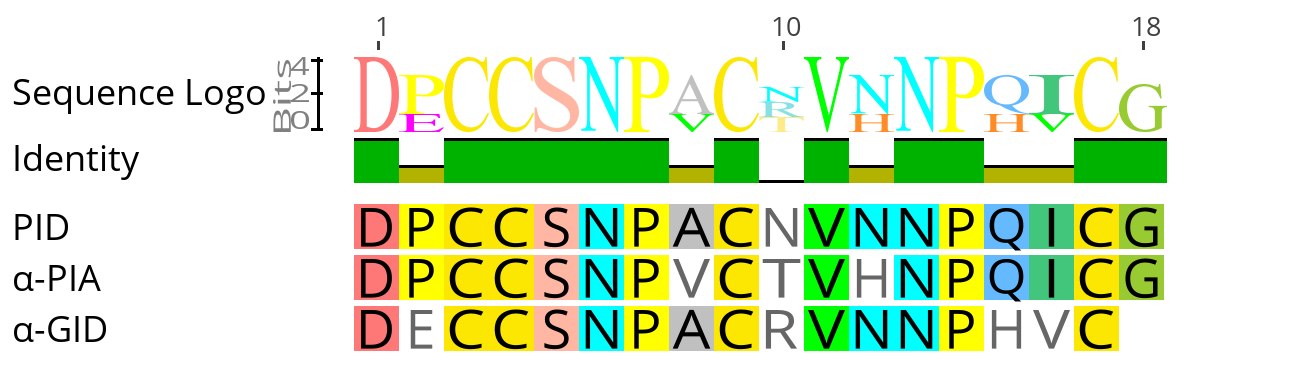


**Figure S8** PID 1) full transcript with annotated signal sequence and mature peptide regions, 2) annotated MS/MS spectra and 3) sequence alignment with α-PID and α-GID.

**PIE** belongs to superfamily A. Homology to α-conotoxin precursor Bt1.8 from *C. betulinus* (Sequence ID: A0A068B6Q6) and characterized α-GIC (Sequence ID: Q86RB2) that inhibits α3β2 receptors (5). Interestingly, MS/MS data from this study identified PIE with an extended N-terminal tail lacked by characterized α-conotoxins.

**
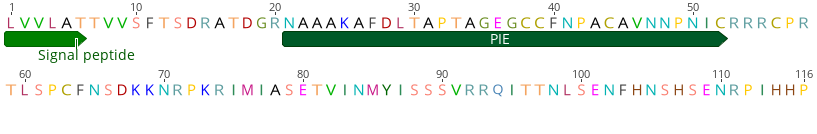
**

**
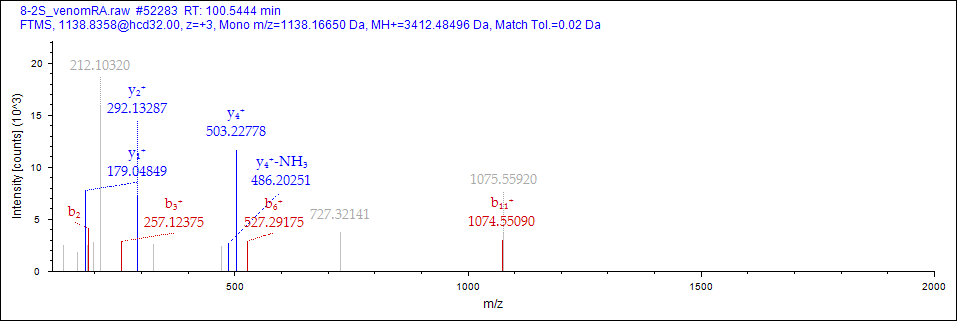
**

**
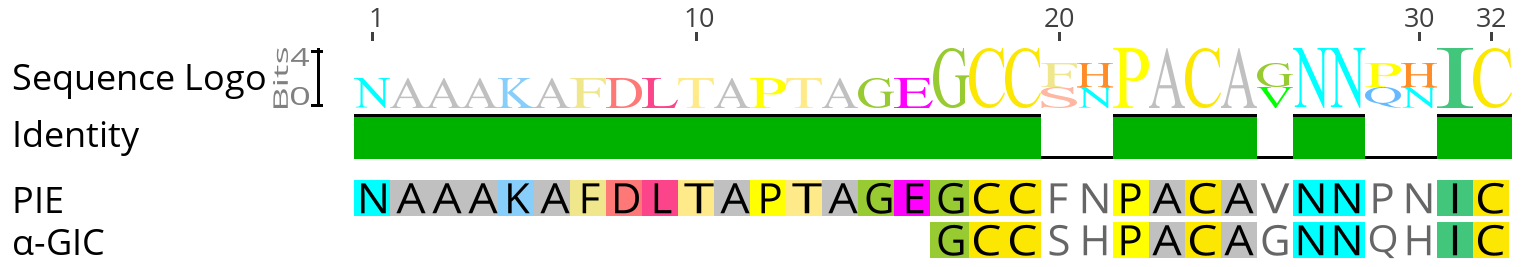
**

**Figure S9** PIE 1) full transcript with annotated signal sequence and mature peptide regions, 2) annotated MS/MS spectra, and 3) sequence alignment with α-GIC

**PIF** is a new framework I conotoxin that was first sequenced *de novo* using PEAKS (Unpublished data). Because it was sequenced *de novo*, the superfamily is unable to be assigned. The mature peptide shows homology to EIIA from *C. ermineus* (Sequence ID: D4HRK4) which inhibits muscle subtype nAChRs (6). PIF is also similar to α-PIB, a muscle subtype-selective conotoxin from *C. purpurascens* (7).


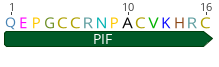


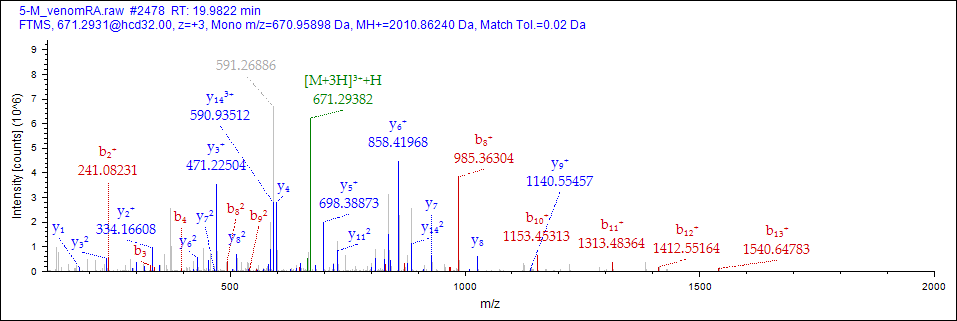


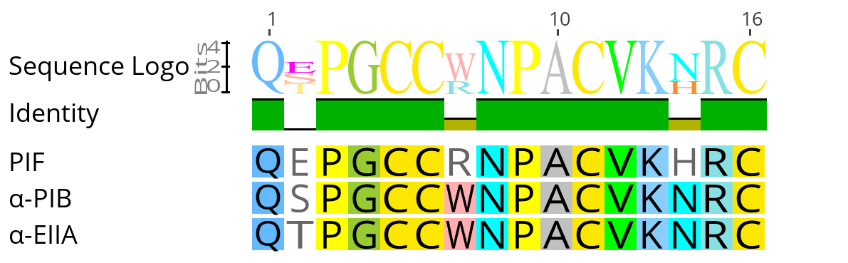


**Figure S10** PIF 1) mature peptide sequence, 2) annotated MS/MS spectra, and 3) sequence alignment with α-PIF and α-EIIA

**PIG** is a framework I conotoxin that was sequenced *de novo* using PEAKS software (Unpublished data). We lack signal sequence information, however the mature peptide sequence shows high homology (82%) to α-PIA that inhibits α6 nAChRs (3).

**
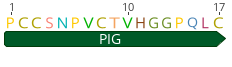
**

**
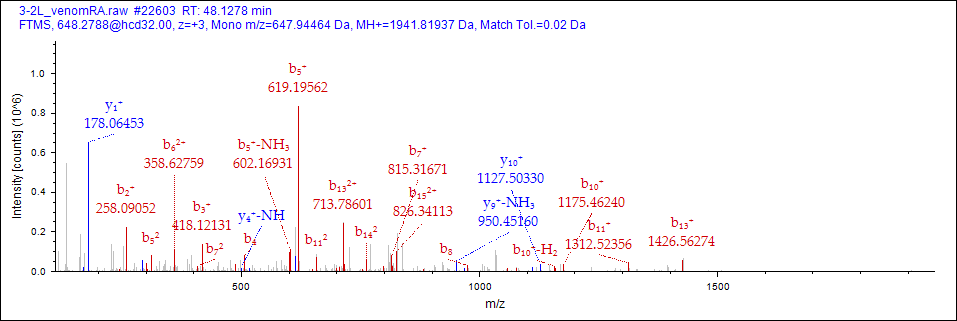
**

**
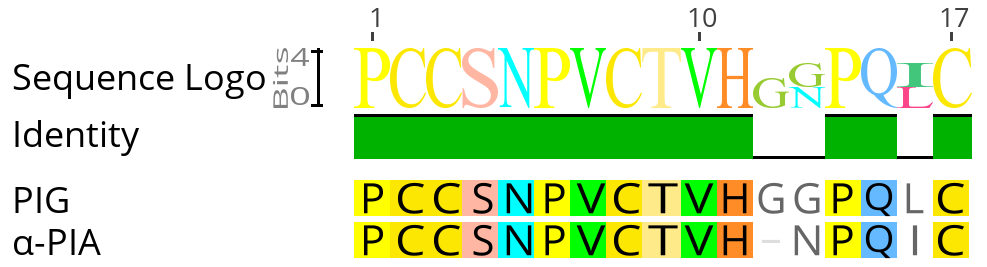
**

**Figure S11** PIG 1) mature peptide sequence, 2) annotated MS/MS spectra, and 3) alignment with α-PIA

**Framework II (CCC-C-C-C)**

**PIIA** exhibits cysteine framework II and belongs to the O3 Superfamily. A very similar sequence is found in the *C. ermineus* venom duct transcriptome (Sequence ID: AXL95373) (1). There are no similar characterized conotoxins from which we can infer activity. It is important to note that there is an extra cystine pair upstream from the identified N-terminus, and it is possible that the mature peptide identified through our approaches was in fact a truncated version. If this extra cysteine pair is part of the mature peptide, it would form a novel four-disulfide peptide with a new cysteine framework (C-C-CCC-C-C-C).


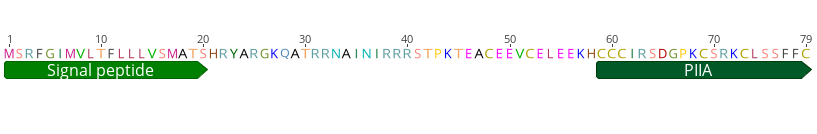


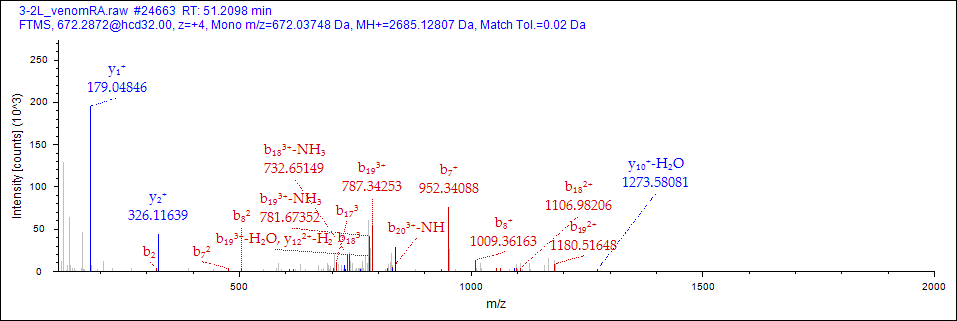


**Figure S12** PIIA 1) full transcript with annotated signal sequence and mature peptide regions and 2) annotated MS/MS spectra

**Framework III (CC-C-C-CC)**

**PIIIG** belongs to the M superfamily that clusters with the motor cabal. It is an M1 mini-M with loops sizes 4/2/1 according to the number of residues between cysteine residues (8). The mature peptide has very little sequence homology to other mini-Ms. The pharmacology of the mini-Ms remains unknown, despite their prevalence across *Conus* species (9).

**
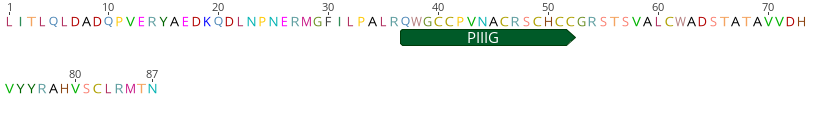
**

**
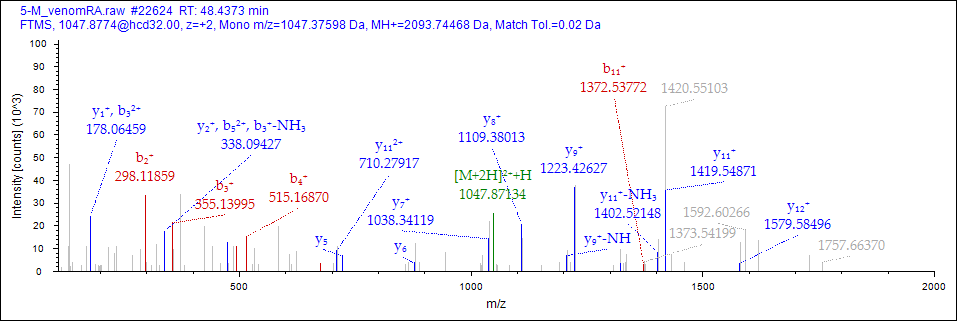
**

**Figure S13** PIIIG 1) full transcript with annotated mature peptide region and 2) annotated MS/MS spectra

**PIIIH** is an M superfamily conotoxin that clusters with the motor cabal. It is an M1 mini-M with loop sizes 4/5/1. PIIIH clusters with motor cabal toxins. It exhibits sequence homology to a peptide expressed in the venom duct of fish-hunting cone snail *C. magus* (Sequence ID: QFQ61044), however there is no evidence of this peptide in the injected venom (10).


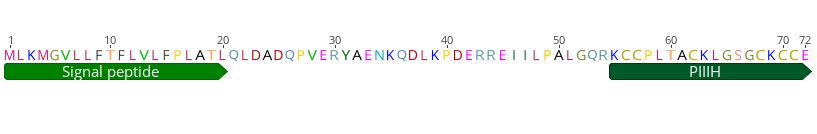


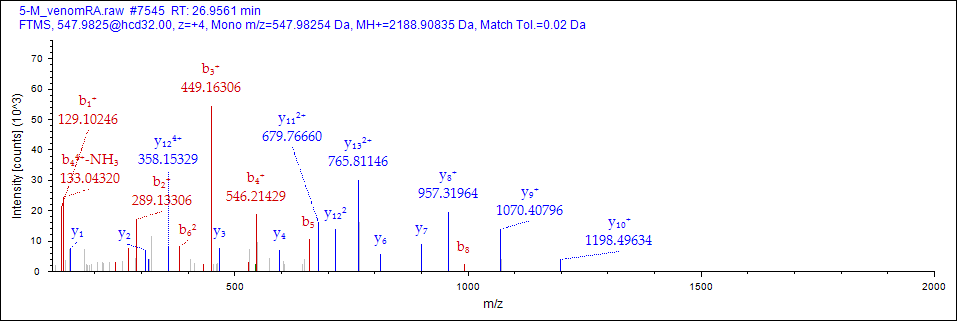


**Figure S14** PIIIH 1) full transcript with annotated signal sequence and mature peptide regions and 2) annotated MS/MS spectra

**PIIII** is a mini-M (M2) from the M Superfamily. It is the only one of the 3 identified venom mini-Ms that clustered with lightning-strike cabal peptides (Figure 3). Similar transcripts are found in *Turriconus* species (ATF27414, ATF27651) (11), and in *C. regius* (P85021) (9). These are all worm-hunting species, supporting previous evidence that *C. purpurascens* may employ a mixed-mode feeding strategy (Unpublished data).

**
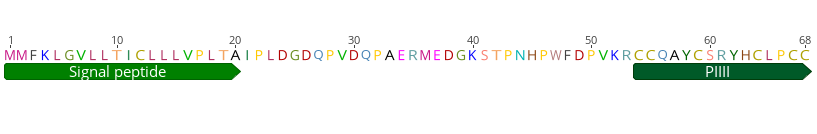
**

**
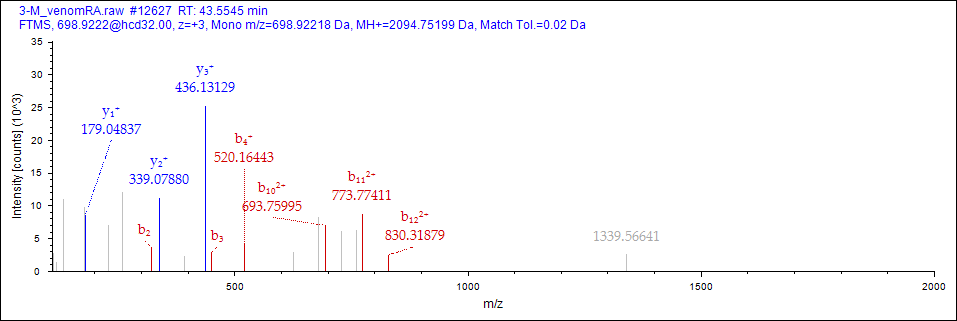
**

**Figure S15** PIIII 1) full transcript with annotated signal sequence and mature peptide regions and 2) annotated MS/MS spectra

**Framework IV (CC-C-C-C)**

**PIVH** belongs to the A superfamily. Its expression in the milked venom clusters closely with κ-PIVF and other lightning strike cabal toxins. PIVH shares ~60% homology with κ-PIVE and κ-PIVF, strongly indicating it also targets the potassium channel (12). PIVH has two adjacent glutamic acid residues, E8 and E9, that can be modified as carboxyglutamate. Both versions of the peptide were identified in the venom. We have included the MS/MS spectra (Figure S16a) and y and b ion series (Table S38) for the carboxy-E8 toxiform.


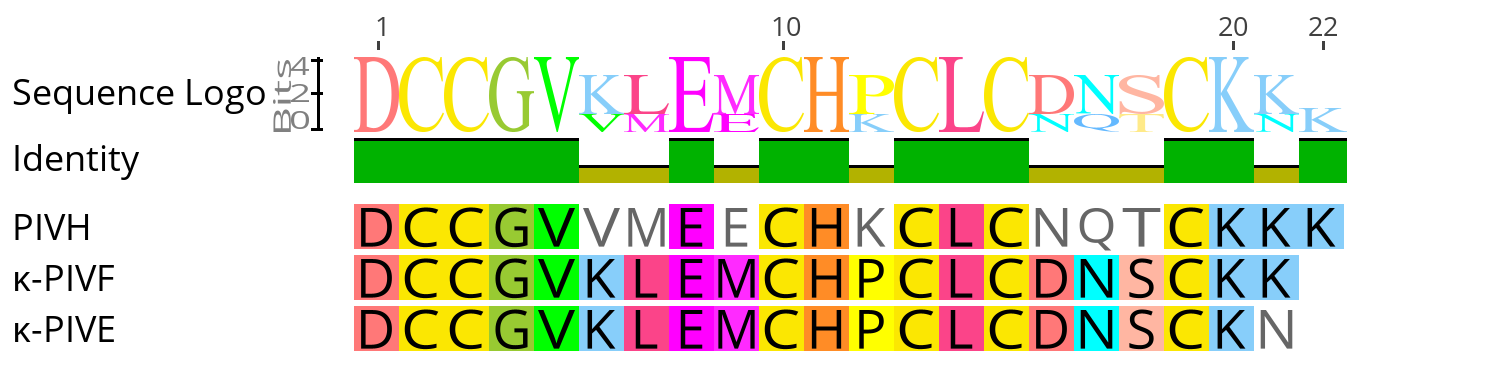

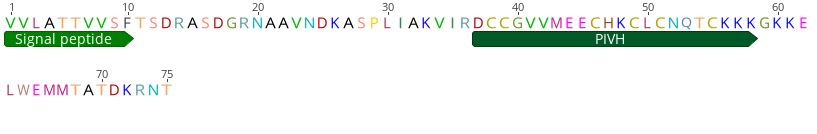

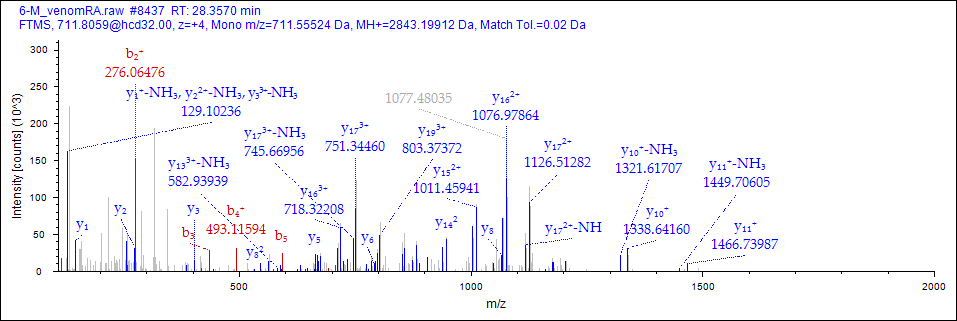


**Figure S16** PIVH 1) full transcript with annotated signal sequence and mature peptide regions 2) annotated MS/MS spectra, and 3) alignment with κ-PIVF and κ-PIVE


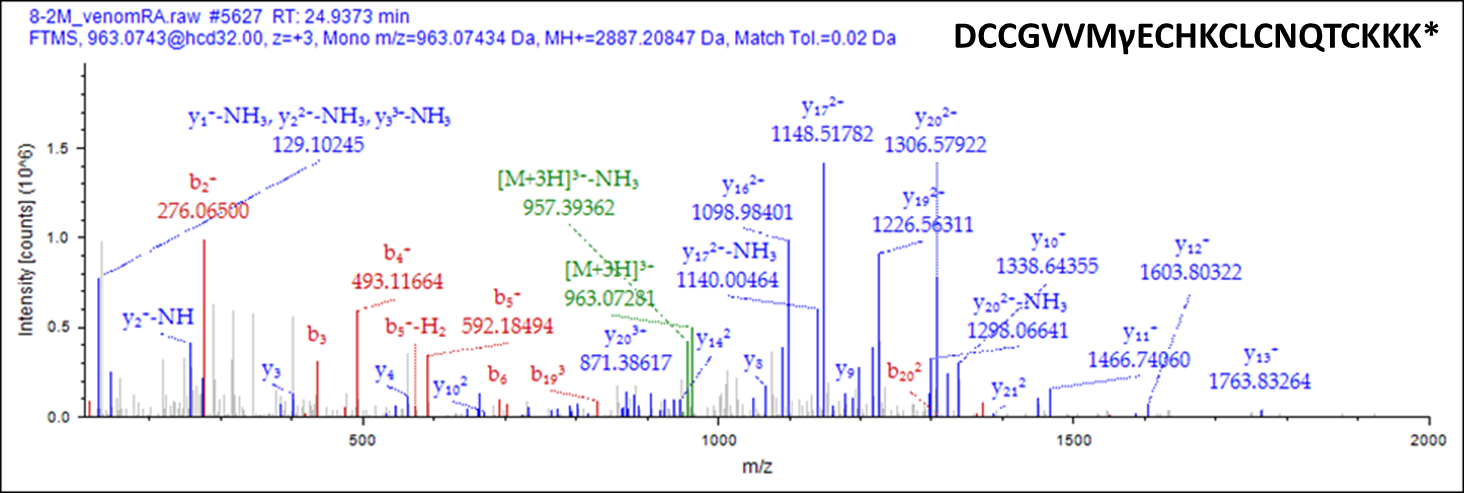
**Figure S16a-** MS/MS spectra for PIVH peptide with carboxyglutamate at position E8 and amidation of the c-terminal (DCCGVVMγECHKCLCNQTCKKK*).

| **#1** | **b⁺** | **b²⁺** | **b³⁺** | **Seq.** | **y⁺** | **y²⁺** | **y³⁺** | **#2** |
| --- | --- | --- | --- | --- | --- | --- | --- | --- |
| 1 | 116.03422 | 58.52075 | 39.34959 | D |  |  |  | 22 |
| 2 | 276.06487 | 138.53607 | 92.69314 | C-Carbamidomethyl | 2772.16352 | 1386.58540 | 924.72602 | 21 |
| 3 | 436.09552 | 218.55140 | 146.03669 | C-Carbamidomethyl | 2612.13287 | 1306.57007 | 871.38247 | 20 |
| 4 | 493.11698 | 247.06213 | 165.04384 | G | 2452.10222 | 1226.55475 | 818.03892 | 19 |
| 5 | 592.18539 | 296.59634 | 198.06665 | V | 2395.08076 | 1198.04402 | 799.03177 | 18 |
| 6 | 691.25381 | 346.13054 | 231.08945 | V | 2296.01234 | 1148.50981 | 766.00897 | 17 |
| 7 | 822.29429 | 411.65078 | 274.76962 | M | 2196.94393 | 1098.97560 | 732.98616 | 16 |
| 8 | 995.32671 | 498.16700 | 332.44709 | E-Carboxy | 2065.90344 | 1033.45536 | 689.30600 | 15 |
| 9 | 1124.36931 | 562.68829 | 375.46129 | E | 1892.87102 | 946.93915 | 631.62853 | 14 |
| 10 | 1284.39996 | 642.70362 | 428.80484 | C-Carbamidomethyl | 1763.82843 | 882.41785 | 588.61433 | 13 |
| 11 | 1421.45887 | 711.23307 | 474.49114 | H | 1603.79778 | 802.40253 | 535.27078 | 12 |
| 12 | 1549.55383 | 775.28055 | 517.18946 | K | 1466.73887 | 733.87307 | 489.58447 | 11 |
| 13 | 1709.58448 | 855.29588 | 570.53301 | C-Carbamidomethyl | 1338.64391 | 669.82559 | 446.88615 | 10 |
| 14 | 1822.66854 | 911.83791 | 608.22770 | L | 1178.61326 | 589.81027 | 393.54260 | 9 |
| 15 | 1982.69919 | 991.85323 | 661.57125 | C-Carbamidomethyl | 1065.52919 | 533.26824 | 355.84792 | 8 |
| 16 | 2096.74212 | 1048.87470 | 699.58556 | N | 905.49855 | 453.25291 | 302.50437 | 7 |
| 17 | 2224.80070 | 1112.90399 | 742.27175 | Q | 791.45562 | 396.23145 | 264.49006 | 6 |
| 18 | 2325.84837 | 1163.42783 | 775.95431 | T | 663.39704 | 332.20216 | 221.80386 | 5 |
| 19 | 2485.87902 | 1243.44315 | 829.29786 | C-Carbamidomethyl | 562.34936 | 281.67832 | 188.12131 | 4 |
| 20 | 2613.97399 | 1307.49063 | 871.99618 | K | 402.31871 | 201.66300 | 134.77776 | 3 |
| 21 | 2742.06895 | 1371.53811 | 914.69450 | K | 274.22375 | 137.61551 | 92.07943 | 2 |
| 22 |  |  |  | K-Amidated | 146.12879 | 73.56803 | 49.38111 | 1 |

**Table S38-** Theoretical y- and b- ion series for PIVH peptide with carboxyglutamate at position E8 and amidation of the c-terminal (DCCGVVMγECHKCLCNQTCKKK*). Highlighted y- ions (blue) and b- ions (red) were matched to the MS/MS spectra (Figure S16a).

**Framework V (CC-CC)**

**PVB** is a T Superfamily conotoxin expressed in both transcriptomes. Its expression in the milked venom clustered closely with other motor cabal peptides (Figure 3). PVB is the second T Superfamily peptide identified from *C. purpurascens* venom but shows limited sequence homology to PVA aside from the conserved cysteine framework (Table 1). The same sequence is found in the transcriptome of *C. ermineus* (Sequence ID: AXL95476) (1). The pharmacology of T superfamily conotoxins is not well defined, however two framework V conotoxins from the T Superfamily are known to target the somatostatin-3 receptor (13).


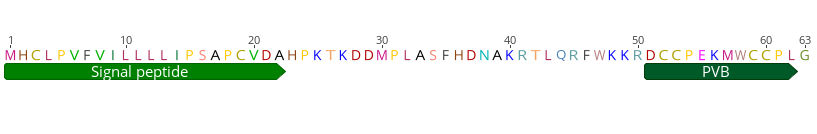


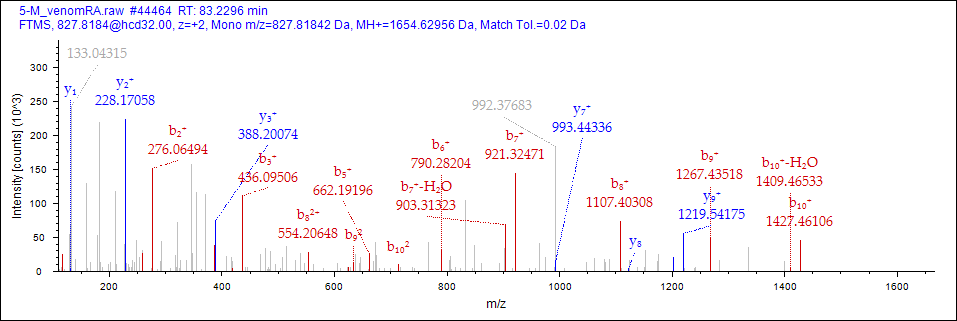


**Figure S17** PVB 1) full transcript with annotated signal sequence and mature peptide regions and 2) annotated MS/MS spectra

**Framework VI/VII (C-C-CC-C-C)**


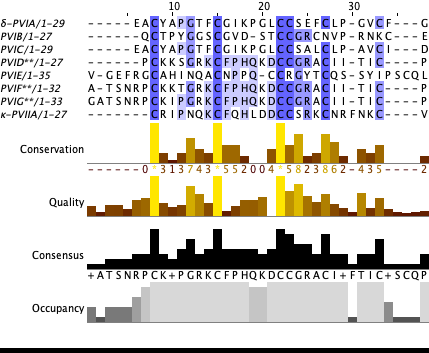


**PVIB** is an O1 Superfamily conotoxin. PVIB was identified in 21 of the 27 venom samples (Figure 2), suggesting it plays an important role in prey capture. It falls within cluster 1 (lightning-strike cabal) and is expressed in the venom of all 7 specimen that form this cluster (Figure 3). PVIB has high homology to a sequence from the venom duct transcriptome of *C. ermineus* (Sequence ID: AXL95467) (1).


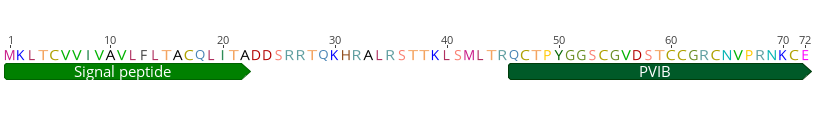


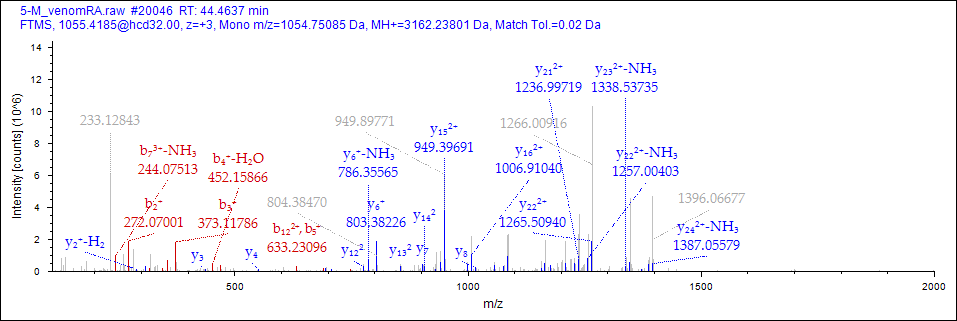


**Figure S18** PVIB 1) full transcript with annotated signal sequence and mature peptide regions and 2) annotated MS/MS spectra

**PVIC** belongs to the O1 Superfamily. It shows high homology (85%) to δ-PVIA, and the two share similar patterns of expression in the venom (Figure 3). Its sequence is also similar to δ-EVIB (Sequence ID: P69752). For this reason, it is likely PVIC will also target sodium channels (14) as part of the lightning-strike cabal.


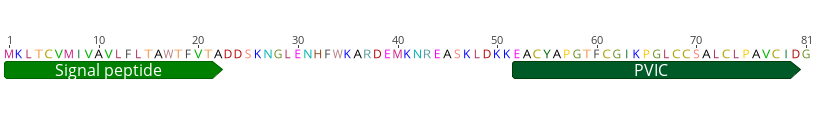


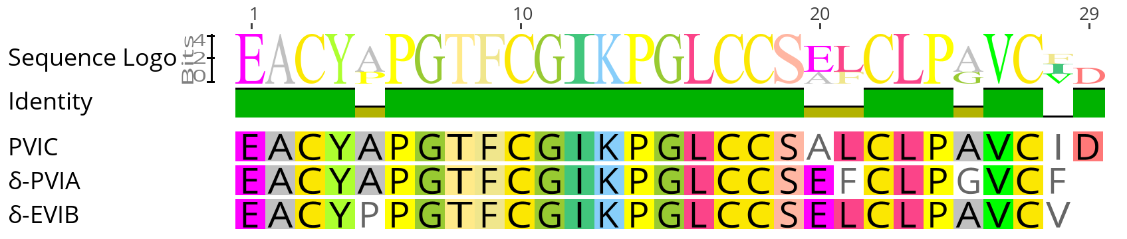


**Figure S20** PVIC A) full transcript with annotated signal sequence and mature peptide regions and B) alignment with δ-PVIA and δ-EVIB

**PVID** belongs to the O1 Superfamily. It was previously reported as a nucleic acid sequence, P2b (Sequence ID: AAQ05866) (15), but this is the first time reported in the venom. It clusters within the lightning strike cabal (Figure 3). It closely resembles *C. purpurascens* nucleic acid sequence p2a (Sequence ID: AAQ05865), and new peptides PVIF (p2c, AAQ05867), and PVIG (Figure S21) (15). It does not share homology to any peptides with known activity. It does not share significant homology to any peptides with known activity, however it has the same cysteine framework and minimal homology to κ-PVIIA (Figure S21).


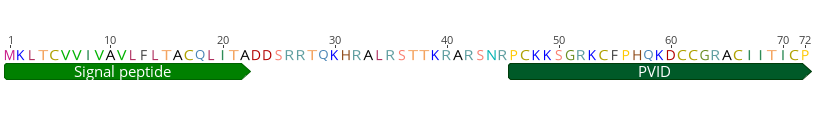


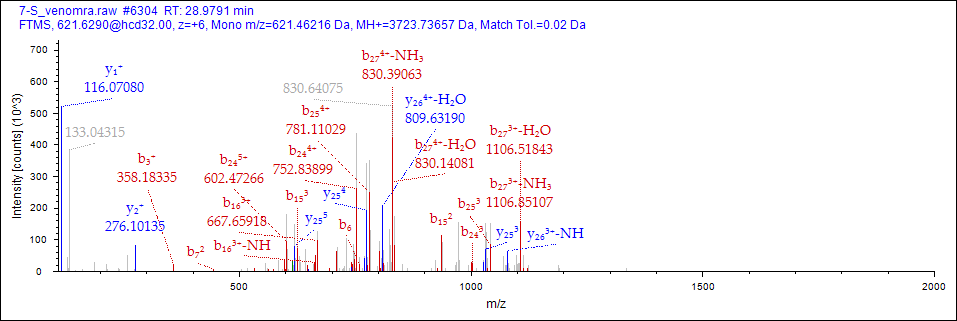


**Figure S21** PVID 1) full transcript with annotated signal sequence and mature peptide regions and 2) annotated MS/MS spectra

**PVIE** belongs to the O1 Superfamily. The mature peptide has little sequence homology to any characterized conotoxin, but has high homology to a nucleic acid sequence from *C. ermineus* (Sequence ID: AXL95668) (1). Interestingly, it is the only framework VI/ O1 Superfamily toxin that is expressed within cluster 2 (motor cabal) (Figure 3).


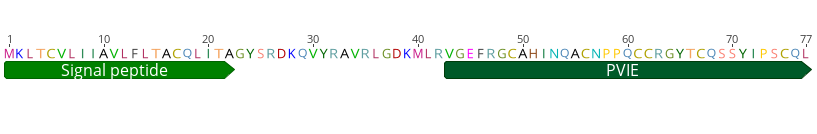


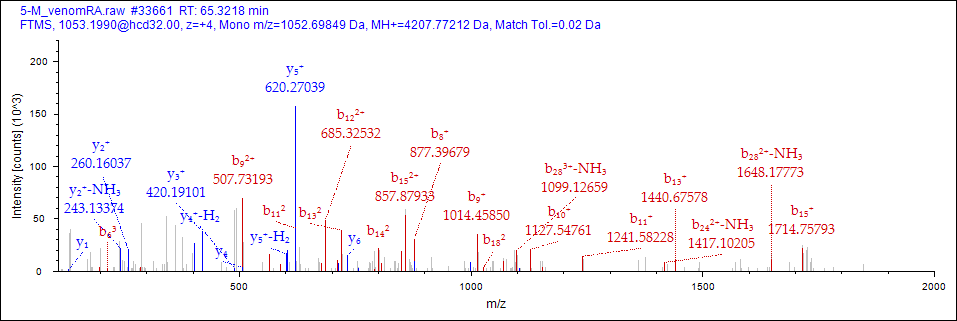


**Figure S22** PVIE 1) full transcript with annotated signal sequence and mature peptide regions and 2) annotated MS/MS spectra

**PVIF** belongs to the O1 Superfamily. It was previously reported as a nucleic acid sequence, P2c (Sequence ID: AAQ05867) (15), but this is the first time reported in the venom. PVIF was only identified in the venom of one specimen (Figure 2). It closely resembles *C. purpurascens* nucleic acid sequences p2a (Sequence ID: AAQ05865), PVID (p2b, AAQ05866) and PVIG (Figure S21) (15). It clusters within the lightning strike cabal (Figure 3). It does not share significant homology to any peptides with known activity, but shares minimal homology with κ-PVIIA.

**
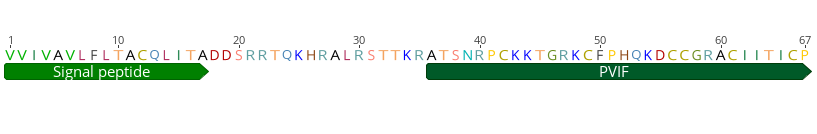
**

**
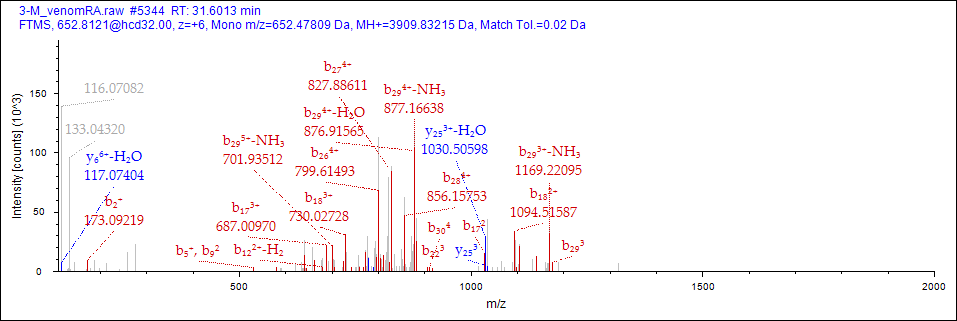
**

**Figure S23** PVIF 1) full transcript with annotated signal sequence and mature peptide regions and 2) annotated MS/MS spectra

**PVIG** is a new O1 Superfamily conotoxin. It closely resembles *C. purpurascens* nucleic acid sequences p2a (Sequence ID: AAQ05865), PVID (p2b, AAQ05866) and PVIF (p2c, AAQ05867) (15) (Figure SAlign). PVIG clustered with the lightning-strike cabal (Figure 3), although was only identified in one specimen (Figure 2). It does not share significant homology to any peptides with known activity, however it has the same cysteine framework and minimal homology to κ-PVIIA.


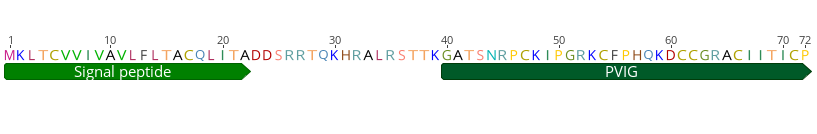


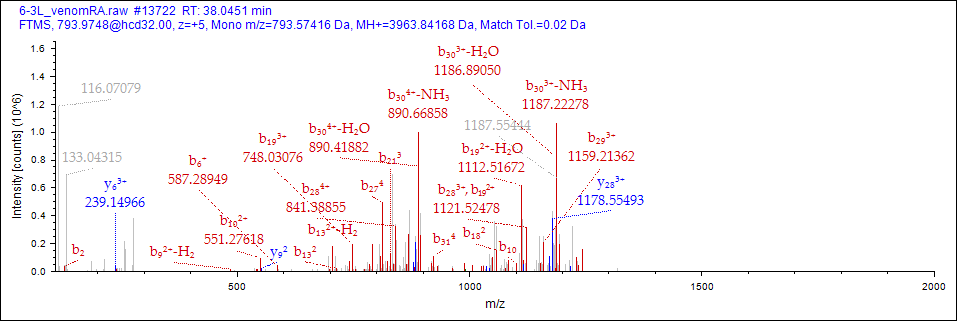


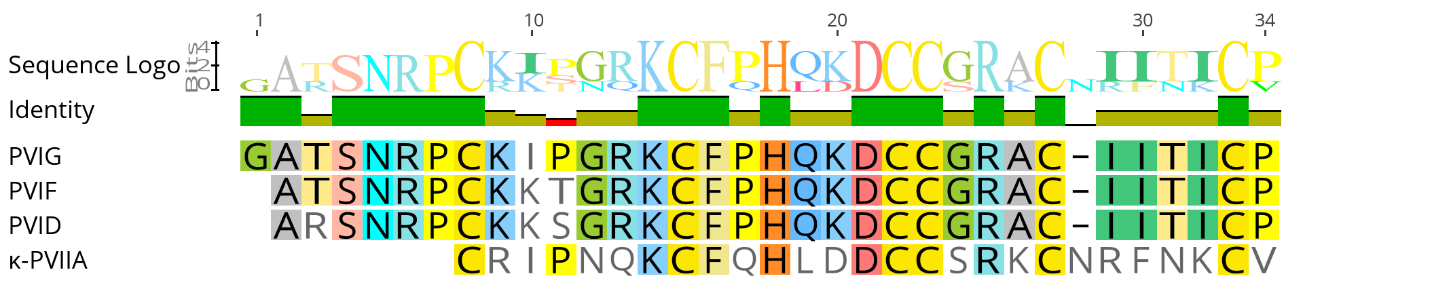


**Figure S24** PVIG 1) full transcript with annotated signal sequence and mature peptide regions, 2) annotated MS/MS spectra, and 3) sequence alignment with PVIF, and PVID, and κ-PVIIA.

**Framework VIII (C-C-C-CXC-CXC-CXCXC)**

**PVIIIA** belongs to the S Superfamily of conotoxins and contains 5 disulfide bonds. It was identified in 25 of the 27 venom samples, and therefore likely has an important role in the venom that has yet to be revealed. It clusters closely with ψ-PIIIE and α-PIVA and is a major component of the motor cabal. There are few framework VIII conotoxins with known bioactivity; two target the nAChR (α-GVIIIB, α-RVIIA) (16,17) and one targets the serotonin receptor (σ-GVIIIA) (18). However, PVIIIA does not display high sequence homology to any of these characterized toxins, aside from a conserved cysteine framework (Figure 5).


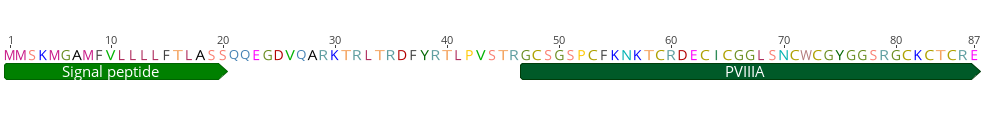


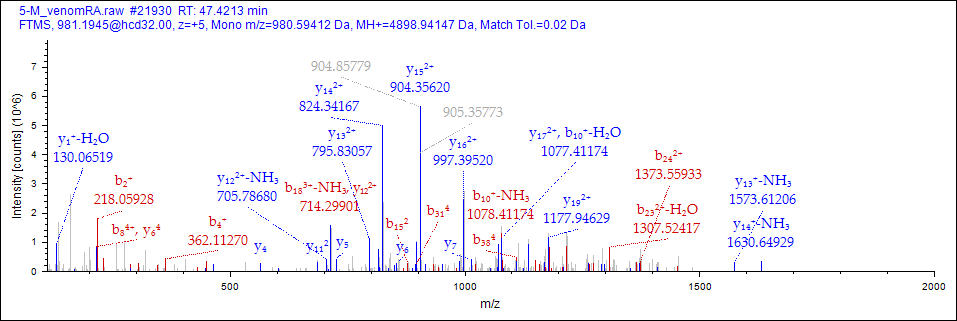


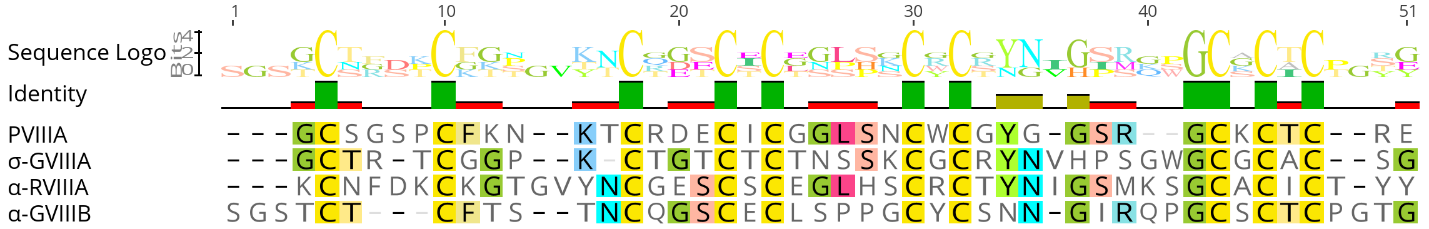


**Figure S25** PVIIIA 1) full transcript with annotated signal sequence and mature peptide regions, 2) annotated MS/MS spectra, and 3) sequence alignment with σ-GVIIIA, α-GVIIIB, and α-RVIIIA

**Framework XXI (CC-C-C-C-CC-C-C-C)**

**p21b** was expressed in transcriptome B and its precursor sequence classifies it as part of the con-ikot-ikot family. It shows 91% identity to p21a, a previously described 10 cysteine, 5-disulfide conotoxin (19). p21a was not expressed in either transcriptome and was not detected in the milked venom. However, p21b was identified in 10 of the 27 venom samples. p21b clusters closely to PVB and PIIIH, both newly described here. A con-ikot-ikot isolated from *C. striatus* targets the AMPA receptor and is presumed to contribute to the lightning strike cabal (20). In this study, p21b expression in the venom clusters with motor cabal toxins, suggesting an alternative molecular target for the con-ikot-ikot family of knottin peptides.


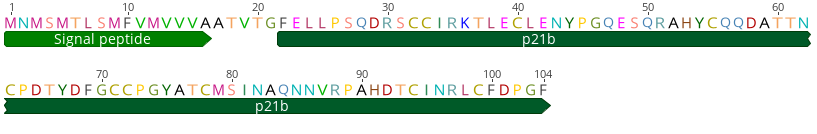


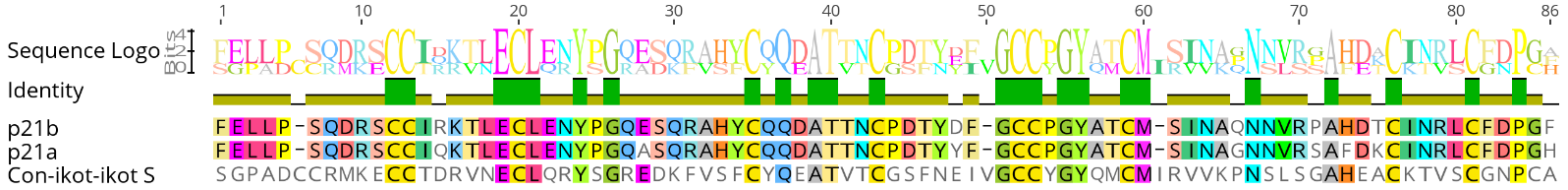


**Figure S26** p21b 1) full transcript with annotated signal sequence and mature peptide regions and 2) sequence alignment with p21a and con-ikot-ikot S

| Conopeptide | Master Protein Accession |
| --- | --- |
| α-PIA | P69658 |
| α-PIB | PIB |
| α-PIC | TRINITY_DN38298_c1_g1_i1_5 |
| PID | TRINITY_DN14184_c0_g1_i1_6 |
| PIE | TRINITY_DN69315_c2_g1_i1_2 |
| PIF | denovo2 |
| PIG | denovo6 |
| α-PIVA | TRINITY_DN72671_c7_g1_i2_2 |
| κ-PIVE | TRINITY_DN72671_c7_g1_i1_2 |
| κ-PIVF | PIVF |
| PIVH | TRINITY_DN38298_c0_g2_i1_3 |
| Linear-P | TRINITY_DN66702_c0_g1_i1_3 |
| Ile-Contryphan-P | TRINITY_DN2067_c1_g1_i1_5 |
| Contryphan-P3 | TRINITY_DN50122_c0_g1_i2_3 |
| Contryphan-P4 | denovo1 |
| ψ-PIIIE | TRINITY_DN77316_c0_g1_i1_2 |
| PIIIG | TRINITY_DN43886_c0_g1_i1_6 |
| PIIIH | TRINITY_DN50122_c0_g1_i1_3 |
| PIIII | TRINITY_DN72226_c3_g3_i1_6 |
| δ-PVIA | TRINITY_DN25228_c0_g1_i1_2 |
| PVIB | TRINITY_DN68626_c0_g1_i3_1 |
| PVIC | TRINITY_DN33623_c0_g1_i1_3 |
| PVID | TRINITY_DN34587_c0_g2_i2_1 |
| PVIE | TRINITY_DN40338_c0_g1_i1_6 |
| PVIF | Q71KS9 |
| PVIG | TRINITY_DN68626_c0_g1_i1_1 |
| κ-PVIIA | TRINITY_DN68626_c0_g1_i2_1 |
| Contryphan-P | TRINITY_DN69318_c0_g1_i1_2 |
| PIIA | TRINITY_DN61341_c0_g1_i1_5 |
| PVIIIA | TRINITY_DN38422_c2_g2_i1_1 |
| PVA | TRINITY_DN23996_c0_g1_i1_2 |
| PVB | TRINITY_DN55396_c0_g1_i1_1 |
| p21b | TRINITY_DN70906_c0_g1_i1_4 |

**Table S39-** Master protein accession IDs for all 33 identified base conopeptides. These IDs are used to reference conopeptides in the deposited Proteome Discoverer results files. Accession IDS beginning with TRINITY came from our venom duct transcriptome assembly. Accession IDS without ‘TRINITY’ are either UniProt entries or manual entries to our search database.

**References**

1. Abalde, S., Tenorio, M. J., Afonso, C. M. L., and Zardoya, R. (2018) Conotoxin Diversity in *Chelyconus ermineus* (Born, 1778) and the Convergent Origin of Piscivory in the Atlantic and Indo-Pacific Cones. *Genome Biol. Evol* **10**, 2643-2662

2. Lebbe, E. K., and Tytgat, J. (2016) In the picture: disulfide-poor conopeptides, a class of pharmacologically interesting compounds. *J. Venom. Anim. Toxins Incl. Trop. Dis.* **22**, 30

3. Dowell, C., Olivera, B. M., Garrett, J. E., Staheli, S. T., Watkins, M., Kuryatov, A., Yoshikami, D., Lindstrom, J. M., and McIntosh, J. M. (2003) α-Conotoxin PIA Is Selective for α6 Subunit-Containing Nicotinic Acetylcholine Receptors. *J. Neurosci.* **23**, 8445-8452

4. Nicke, A., Loughnan, M. L., Millard, E. L., Alewood, P. F., Adams, D. J., Daly, N. L., Craik, D. J., and Lewis, R. J. (2003) Isolation, structure, and activity of GID, a novel alpha 4/7-conotoxin with an extended N-terminal sequence. *J. Biol. Chem.* **278**, 3137-3144

5. Chi, S. W., Kim, D. H., Olivera, B. M., McIntosh, J. M., and Han, K. H. (2004) Solution conformation of alpha-conotoxin GIC, a novel potent antagonist of alpha3beta2 nicotinic acetylcholine receptors. *Biochem. J.* **380**, 347-352

6. Quinton, L., Servent, D., Girard, E., Molgo, J., Le Caer, J. P., Malosse, C., Haidar el, A., Lecoq, A., Gilles, N., and Chamot-Rooke, J. (2013) Identification and functional characterization of a novel alpha-conotoxin (EIIA) from *Conus ermineus*. *Anal. Bioanal. Chem.* **405**, 5341-5351

7. Lopez-Vera, E., Jacobsen, R. B., Ellison, M., Olivera, B. M., and Teichert, R. W. (2007) A novel alpha conotoxin (alpha-PIB) isolated from *C. purpurascens* is selective for skeletal muscle nicotinic acetylcholine receptors. *Toxicon* **49**, 1193-1199

8. Jacob, R. B., and McDougal, O. M. (2010) The M-superfamily of conotoxins: a review. *Cell. Mol. Life Sci.* **67**, 17-27

9. Franco, A., Dovell, S., Moller, C., Grandal, M., Clark, E., and Mari, F. (2018) Structural plasticity of mini-M conotoxins - expression of all mini-M subtypes by *Conus regius*. *FEBS J.* **285**, 887-902

10. Pardos-Blas, J. R., Irisarri, I., Abalde, S., Tenorio, M. J., and Zardoya, R. (2019) Conotoxin Diversity in the Venom Gland Transcriptome of the Magician's Cone, *Pionoconus magus*. *Mar. Drugs* **17**, 553

11. Li, Q., Barghi, N., Lu, A., Fedosov, A. E., Bandyopadhyay, P. K., Lluisma, A. O., Concepcion, G. P., Yandell, M., Olivera, B. M., and Safavi-Hemami, H. (2017) Divergence of the Venom Exogene Repertoire in Two Sister Species of Turriconus. *Genome Biol. Evol.* **9**, 2211-2225

12. Teichert, R. W., Jacobsen, R., Terlau, H., Yoshikami, D., and Olivera, B. M. (2007) Discovery and characterization of the short kappaA-conotoxins: a novel subfamily of excitatory conotoxins. *Toxicon* **49**, 318-328

13. Petrel, C., Hocking, H. G., Reynaud, M., Upert, G., Favreau, P., Biass, D., Paolini-Bertrand, M., Peigneur, S., Tytgat, J., Gilles, N., Hartley, O., Boelens, R., Stocklin, R., and Servent, D. (2013) Identification, structural and pharmacological characterization of tau-CnVA, a conopeptide that selectively interacts with somatostatin sst3 receptor. *Biochem. Pharmacol.* **85**, 1663-1671

14. Shon, K. J., Grilley, M. M., Marsh, M., Yoshikami, D., Hall, A. R., Kurz, B., Gray, W. R., Imperial, J. S., Hillyard, D. R., and Olivera, B. M. (1995) Purification, characterization, synthesis, and cloning of the lockjaw peptide from *Conus purpurascens* venom. *Biochemistry* **34**, 4913-4918

15. Duda, T. F., Jr., and Palumbi, S. R. (2004) Gene expression and feeding ecology: evolution of piscivory in the venomous gastropod genus *Conus*. *Proc Biol Sci* **271**, 1165-1174

16. Teichert, R. W., Jimenez, E. C., and Olivera, B. M. (2005) Alpha S-conotoxin RVIIIA: a structurally unique conotoxin that broadly targets nicotinic acetylcholine receptors. *Biochemistry* **44**, 7897-7902

17. Christensen, S. B., Bandyopadhyay, P. K., Olivera, B. M., and McIntosh, J. M. (2015) alphaS-conotoxin GVIIIB potently and selectively blocks alpha9alpha10 nicotinic acetylcholine receptors. *Biochem. Pharmacol.* **96**, 349-356

18. England, L. J., Imperial, J., Jacobsen, R., Craig, A. G., Gulyas, J., Akhtar, M., Rivier, J., Julius, D., and Olivera, B. M. (1998) Inactivation of a serotonin-gated ion channel by a polypeptide toxin from marine snails. *Science* **281**, 575-578

19. Moller, C., and Mari, F. (2011) 9.3 KDa components of the injected venom of *Conus purpurascens* define a new five-disulfide conotoxin framework. *Biopolymers* **96**, 158-165

20. Walker, C. S., Jensen, S., Ellison, M., Matta, J. A., Lee, W. Y., Imperial, J. S., Duclos, N., Brockie, P. J., Madsen, D. M., Isaac, J. T., Olivera, B., and Maricq, A. V. (2009) A novel *Conus* snail polypeptide causes excitotoxicity by blocking desensitization of AMPA receptors. *Curr. Biol.* **19**, 900-908
